# The STAT3 HIES mutation is a gain-of-function mutation that activates genes via AGG-element carrying promoters

**DOI:** 10.1093/nar/gkv911

**Published:** 2015-10-10

**Authors:** Li Xu, Jin-Jun Ji, Wangping Le, Yan S. Xu, Dandan Dou, Jieli Pan, Yifeng Jiao, Tianfei Zhong, Dehong Wu, Yumei Wang, Chengping Wen, Guan-Qun Xie, Feng Yao, Heng Zhao, Yong-Sheng Fan, Y. Eugene Chin

**Affiliations:** 1 Department of Immune Diseases, School of Basic Medicine, Zhejiang Chinese Medical University, Hangzhou, Zhejiang 310053, China; 2 Key Laboratory of Stem Cell Biology, Institute of Health Sciences, Shanghai Institutes for Biological Sciences, Chinese Academy of Sciences-Jiaotong University School of Medicine, 320 Yueyang Road, Shanghai 200031, China; 3 Cancer Center, School of Medicine Shandong University, Culture West Street, Jinan, Shandong 250012, China; 4 Department of Thoracic Surgery, Shanghai Chest Hospital, Shanghai Jiaotong University, 241 West Huaihai Road, Shanghai 200030,China

## Abstract

Cytokine or growth factor activated STAT3 undergoes multiple post-translational modifications, dimerization and translocation into nuclei, where it binds to serum-inducible element (SIE, ‘TTC(N3)GAA’)-bearing promoters to activate transcription. The STAT3 DNA binding domain (DBD, 320–494) mutation in hyper immunoglobulin E syndrome (HIES), called the HIES mutation (R382Q, R382W or V463Δ), which elevates IgE synthesis, inhibits SIE binding activity and sensitizes genes such as TNF-α for expression. However, the mechanism by which the HIES mutation sensitizes STAT3 in gene induction remains elusive. Here, we report that STAT3 binds directly to the AGG-element with the consensus sequence ‘AGG(N3)AGG’. Surprisingly, the helical N-terminal region (1–355), rather than the canonical STAT3 DBD, is responsible for AGG-element binding. The HIES mutation markedly enhances STAT3 AGG-element binding and AGG-promoter activation activity. Thus, STAT3 is a dual specificity transcription factor that promotes gene expression not only via SIE- but also AGG-promoter activity.

## INTRODUCTION

STAT3, known as the acute-phase response element, was first described in hepatocytes (1,2) and can be activated by the interleukin-6 (IL-6) cytokine family, type I interferon and epidermal growth factor (EGF) (3,4). Structurally, the STAT3 protein can be divided into three major regions: the N-terminal region (1–355) of the N-terminal helical domain extending into the next coiled-coil domain, the central region (355–555) of the canonical DNA binding domain (DBD) and the C-terminal region (555–770) of the linker-SH2 domain, which extends into the transcription activation (TA) domain.

STAT3 C-terminal K685 acetylation and Y705/S727 phosphorylation are involved in C-terminal dimerization and enhance some formation. The repeating β-sheets of the DBD (320–494) recognize and bind the serum-induced element (SIE) with the consensus sequence of ‘TTC(N3)GAA’ (5). In this canonical pathway, the STAT3 homodimer binds the SIE-containing promoters for gene regulation. Interestingly, the critical residues of the STAT3 DBD that are responsible for SIE binding include those with negative charges (E434, E435, V461, V462, V463) (6). Moreover, STAT3 indirectly regulates other transcriptional elements by forming complexes with transcription factors such as NF-κB, androgen receptor, estrogen receptor, glucocorticoid receptor and Jun B (7,8).

The STAT N-terminal region contains four large α-helixes that can be post-translationally modified. Cytokine-activated STAT3 is acetylated and methylated within this region for optimal activation or stabilization (9,10). The STAT N-terminal region is involved in STAT tetramer formation, transcriptional regulation and sub-cellular translocation (11). STAT3 with a 150–163 residue deletion within the first α-helix fails to undergo nuclear translocation (12). STAT3 with an R214/R215A substitution is Y705-phosphorylated normally but fails to respond to EGF or IL-6 for transcriptional activation (13), suggesting that the STAT3 N-terminal region can function independently of the C-terminal region in gene regulation.

DBD mutations in STAT3 (i.e., R382, F384, R423, V463 and V637) are a major cause of hyperimmunoglobulin E syndrome (HIES) and unexpected hyper-TNF-α promoter activity (14). Mice with STAT3 conditionally knocked out in B cells display normal B cell development and T cell-dependent antibody responses (15), suggesting that the STAT3 HIES mutation does not directly affect T and B cell function in antibody generation. In patients with the STAT3 HIES mutation, the TNF-α level is two–three-fold higher in the supernatant of Lipopolysaccharide (LPS) stimulated peripheral blood mononuclear cell (16,17). Transgenic mice that express a V463 deletion STAT3 mutation recapitulate multiple aspects of HIES, including elevated serum IgE and a significant elevation of serum TNF-α level (18). However, the STAT3 HIES mutants lost their SIE binding activity and failed to respond to SIE-promoter activation. Although NF-κB activation by LPS is well established for the upregulation of cytokines including IL-6 and TNF-α (19), leptin-activated B cells secrete cytokines including TNF-α via STAT3 activation (20). The HIES mutation is therefore a loss-of-function mutation in terms of SIE binding activity, but again-of-function mutation in terms of TNF-α gene regulation.

In this study, we applied ChIP-cloning and ChIP-on-ChIP approaches to identify other STAT3 binding elements. While ChIP-on-ChIP analysis is based on hybridization to identify the peaks of tagged DNA sequences, ChIP-cloning approach is based upon transcription factor DNA binding sites of variable affinities provides information regarding the genome-wide distribution (21,22). We now report an AGG-element with the consensus sequence ‘AGG(N3)AGG’ as a novel DNA motif for STAT3 binding directly. The AGG-element is distributed in a variety of promoters, including the TNF-α gene promoter. Moreover, the helical N-terminal region of STAT3 is critical for AGG-element binding. Although STAT3 with the HIES mutation abolished SIE binding and SIE-dependent gene regulation, HIES mutation is more active in AGG-element promoter activation.

## MATERIALS AND METHODS

### Cell lines and reagents

HepG2, 293T and PC3 cells were obtained from the Shanghai Institutes for Biological Sciences (SIBS), Chinese Academy of Sciences (CAS, Shanghai). Cells were cultured in high glucose DMEM (C11995500BT, Life Technologies) containing 10% fetal bovine serum (FBS) (10099–141, Life Technologies) with 100 units/ml penicillin and 10 μg/ml streptomycin (15140–122, Life Technologies) at 37°C in a 5% CO_2_ atmosphere.

The polyclonal antibody against STAT3 C-20 (sc-482) and monoclonal antibodies for Myc (sc-40) and pY20 (sc-508) were from Santa Cruz Biotechnology, Inc. The GFP monoclonal antibody (11814460001) was from Roche and the polyclonal antibody for acetyl-lysine (#9441s) was from Cell Signaling Technology. The secondary antibodies, including goat anti-rabbit RDye^®^ 680RD (926–68071) and goat anti-mouse RDye^®^ 800CW (926–32210), were from LICOR. Anti-pY45-STAT3 and anti-acetyl-K78-STAT3 polyclonal antibodies were prepared by AB-land, Inc. (Hangzhou, China).

Recombinant human IL-6 was from Life Technologies, LPS was from Sigma and recombinant human LIF was from Millipore. The dual-luciferase reporter assay system kit (E1910) was obtained from Promega.

### ChIP assays

- ChIP experiment: chromatin preparation and immunoprecipitation experiments were carried out as described previously (23). In brief, 2 × 10^7^ HepG2 cells in a 6-well plate were treated with IL-6 for 30 min and fixed in 1% formaldehyde. Chromatin was sheared into 600-bp fragments with sonication. Nuclear lysates prepared from these cells were incubated with anti-STAT3 (5 μg) for immune-precipitation and ChIP analysis.
- ChIP-cloning: The ChIP precipitates were amplified by polymerase chain reaction (PCR) using six base random primers according to the Whole Genome Amplification Kit (WGA1–50RXN; Sigma). The PCR-amplified gene products were then inserted into a pMD^®^18-T Vector (D101A; Takara Bio) and transformed into competent bacteria. For sequencing, 200 colonies were selected. ChIP-on-ChIP experiments were performed following the KangChen Biotech protocol (Shanghai).
- ChIP-PCR analysis: IL-6-treated HepG2 cells were used for the ChIP experiment. ChIP precipitates were used as PCR templates with nuclear lysates as input. PTK6, IL-6R, NTKR2 and EP300 were selected for ChIP-PCR. The nucleic acid sequences represented by the red boxes in Figure 7F were selected for ChIP-PCR. The correspond­ing primers were as follows:

PTK6-F,5-CCAGCACTTTGGGAAGC-3;

PTK6-R, 5-GGAGTCTCGCTCTGTCG-3;

NTRK-F,5-TGCTTTCTGCTGGAAT-3;

NTRK-R, 5-TACAGGTGCGTGCTAC-3;

EP300-F,5-CTCACGCCTGTCATCCC-3;

EP300-R, 5-GCAACCTCTGCCTCCTG-3;

IL-6R-F,5-TGGCTGACACGGTGAAAC-3;

IL-6R-R, 5-AGGCTGGAGTGCAGTGGT-3

### Transient transfection and luciferase reporter assay

293T cells in 24-well plates (1 × 10^5^ cells per well) were cotransfected with 0.5 μg of firefly luciferase reporter, 0.02 μg of pRL-SV40 renilla luciferase reporter (Promega) and wild-type or mutant cMyc-STAT3 using Lipofectamine 2000 according to the manufacturer's instruction (Life Technologies) for 24 h followed by treatment with IL-6 (20 ng/ml), LPS (10 μg/ml), LIF (20 ng/ml) or no treatment. Twenty-four hours later, whole cell lysates were prepared for the luciferase reporter activity analysis using a Dual-Luciferase^®^ Reporter Assay System (Promega). The pRL-SV40 plasmid was used to normalize transfection efficiency. Data are presented as the mean ± SD of three experiments.

### Myc-tagged protein purified

293T cells were transiently transfected with Myc-STAT3 or Myc-STAT3 mutants using SuperFectin^™^ II DNA Transfect Reagent (PuFei 2012–100) for 36 h followed by IL-6 treatment for another 30 min. Whole cell lysates were prepared and Myc-STAT3 proteins were purified with extensive washes by using Pierce Mammalian C-Myc Tag IP/Co-IP Kit (Thermo Scintific, 23625). Such Myc-affinity purified STAT3 proteins were applied for electrophoretic mobility shift assay (EMSA) and DNA affinity binding assay.

### Electrophoretic mobility shift assay (EMSA) and DNA affinity binding assay

Nuclear lysates were prepared from HepG2 cells treated with or without IL-6 according to the instructions of a Nuclear and Cytoplasmic Protein Extraction Kit (#P0027; Beyotime). The 5′-biotin-labeled oligonucleotides (AGG-element) synthesized by Sangon Biotech Company were incubated with HepG2 cell nuclear protein or purified protein and DNA binding proteins were separated in the gel according to a Light Shift Chemiluminescent EMSA Kit (#20148; Thermo Fisher Scientific).

The DNA affinity binding assay was performed according to our previous publication (24). Briefly, The 5′-biotin-labeled oligonucleotides were incubated with Dynabeads^®^ M-280 Streptavidin (#11205D Life Technologies) at room temperature for 30 min, washed three times with 1× B&W buffer (2× B&W buffer: 10 m MTris–HCl, pH 7.5, 2 M NaCl and 1 mM EDTA) and resuspended in DNA binding buffer (20 mM Hepes, pH 7.9, 100 mM KCl, 5 mM MgCl2, 0.1 mM EDTA, 4 mM DTT and 12% glycerol). An equal volume of cell lysate was then added and the sample was incubated for 60 min at room temperature, washed three times with DNA binding buffer and boiled for 10 min in sodium dodecyl sulphate (SDS) sample loading buffer (62.5 mM Tris, pH 6.8, 2% SDS, 10% glycerol, 1% Bromine phenol blue and 5% β-mercaptoethanol). The elution was then separated by 10% sodium dodecyl sulphate-polyacrylamide gel electrophoresis and immunoblotted with anti-STAT3 or anti-Myc.

### Immunoblotting and immunoprecipitation analysis

293T transfectants were harvested in ice-cold RIPA lysis buffer containing protease inhibitors and incubated for 60 min at 4°C with spinning. Cell lysates were cleared by centrifugation at 14 000 rpm for 5 min at 4°C. The supernatants were incubated with 1 μg of primary antibody overnight at 4°C followed by the addition of protein A/G beads (P2012, Beyotime Institute Biotechnology) and rotation for 3 h. The beads were collected and washed three times with 1 ml of RIPA lysis buffer and then denatured by boiling in SDS sample loading buffer. The supernatants were analyzed by western blot assay with different primary antibodies (anti-Myc, anti-GFP, anti-pTyr (PY20) or anti-acetyl-Lys). Proteins were visualized using the Odyssey Infrared Imaging System (LI-COR Biosciences) with the appropriate secondary antibodies as per the manufacturer's instructions.

### Plasmids and cDNA constructs

Nucleic acid sequences containing the 5′ ends of the nucleic acid fragment plus a HindIII recognition site and the 3′ end of sequences plus a SalI recognition site were generated, and three protective bases were added to both ends. The nucleic acid sequence and its complementary sequence were diluted with STE buffer (10 mM Tris, pH 8.0, 50 mM NaCl, 1 mM EDTA) and annealed. Double-stranded nucleic acid fragments were then digested with HindIII/SalI and subcloned into a luciferase reporter vector. The sequences used to generate luciferase vectors are shown in Supplementary Figure S1. Two deletion mutants based on the AGG-Luc vector (AGGΔ1-Luc and AGGΔ2-Luc) were constructed. Sequences used to generate the deletion mutation are as follows:

AGGΔ1-Luc F: TGAAAGGCTGTGCGCGCGAGGGAGGG;

AGGΔ1-Luc R: CCCTCCCTCGCGCGCACAGCCTTTCA;

AGGΔ2-Luc F: TGCGCGGAGGGCGGGGGCCGTCGACC;

AGGΔ2-Luc R: GGTCGACGGCCCCCGCCCTCCGCGCA

GFP-tagged STAT3 and truncated STAT3 were constructed using the corresponding Myc-STAT3 expression vector. Myc-STAT3 and truncated STAT3 expression vectors were digested with EcoRI/ApaI, and the target fragments were subcloned into the pEGFP-C1 vector. The deletion mutation (170–180Δ) and other mutants (R382Q, R382W, E434/435A, V462/462/463A, R423Q, V463Δ, S465A) were constructed usinga QuikChange^®^ XL site-directed mutagenesis kit (200517, Agilent Technologies). The following primers were employed:

170–180ΔF, GTGGAGAACCTCCAGGACAGCCAAGGAGACATGCAG

170–180ΔR, CTGCATGTCTCCTTGGCTGTCCTGGAGGTTCTCCAC

E434/435A-F, TCCTTGATCGTGACTGCCGCCCTGCACCTG

E434/435A-R, CAGGTGCAGGGCGGCAGTCACGATCAAGGA

V461/462/463A-F, TCCTTGCCAGCCGCCGCCATCTCCAACATC

V461/462/463A-R, GATGTTGGAGATGGCGGCGGCTGGCAAGGA

R382Q-F, CAGAGGGTCTCAGAAATTTAACAT

R382Q-R, ATGTTAAATTTCTGAGACCCTCTG

R382W-F, CAGAGGGTCTTGGAAATTTAACAT

R382W-R, ATGTTAAATTTCCAAGACCCTCTG

R423Q-F, GGGAATGGAGGCCAGGCCAATTGT

R423Q-R, ACAATTGGCCTGGCCTCCATTCCC

V463Δ-F, TTGCCAGTTGTGATCTCCAACATCT

V463Δ-R, AGATGTTGGAGATCACAACTGGCAA

S465A-F, TTGTGGTGATCGCCAACATCTGTCA

S465A-R, TGACAGATGTTGGCGATCACCACAA

Genomic DNA was extracted, and the corresponding primers were synthesized. We then amplified the TNF-α promoter region. The nucleic acid amplification sequences are shown in Supplementary Figure S2A. The PCR products were digested with HindIII/SalI and inserted into the luciferase plasmid as TNF-Luc. The following primers were utilized:

TNFα-promoter F, CCCAAGCTTCCAGGGCTATGGAAGTCG

TNFα-promoter R, ACGCGTCGACGAGAAGAGGCTGAGGAACAAG

Two deletion mutants based on the TNF-Luc vector were constructed(TNFΔ1-Luc and TNFΔ2-Luc). The following primers were employed:

TNFΔ1-Luc -F: GGAGGCAATAGGTTTTCAGTTGTTGGCACACC

TNFΔ1-Luc -R: GGTGTGCCAACAACTGAAAACCTATTGCCTCC

TNFΔ2-Luc -F: AACTTTCCAAATCCCCCTCATGGGTTTCTCCA

TNFΔ2-Luc -R: TGGAGAAACCCATGAGGGGGATTTGGAAAGTT

All DNA sequencing was performed by the Sangon Biotech Company (Shanghai). The PCR cleanup kit (AP-PCR-50), plasmid miniprep kit (AP-MN-P-50) and DNA gel extraction kit (AP-GX-50) were from AxyGen Biosciences. A ligation high kit (LGK-101; Toyobo Co), high pure maxi plasmid kit (DP116; Tiangen Biotech) and TRIzol (15596–026; Life Technologies) were used. Dpn I (1233A), EcoRI (1040A), ApaI(1005A), HindIII (1060A), SalI (1080A), Takara Taq™ (DR001A), Takara LA Taq^®^ (DRR002A), Prime STAR^®^ Max DNA Polymerase (DR045S), Prime Script RT-PCR kit (DRR014S) and DNA mate (D605A) were from TaKaRa Bio.

### RNA iolation and RT-PCR analysis

HepG2 cells were transiently transfected with Myc-STAT3, wild-type STAT3 or HIES mutant STAT3 as previously described, followed by LPS or IL-6 treatment. Total RNA was extracted with TRIzol (Life Technologies) according to the manufacturer's instructions. Reverse transcription was performed with oligodT primers and reverse transcriptase M-MLV (#D2640A; Takara). Real-time PCR was carried out in a Bio-RAD-IQ5 Multicolor-Real-Time PCR system with SYBR Green PCR Master Mix (TaKaRa #DRR041A). Relative levels of gene expression were determined with β-actin as the control. RT-PCR was carried out using a Bio-Rad T100 Thermal Cycler PCR system with β-actin as the control. The following primers were used for gene amplification:

TNF-α-F, GTCTGGGCAGGTCTACTTT

TNF-α-R, TGGGCTCCGTGTCTCA

PTK6-F, CGGGAGACTGACACGAAGC

PTK6-R, CCAGCGACAAAGGTGAAGG

EP300-F, GCCAGTGTCGGAATGCCAATT

EP300-R, GCTGAGCAGTCTGAGGAGGG

IL-6R-F, CTGGGTGCTCAGGAATCAGG

IL-6R-R, GTTGGCGACGCATAGGG

NTRK2-F, ATGGCTTTCTAGTCTTGTGG

NTRK2-R, GCATACTCGGCTCTTTGA

β-actin-F, ATGCCATCCTGCGTCTG

β-actin-R, ATGCCATCCTGCGTCTG

## RESULTS

### STAT3 binds to the AGG-element

**Figure 1.**
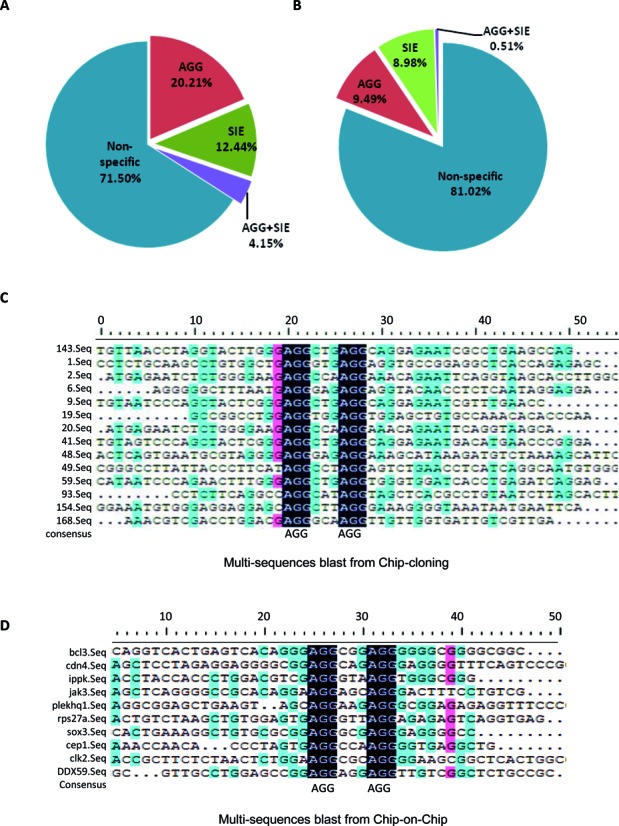
Screening different STAT3 DNA-binding sequences. (**A**) The relative ratio of various sequences from the ChIP-cloning results. (**B**) The relative ratio of various sequences from the ChIP-on-ChIP results. (**C**) The AGG sequence alignment analysis. The sequences were derived from the ChIP-cloning results. (**D**) The AGG sequence alignment analysis. The sequences were derived from the ChIP-on-Chip results.

To uncover novel STAT3-promoter binding activities, we performed ChIP cloning and ChIP-on-ChIP experiments in HepG2 cells. In addition to non-specific binding sequences, many DNA samples recovered from the STAT3 immuno-precipitates contained the so-called AGG-motif with the consensus sequence ‘AGG(N3)AGG’. From a careful analysis of 194 sequences obtained from STAT3-ChIP cloning results, 20.21% of the DNA sequences contained the AGG motif, whereas only 12.44% DNA sequences contained the SIE motif (Figure 1A). Similarly, in the ChIP-on-ChIP results of 594 putative STAT3 binding sequences, AGG motifs (9.49%) were slightly more represented than SIE motifs (8.98%) (Figure 1B). SIE-sequences contain TTC and GAA duplicates typically separated by three bases (5′-TTC(N3)GAA-3′), AGG-sequences contain AGG duplicates that are also separated by three bases of any sequence (Figure 1C and D).

**Figure 2.**
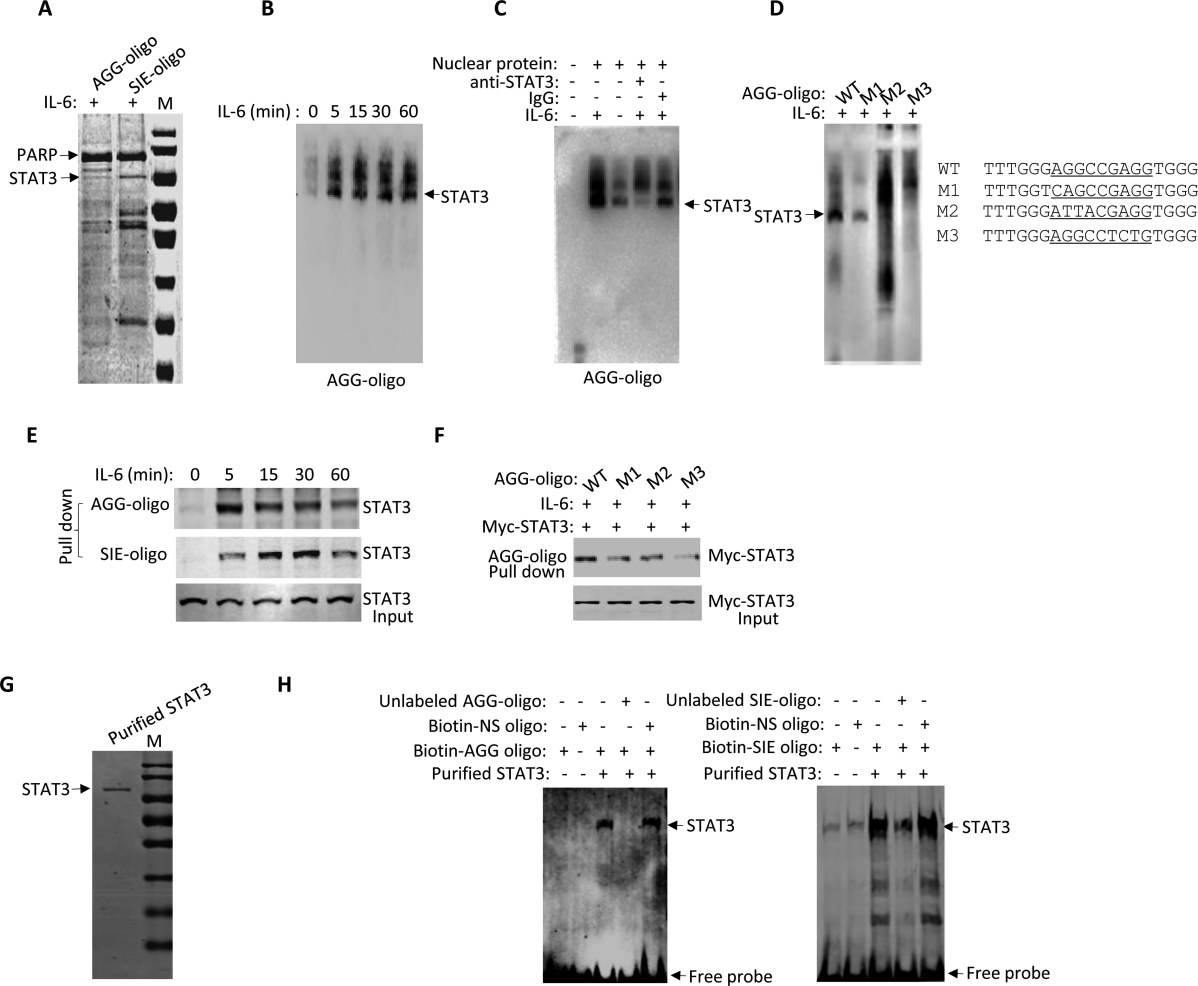
STAT3 binds to the AGG-motif directly. (**A**) Nuclear proteins from IL-6 (20 ng/ml)-treated HepG2 cells were incubated with biotinylated SIE or AGG-element oligonucleotidebeads for the DNA affinity binding experiment. DNA pull down samples were separated with 10% sodium dodecyl sulphate-polyacrylamide gel electrophoresis (SDS-PAGE) and stained with Coomassie blue. All of the proteins were trypsinized for mass spectrometry analysis. Mass spectrometry identified STAT3 as indicated. (**B**) Nuclear proteins of IL-6-treated HepG2 cells were incubated with a 5′-biotinyl-AGG oligonucleotide probe for 30 min and submitted to an electrophoretic mobility shift assay (EMSA). (**C**) Nuclear proteins of HepG2 cells treated with or without IL-6 were incubated with5′-biotinyl-AGG oligonucleotide probe prior to an EMSA assay. STAT3 antibody and control IgG were used for competition reactions. STAT3–DNA complexes were as indicated. (**D**) Nuclear proteins of IL-6-treated HepG2 cells were incubated with a 5′-biotinyl-AGG (mutation) oligonucleotide probe prior to an EMSA. STAT3–DNA complexes were as indicated. (**E**) Whole cell lysates of HepG2 cells were incubated with SIE or AGG oligonucleotide beads prior to a DNA affinity binding assay. The DNA oligonucleotide pull down proteins were then separated via 10% SDS-PAGE followed by anti-STAT3 immunoblotting. (**F**) HepG2 cells were transiently transfected with Myc-STAT3 for 36 h followed by IL-6 treatment for 30 min. Whole cell lysates prepared from the cells were then incubated with AGG or AGG mutant oligo-beads for affinity precipitation analysis and 10% SDS-PAGE prior to immunoblotting with anti-Myc. (**G**) Myc-tagged STAT3-wild-type was transfected into 293T cells for 36 h followed by IL-6 treatment for additional 30 min. Whole cell lysate was prepared from the cells and the Myc-tagged STAT3 proteins were purified with c-Myc Tag IP/Co-IP Kit and separated with 10% SDS-PAGE with Coomassie brilliant blue staining. (**H**) Above purified Myc-tagged STAT3 proteins (2 µg) and biotin-AGG-oligo or SIE-oligo probe (1 nM) were used for EMSA assay. Also included here were 200-fold unlabeled SIE oligo probe, AGG-oligo or biotin-non-specific oligo as indicated.

To investigate STAT3 binding capacity to the AGG-element, we performed DNA oligonucleotide affinity precipitation and mass spectrometry analysis. STAT3 was identified among the proteins pulled down by both the AGG-oligonucleotide beads and the SIE-oligonucleotide beads as the positive control. Mass spectrometric analysis clearly revealed that STAT3 was efficiently pulled down by both the SIE-oligonucleotide beads and the AGG-oligonucleotide beads (Figure 2A). To confirm that STAT3 proteins directly bind to the AGG-oligonucleotides, we synthesized biotin-labeled probes with the AGG-sequences and performed an EMSA. The nuclear fraction prepared from HepG2 cells was incubated with the AGG-probes. EMSA revealed a typical STAT-DNA binding pattern of three bands of STAT homo- and heterodimers that were induced by IL-6 treatment for 5 min and lasted up to 60 min (Figure 2B). To distinguish which of the three bands represented STAT3, we included an antibody against STAT3 in the reaction. The bottom band formed upon IL-6 treatment was clearly disrupted by the STAT3 antibody but not by the control IgG antibody (Figure 2C), suggesting that this bottom band most likely represents the AGG-probe associated STAT3 homodimer.

To provide additional evidence of the specificity of STAT3-AGG-element binding, we mutated the flanking AGG sequences of the probes. The three mutations, designated M1, M2 and M3, are listed in the right panel of Figure 2D. These AGG-oligonucleotide mutants carried mutations in the flanking AGG sequences without changing the internal base (N3) sequence. While all of the AGG mutated probes reduced STAT3 binding activity, the M3 mutation completely abolished STAT3 binding in the EMSA (Figure 2D, left panel). We also applied a DNA binding affinity pull down approach to demonstrate STAT3 binding to the AGG-element. In the DNA pull down assay, IL-6 activated STAT3 proteins prepared from HepG2 nuclear fractions were precipitated by AGG-oligonucleotide beads as well as SIE-oligonucleotide beads as the positive control (Figure 2E). As expected, the M1, M2 and M3 AGG-mutant oligonucleotides attenuated the STAT3 binding activity (Figure 2F).

To confirm the direct binding activity of STAT3 for AGG element, we performed EMSA by using purified STAT3 protein. Myc tagged STAT3 proteins were affinity purified from 293T transfectants treated with IL-6. After extensive washes, Myc affinity purified STAT3 protein was fairly pure with no visible contamination of other proteins (Figure 2G). We then incubated such purified STAT3 protein with biotin-labeled AGG-oligo (Figure 2H, left panel) or SIE-oligo (Figure 2H, right panel) for EMSA. Apparently, purified STAT3 protein could bind to both SIE and AGG probes without apparent difference and this binding reaction can be prevented by competition from both unlabeled SIE-olgio or AGG-oligo but not Biotin-labeled non-specific oligo (Figure 2H). STAT3 binding to the AGG-oligo was visualized with elongated exposure time, supporting the notion that STAT3 binding to the SIE sequence more efficiently than binding to the AGG sequence. Thereby, we conclude that the ‘AGG(N3)AGG’ sequence (AGG element) represents a novel STAT3 binding DNA element and STAT3 can bind to this sequence directly.

### The STAT3 N-terminal region is post-translationally modified for AGG-element-dependent transcriptional activation

**Figure 3.**
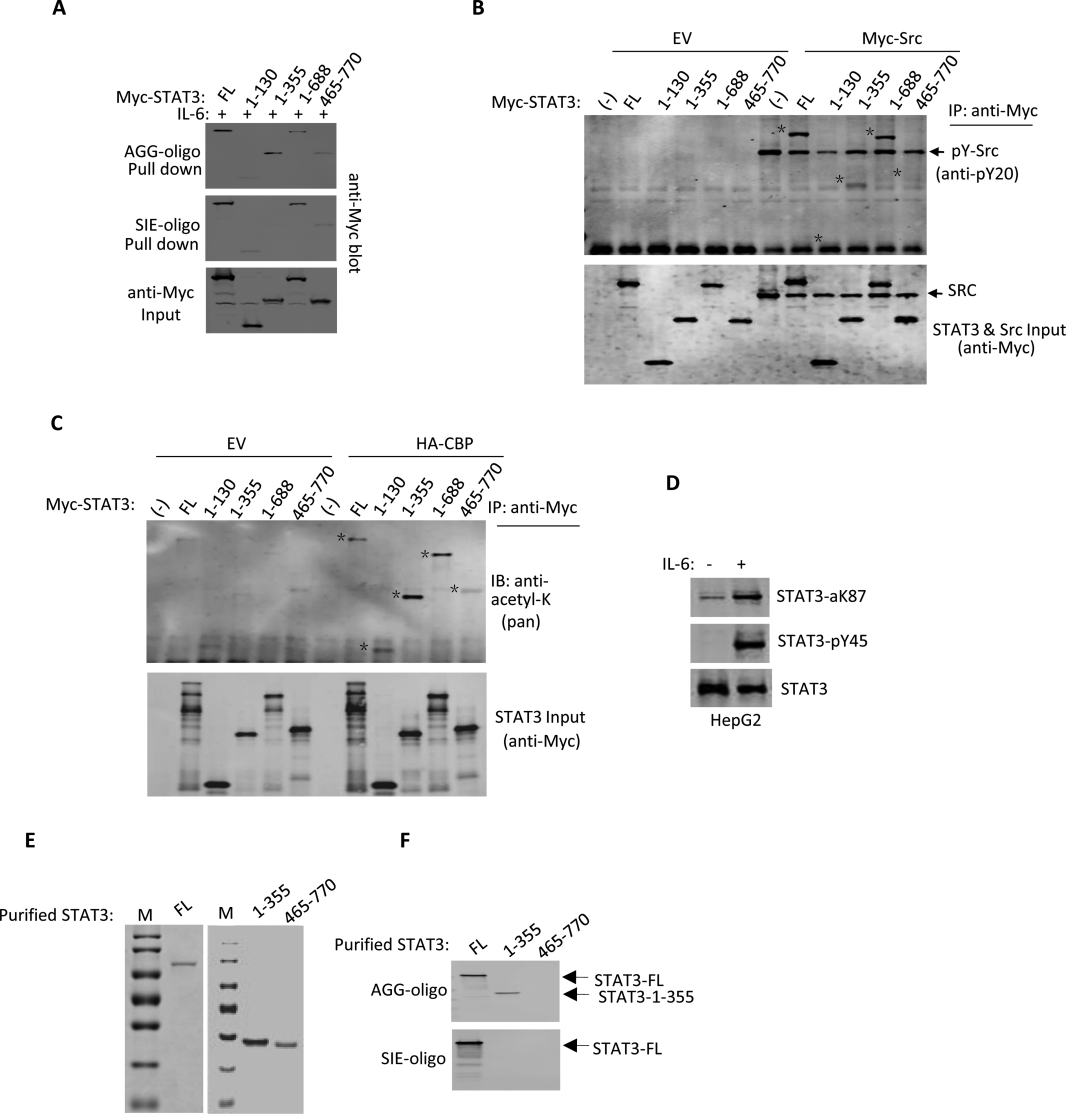
The STAT3-N domain undergoes post-translational modifications. (**A**) Myc-tagged STAT3-full length (FL) or indicated STAT3 domains were transfected into 293T cells. After 36 h, the whole cell lysates were used for AGG or SIE oligonucleotide bead pull down experiments. The pull down samples were detected by immunoblotting with anti-Myc, and whole cell lysates were used as the input. (**B**) Myc tagged STAT3-FL or indicated STAT3 domains were transfected with Myc-tagged c-Src or EV (empty vector) into 293T cells. After transfection for 36 h, the whole cell lysates were used for immunoprecipitation with the Myc antibody. STAT3 tyrosine phosphorylation was detected with a pan phosphotyrosine antibody (anti-PY20). Whole cell lysates were used as the input. (**C**) Myc-tagged STAT3-FL or indicated STAT3 domains were transfected with or without HA-tagged CBP into 293T cells. After transfection for 36 h, the whole cell lysates were used for immunoprecipitation with the Myc antibody. STAT3 lysine acetylation was detected with a pan acetyllysine antibody. (**D**) Whole cell lysates of HepG2 cells were treated with or without IL-6 for 15 min for STAT3 analysis with specific antibodies against phospho-Y45 STAT3 and acetyl-K78 STAT3, as indicated. (**E**) Myc-tagged STAT3 FL, 1–355 and 460–770 regions were transfected into PC3 cells for 36 h followed by IL-6 treatment for 30 min. Whole cell lysates were prepared and the Myc-tagged STAT3 protein was purified with Myc Tag IP/Co-IP Kit and separated by 10% SDS-PAGE with Coomassie brilliant blue staining. (**F**) The above purified Myc-tagged STAT3 proteins were incubated with SIE-oligo or AGG-oligo beads for DNA affinity binding assay. After three extensive washes, oligo pull down STAT3 proteins were analyzed with anti-Myc blotting.

The central region of STAT3 contains the DBD responsible for SIE binding (5). To confirm whether the DBD was responsible for binding to both the SIE and AGG-elements, various STAT3 domain truncation proteins were constructed and compared for their binding to the AGG-element and the SIE using a DNA pull down experiment. While full length STAT3 was pulled down by both the SIE- and AGG-oligonucleotide beads, the STAT3 N-terminal region (1–355) was only pulled down by the AGG-oligonucleotide beads (Figure 3A). STAT3 bound much more effectively to the SIE-oligonucleotide beads when extended up to the SH2 domain (1–688) (Figure 3A). However, when STAT3 1–355 was shortened further, STAT3 1–130 failed to bind to the AGG-element (Figure 3A), suggesting that the N-terminal region 1–355 was necessary for optimal AGG-element binding activity.

STAT3 1–355 responded to IL-6 for the AGG-element binding, suggesting that the STAT3 N-terminal region might undergo post-translational modification upon IL-6 treatment. Post-translational modifications within the N-terminal domain of STAT3 have been reported (9,10,13). To examine tyrosine phosphorylation or acetylation of the STAT3 N-terminal domain, we cotransfected STAT3 truncates with c-Src or CBP in 293T cells. STAT3 1–355 tyrosine phosphorylation by c-Src was clearly detected with a pan tyrosine phosphorylation antibody (Figure 3B). STAT3 1–335 acetylation by CREB-binding protein (CBP) CBP was strikingly compared with the acetylation of full length STAT3 or STAT3 1–688 (Figure 3C). Multiple post-translational modifications of the STAT3 N-terminal domain were confirmed by specific antibodies for STAT3 Y45 phosphorylation and K87 acetylation in HepG2 cells upon IL-6 treatment (Figure 3D). In order to confirm the direct binding effect of the STAT3 N-domain protein for AGG-sequence, we purified both STAT3-N-terminal region (1–355) and STAT3-C-terminal region (465–770) proteins and performed DNA affinity binding assay. The results clearly showed that purified STAT3 (1–355) protein only bound with AGG-oligo, whereas the purified STAT3 (465–770) protein bound to neither AGG-oligo nor SIE-oligo (Figure 3E and F).

**Figure 4.**
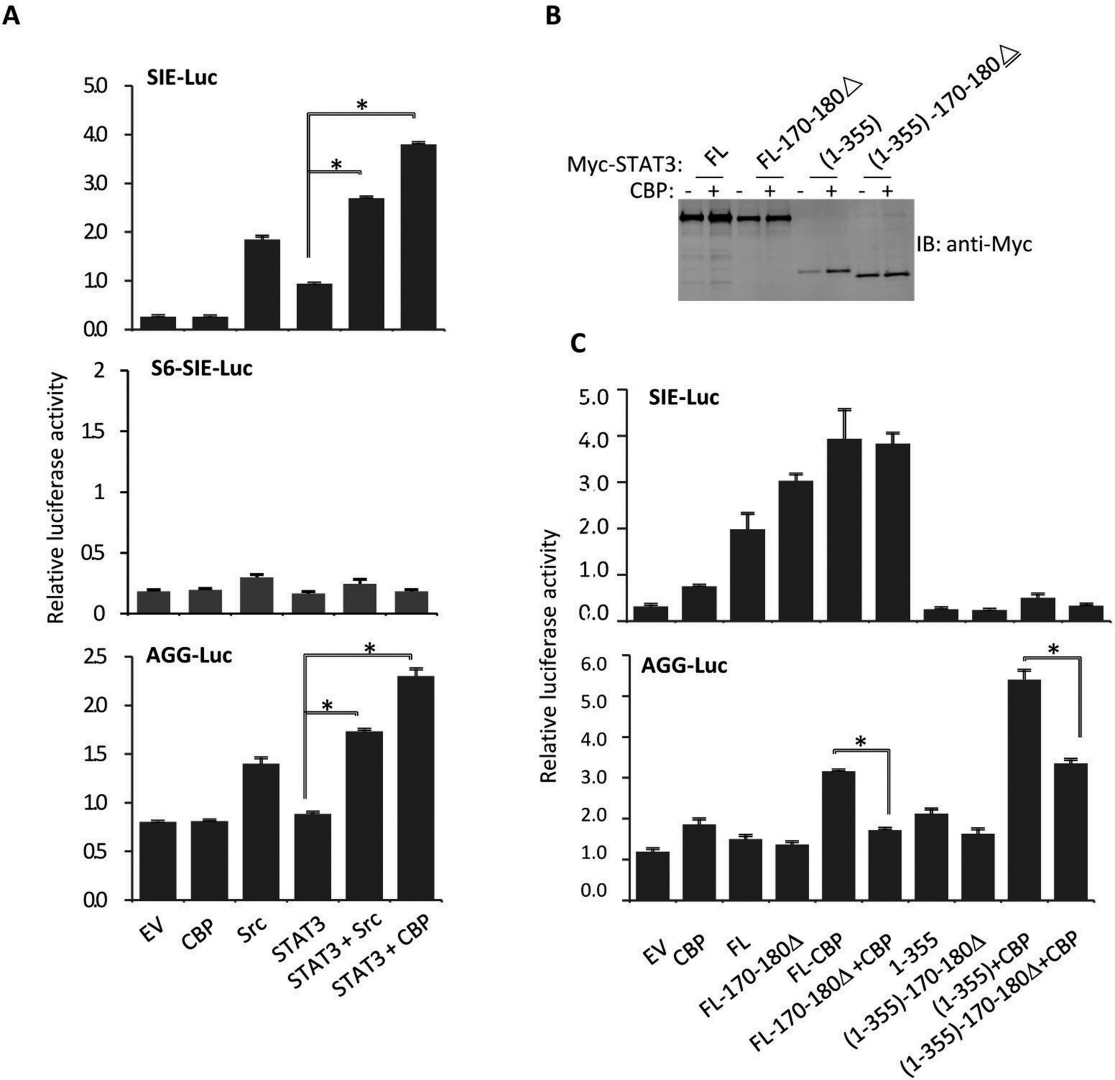
The STAT3-N domain is responsible for AGG-Luc promoter activation. (**A**) STAT3, CBP, c-Src and EV expression plasmids were transfected along with corresponding luciferase reporter vector into 293T cells. Luciferase activity was examined 48 h after transfection. Data are representative of three independent experiments, and bar graphs show the mean ± SD with *P < 0.01. (**B**) Myc-tagged STAT3-full length or indicated STAT3 domains were transfectedinto 293T cells. After 36 h, the cells were suspended in lysis buffer and samples were analyzed by anti-Mycimmunoblotting. (**C**) Myc-tagged STAT3-FL or indicated STAT3 domains were transfected along with AGG-Luc reporter or SIE-Luc reporter vectors in 293T cells followed by transfection with or without CBP. Luciferase activity was examined 48 h after transfection. Data are representative of three independent experiments, and bar graphs show the mean ± SD, with *P < 0.01.

To investigate STAT3 N-terminal region transcription activity, we performed a luciferase reporter activity assay by adopting three luciferase reporter systems: SIE-luciferase, STAT6 SIE-luciferase and AGG-luciferase. The AGG-luciferase reporters were constructed using the promoter sequences obtained from the ChIP-on-ChIP results (Supplementary Figure S1). STAT3 activation was monitored by including c-Src or CBP for cotransfection. As expected, STAT3 transcriptional activity was clearly detected by cotransfecting STAT3 with c-Src or CBP and the SIE-element reporter but not with the STAT6 SIE reporter, which contains one additional base between ‘TTC’ and ‘GAA’ (TTC(N4)GAA) (Figure 4A). When the AGG-luciferase reporter was tested, a similar pattern of STAT3 transcriptional activity was detected when STAT3 was transfected along with c-Src or CBP (Figure 4A, bottom). For both the SIE- and AGG-luciferase reporters, CBP was more active than c-Src in inducing STAT3 activation. We then compared the STAT3 N-terminal region 1–355 activity on both the SIE- and AGG-luciferase reporters. CBP cotransfection often enhances protein stability in cells, presumably via acetylation. When STAT3 full length or 1–355 was cotransfected with CBP, 1–355 accumulation was more obvious (Figure 4B). STAT3 1–355 transcriptional activity was enhanced by CBP cotransfection with the AGG-luciferase reporter but not with the SIE-luciferase reporter (Figure 4C).

To identify the motif critical for STAT3 1–355 transcriptional activity, we tested a series of STAT3 variants. Compared to STAT3 full length or 1–355, the deletion of STAT3 170–180 markedly abolished AGG-luciferase reporter activation in response to CBP cotransfection (Figure 4C). In contrast, STAT3 with the 170–180 deletion showed no obvious defect in SIE-luciferase reporter activation (Figure 4C). Therefore, either methylation and or acetylation of these residues (170–180) can be critical for STAT3 N-terminal activity in AGG-promoter regulation

### The intracellular localization of STAT3 truncations and mutants

**Figure 5.**
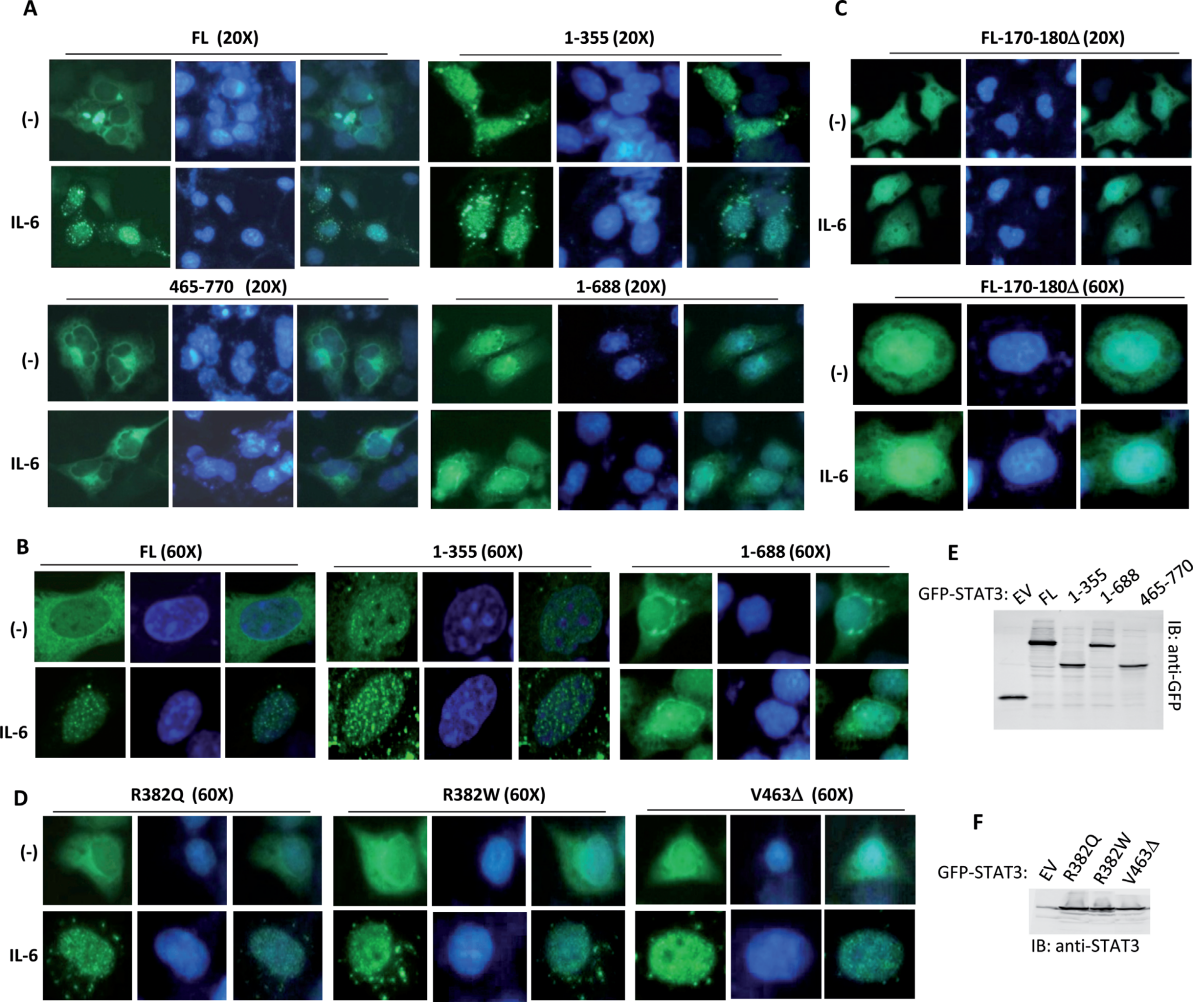
The intracellular localization of STAT3 truncations and mutants. (**A** and **B**) GFP-tagged STAT3-FL or indicated STAT3 domains were transfected into HepG2 cells. After transfection for 24 h, the cells were treated with or without IL-6 for 30 min and then fixed and visualized with a confocal microscope. Nuclei were stained with DAPI (blue). (**C** and **D**) GFP-tagged STAT3 mutants were transiently expressed in HepG2 cells. After transfection for 24 h, the cells were treated with or without IL-6 for 30 min and then fixed and visualized with a confocal microscope. Nuclear DNA was stained with DAPI (blue). (**E**) GFP-tagged STAT3-FL or indicated STAT3 domains were transfected in 293T cells. After transfection for 36 h, the cell lysates were detected by immunoblotting with anti-GFP antibody. (**F**) GFP-tagged STAT3-wild-type (WT) and mutants were transiently transfected into 293T cells. After 36 h, the cell lysates were detected by anti-STAT3 immunoblotting.

We analyzed the STAT3 N-terminal region for nuclear translocation. Punctate STAT3 nuclear distribution accompanied by CBP and CBP-mediated histone acetylation was previously reported as an indication of transcriptionally active chromatin. These punctate STAT3 nuclear distributions were defined as the STAT3-specific nuclear body by Herrman (25). Recently, technological advances in nuclear body are providing new insights into their roles in regulation of key cellular events and their contribution to disease progression (26). GFP- tagged STAT3 truncations were expressed in 293T or HepG2 cells (Figure 5A, B and E). Although the nuclear translocation of GFP-STAT3-full length required IL-6 treatment, STAT3 N-terminal region (1–355)-GFP was nuclear in HepG2 cells (Figure 5A and B). As expected, GFP-STAT3-full length showed punctate nuclear distribution upon IL-6 treatment (Figure 5B). Moreover, STAT3 1–355 also underwent punctate nuclear distribution upon IL-6 treatment, whereas STAT3 465–770 subcellular localization failed to respond to IL-6 (Figure 5A). The 170–180 deletion abolished the STAT3 punctate distribution pattern without affecting STAT3 nuclear translocation (Figure 5C). This finding was consistent with the above observation that the deletion of 170–180 from STAT3 markedly abolished STAT3 full length or 1–355 activity in response to CBP cotransfection in AGG-luciferase reporter activation (Figure 4C). STAT3 1–355 was far more active in response to IL-6 treatment than STAT3 1–688 for both nuclear translocation and the punctate distribution pattern (Figure 5B). These results together suggest that the N-terminal region alone is sufficient for STAT3 nuclear translocation and transcriptionally active nuclear distribution.

### The HIES mutation is gain-of-function in AGG-element-dependent transcription regulation

**Figure 6.**
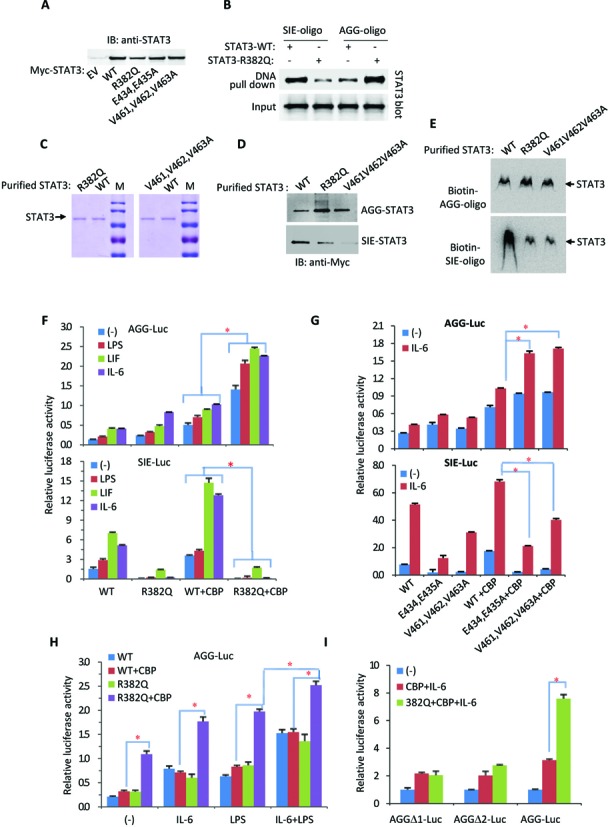
The STAT3 HIES mutation is gain-of-function in AGG-promoter activation. (**A**) Myc-tagged wild-type (WT) and mutant STAT3 were transiently transfected into 293T cells. After 36 h, the cell lysates were detected by anti-STAT3 immunoblotting. (**B**) Myc-STAT3 or R382Q mutant were transiently transfected into STAT3-null cells (PC3) for 36 h followed by IL-6 treatment for additional 30 min. The cell lysates were incubated with SIE or AGG oligonucleotide beads for the DNA affinity binding assay, and the pulled-down samples were analyzed by immunoblotting with a Myc antibody. The whole cell lysates were used as the input. (**C**) Myc-tagged STAT3 wild-type and mutants were transfected into 293T cells for 36 h followed by IL-6 treatment for 30 min. Whole cell lysates were prepared and the Myc-tagged STAT3 protein was purified with Myc Tag IP/Co-IP Kit and separated by 10% SDS-PAGE with Coomassie brilliant blue staining. (**D**) The above purified Myc-tagged wild-type STAT3 or STAT3 mutant proteins were incubated with SIE-oligo or AGG-oligo beads for 30 min on ice. After three extensive washes, STAT3 proteins pulled-down with oligo-beads were analyzed with immunoblotting. (**E**) The above purified wild-type STAT3 or STAT3 mutant proteins (2 µg) and Biotin-SIE or Biotin-AGG oligon probe (1 nM) were used for EMSA assay. (**F**) The STAT3-WT or -R382Q mutant were transfected along with AGG-Luc reporter vectors or SIE-Luc reporter vectors into 293T cells followed by transfection with or without CBP. After transfection for 24 h, the cells were treated with IL-6 (20 ng/ml), LPS (10 μg/ml) or LIF (20 ng/ml) for an additional 24 h prior to luciferase reporter analysis. Data are representative of three independent experiments, and bar graphs show the mean ± SD, with *P < 0.01. (**G**) STAT3-WT or E434/435A and V461/462/463A mutants were transfected along with AGG-Luc reporter vectors or SIE-Luc reporter vectors into 293T cells followed by transfection with or without CBP. After transfection for 24 h, the cells were treated with IL-6 for an additional 24 h prior to luciferase reporter analysis. Data are representative of three independent experiments, and bar graphs show the mean ± SD, with *P < 0.01. (**H**) STAT3-WT or STAT3-R382Q mutant was transfected along with the AGG-Luc reporter into 293T cells. After transfection for 24 h, the cells were treated with IL-6, LPS or IL-6 plus LPS for an additional 24 h prior to luciferase reporter analysis. Data are representative of three independent experiments, and bar graphs show the mean ± SD, with *P < 0.01. (**I**) The STAT3-R382Q mutant was cotransfected with or without CBP along with the indicated AGG-Luc variants into 293T cells. After transfection for 24 h, the cells were treated with or without IL-6 for an additional 24 h prior to luciferase reporter analysis. Data are representative of three independent experiments, and bar graphs show the mean ± SD, with *P < 0.01.

The central and C-terminal regions of STAT3 contain the DBD and the SH2 domain, respectively. STAT3 mutations of these two domains, including R382Q, R382W, F384S, R423Q, V463Δ, S465A, T662I, E652K, V637M, were reported to lead to HIES (14,17). All of these mutants lost SIE-dependent gene regulation, presumably due to their failure to bind to the SIE. To study the effect of the HIES mutants on AGG-dependent gene regulation, we constructed two sets of STAT3 HIES mutants: GFP-tagged STAT3 HIES mutants (R382Q, R382W or V463Δ) (Figure 5F) and Myc-tagged STAT3 HIES mutants (R382Q, E434E435A, V463 V464 V465A) (Figure 6A). The three GFP-STAT3 HIES mutants (R382Q, R382W or V463Δ) were transfected into 293T cells followed by IL-6 treatment for 30 min and subsequent confocal microscope visualization. All three HIES mutants showed nuclear translocation with a punctate distribution pattern in response to IL-6 treatment (Figure 5D).

Among the HIES mutations, R382 is the most commonly mutated residue. Surprisingly, while STAT3 binding to the SIE-element was disrupted, STAT3 with the R382Q mutation markedly enhanced its AGG-element binding activity in response to IL-6 treatment (Figure 6B). Purified STAT3 proteins, wild-type or mutant (R382Q or V461V462V463A) (Figure 6C), were incubated with AGG-oligo or SIE-oligo for DNA affinity binding assay followed by immunoblotting analysis. Whereas both R382Q and V461V462V463A mutation markedly disrupted STAT3 for the SIE-element binding, their AGG-element binding activity was actually enhanced (Figure 6D). Similar results were obtained by performing EMSA (Figure 6E).

We then compared STAT3-R382Q mutant SIE- and AGG-luciferase reporter activation. The R382Q mutation dramatically increased AGG-luciferase reporter activation (Figure 6F, upper panel) but reduced SIE-luciferase reporter activation (Figure 6F, lower panel). While the cytokines IL-6 and LIF activated STAT3 gene regulation in both SIE- and AGG-element-dependent manners, LPS failed to activate STAT3 gene regulation in an SIE-dependent manner (Figure 6F lower panel). The STAT3 R382Q mutant failed to activate the SIE-luciferase reporter regardless of CBP cotransfection. Surprisingly, STAT3 with the R382Q mutation significantly enhanced the transcriptional activity of the AGG-luciferase reporter in response to CBP cotransfection (Figure 6F).

Double Glu (E434, E435) and triple Val (V461-V463) were previously reported as the key STAT3 DBD motifs for SIE binding (5,6). As expected, STAT3 with a double Glu mutation (E434, 435A) or triple Val mutation (V461–463A) reduced the SIE-luciferase reporter transcriptional activity (Figure 6G, lower panel). In contrast, these STAT3 DBD mutants significantly enhanced AGG-luciferase reporter activity (Figure 6G, upper panel). LPS can induce IL-6 secretion. Treating the cells with LPS mimicked recombinant IL-6 treatment and enhanced AGG-luciferase reporter activation by the STAT3-R382Q mutant and CBP (Figure 6H). Consistently, LPS and IL-6 together enhanced both the wild-type and R382Q mutated STAT3 AGG-element dependent transcription activity (Figure 6H).

We also tested other HIES mutants, including R382W, R423Q and S465A. They all became more active in response to CBP cotransfection or IL-6 treatment in the activation of the AGG-luciferase reporter (Supplementary Figure S4). Two AGG-deletion mutations were introduced into the AGG-luciferase reporter vector (Supplementary Figure S1). Deletion of the AGG-element disrupted both STAT3 wild-type and STAT3-R382Q mutant activity in response to CBP cotransfection and IL-6 treatment (Figure 6I). These results demonstrate that the STAT3 HIES mutants are gain-of-function mutations in terms of AGG-element-dependent promoter activation.

### AGG-promoter activity is critical for TNF-α gene activation

**Figure 7.**
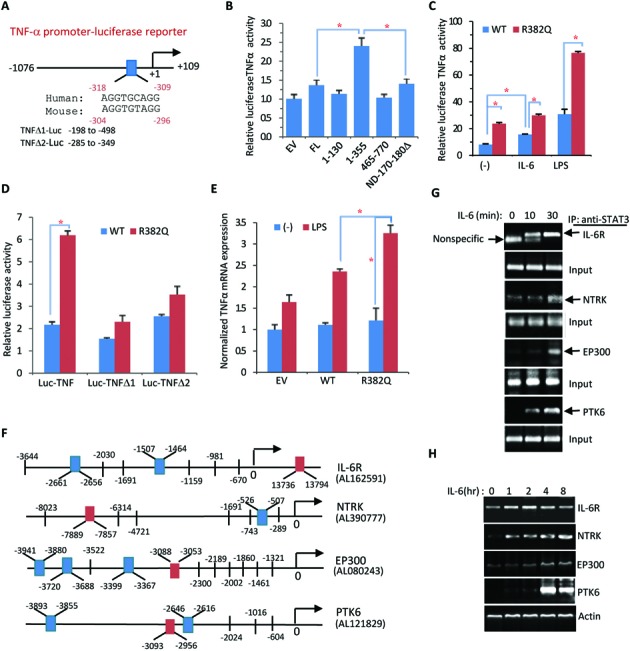
TNF-α promoter and other AGG-promoters are activated by STAT3. (**A**) Illustration of the TNF-α promoter luciferase reporter vector. As indicated, the conserved AGG motif ‘AGG(N)3AGG’ was identified from human and mouse TNFα promoters. (**B**) Myc-tagged STAT3-FL or indicated STAT3 domains were transfected along with TNF-Luc into 293T cells. The TNF-Luc activity was examined 48 h after transfection. Data are representative of three independent experiments and bar graphs show the mean ± SD, with *P < 0.01. (**C**) STAT3-WT and STAT3-R382Q mutants were compared for TNF-Luc activation in 293T cells. After transfection for 24 h, the cells were treated with IL-6 or LPS for an additional 24 h prior to luciferase reporter analysis. Data are representative of three independent experiments, and bar graphs show the mean ± SD, with *P < 0.01. (**D**) STAT3-WT or STAT3-R382Q mutant was transfected along with TNF-Luc or AGG motif deleted TNFΔ1-Luc or TNFΔ2-Luc into 293T cells for 48 h. Data are representative of three independent experiments, and bar graphs show the mean ± SD, with *P < 0.01. (**E**) STAT3-WT, STAT3-R382Q mutant or EV was transfected into 293T cells for 24 h followed by LPS treatment for an additional 24 h. TNF-α mRNA expression was detected by real-time PCR. Data are representative of three independent experiments, and bar graphs show the mean ± SD, with *P < 0.01. (**F**) Diagram of all AGG-elements identified within the promoter regions of *PTK6, IL6R, EP300* and *NTRK* genes. DNA sequences in the red box were used for ChIP-PCR analysis. (**G**) HepG2 cells treated with IL-6 for 0, 10 and 30 min. According to (F), the corresponding sequences were selected for ChIP-PCR analysis. (**H**) HepG2 cells were treated with IL-6 for the indicated times followed by RT-PCR analysis to determine the mRNA expression of *PTK6, IL6R, EP300* and *NTRK* genes in response to IL-6 treatment.

The TNF-α gene promoter, either human or mouse, does not contain any conserved SIE sequence, even though STAT3 activation of this gene in B cells was reported (20). An AGG-element was identified in the TNF-α promoter region between −318 and −309 (human) or −304 and −296 (mouse) (Figure 7A, Supplementary Figure S2). Figure 7A schematically depicts the promoter sequence upstream of the human TNF-α gene that matches the consensus AGG-element sequence. We cloned the human TNF-α promoter region from −1076 to +109 and constructed a TNF-α promoter luciferase reporter (Supplementary Figure S2). Different STAT3 domain truncations were then tested on this luciferase reporter. Interestingly, the N-terminal region (1–355) alone was much more active than full length STAT3 in activating the TNF-α promoter luciferase reporter (Figure 7B). Likewise, the constitutively active HIES mutant was much more active than wild-type STAT3 in activating the TNF-α luciferase reporter (Figure 7C).

The human and mouse TNFα promoters contain multiple (i.e., 2–3) NF-κB binding sites (27,28). LPS treatment markedly activated the TNF-α promoter, presumably via IL-6 induction (Figure 7C). However, deletion of these NF-κB sites had little effect on TNF-α induction by LPS in B cells, suggesting an indirect role for LPS in TNF-α promoter activation (29). Surprisingly, deletion of the AGG-element from the TNF-α promoter largely abolished the activity of the HIES mutant in activating the TNF-α promoter (Figure 7D). When TNF-α gene expression was analyzed in HepG2 cells, the HIES mutant markedly enhanced TNF-α mRNA induction in response to LPS treatment (Figure 7E). The *PTK6, IL6R, EP300* and *NTRK* promoters also contain AGG-element(s) (Figure 7F). IL-6 activated STAT3 was associated with the AGG-elements of these promoters, as revealed by STAT3-ChIP PCR analysis (Figure 7G). Expression of all these genes was induced by IL-6 (Figure 7H). Collectively, these data strongly indicate that the AGG-element is transcriptionally active and is responsible for the activation of TNF-α and other genes. Thus, the STAT3 HIES mutation is gain-of-function in gene regulation via the AGG-element and the N-terminal region 1–355 is necessary for optimal AGG-element binding activity and promoter activation.

## DISCUSSION

The STAT3 HIES mutation is a loss-of-function or dominant negative mutation in terms of SIE DNA binding and gene regulation in cells. However, in cells expressing the STAT3 HIES mutant, the TNF-α level was unexpectedly high (14,16), suggesting that STAT3 HIES is also a gain-of-function mutation in transcriptional activation. These STAT3 HIES mutants exhibit punctate nuclear distribution in response to IL-6 treatment, suggesting that they are associated with transcriptionally active chromatin (25).

The N-terminal region of STAT3 is sufficient for STAT3 nuclear translocation and punctate distribution, as this region alone is capable of manipulating gene expression. This helical region likely evolved from the ancient coiled-coil domain with some similarity to the helix-turn-helix. While the helix-turn-helix is the major DNA binding structure (30–32), the coiled-coil domain of the Uup protein has also been reported to bind DNA (33). Although the STAT3–AGG-element interaction is likely weaker than the STAT3–SIE-element interaction, our ChIP-cloning and ChIP-on-ChIP results revealed that only a small amount of the nucleic acid sequences pulled down by STAT3 contained an SIE. BLAST analysis of the AGG-sequence demonstrated that these sequences are abundant in the human genome and present in important gene promoters, including the AluS family left and right arms.

STAT3 exists in multiple isoforms in mammalian cells. The three major isoforms are STAT3α, STAT3β and STAT3γ. They have very different DNA binding activities and gene regulation activities (34–36). STAT3β expression can rescue the embryonic lethality of a STAT3α-null mutation by inducing the expression of STAT3α-insensitive genes (37). A promoter with a triple SIE has no response to STAT3β, but a promoter with a triple AP-1 element is fully responsive to STAT3β (36). STAT3β can activate important growth genes lacking SIE promoters. To activate canonical SIE promoters, STAT3 relies on the phosphorylation and acetylation of the C-terminal region (38–40). C-terminal post-translational modification events are exclusively required for STAT3 to undergo C-terminal dimerization and SIE binding (41). In contrast, STAT3 requires N-terminal post-translational modifications, especially acetylation, for AGG-promoter activation. Therefore, the STAT3 HIES mutants may rely on post-translational modifications by acetyltransferases, such as CBP/p300, to activate AGG-promoters in response to different ligands.

LPS induces gene expression either directly via NF-κB or IRF3 activation or indirectly via their gene expression products (i.e., IL-6) (42). Although LPS-activated NF-κB may or may not be responsible for the direct regulation of TNF-α promoter activation (29), IL-6 production in response to LPS treatment can activate STAT3, there by activating AGG-element promoters including TNF-α. These findings explain why the STAT3 inhibitor WP1066 significantly blocks leptin-induced TNF-α secretion from B cells despite the lack of an SIE-element within the TNF-α promoter (20). Although STAT3 activity in regulation of AGG promoter is weaker than that of SIE promoter, the AGG-element is far more widely distributed in promoteromics. STAT3 with the HIES mutation unexpectedly enhanced STAT3 intrinsic AGG-element binding and transcriptional activity, suggesting that patients with the HIES mutation may become more sensitive toward LPS in TNF-α induction and inflammatory responses. The presence of many AGG-elements in promoters and Alu sequences may explain why STAT3 is involved in regulating a variety of cell functions.

In summary, STAT3 is a dual specificity transcription factor that can regulate both the canonical SIE- and the non-canonical AGG-containing promoters. The HIES mutation can dramatically increase STAT3 intrinsic activity for AGG-element DNA binding and gene regulation activities.

## Supplementary Material

SUPPLEMENTARY DATA
