# Supplementary material for: The STAT3 HIES mutation is a gain-of-function mutation that activates genes via AGG-element carrying promoters
Source: Nucleic Acids Res. 2015 Oct 10;43(18):8898–912. doi: 10.1093/nar/gkv911 (PMC4605325; doi:10.1093/nar/gkv911)
Supplement: SUPPLEMENTARY DATA [file supp_43_18_8898__index.html]

The STAT3 HIES mutation is a gain-of-function mutation that activates genes via AGG-element carrying promoters — SUPPLEMENTARY DATA 

# The STAT3 HIES mutation is a gain-of-function mutation that activates genes via AGG-element carrying promoters

## SUPPLEMENTARY DATA

- SUPPLEMENTARY DATA
