# Supplementary material for: The STAT3 HIES mutation is a gain-of-function mutation that activates genes via AGG-element carrying promoters
Source: Nucleic Acids Res. 2015 Oct 10;43(18):8898–912. doi: 10.1093/nar/gkv911 (PMC4605325; doi:10.1093/nar/gkv911)
Supplement: SUPPLEMENTARY DATA [file supp_gkv911_nar-00708-m-2015-File003.pdf]

SUPPLEMENTALDATA

Figure S1. Sequence used for SIE-Luc, S6-SIE-Luc, and AGG-Luc constructs

Figure S2. Human TNF-α promoter sequence used for luciferase reporter (TNF-Luc) construction

(**A**) Human TNF-α promoter sequence analysis.

(**B**) Alignment between human and mouse TNF-α promoter.

Figure S3. The AGG elements of a human Alu consensus sequence

Figure S4. STAT3 mutants on AGG-Luc and SIE-Luc luciferase reporter activity

The regulation of STAT3 mutants (R382Q, R382W, R423Q, V463del, S465A) on AGG-Luc and SIE-Luc reporter was test, experiment method is same as Figure 6D.

Appendix 1. promoter of STAT3 binding sequence

Appendix 2. The sequence from chip-clone results

HindIII-sequence (5’-3’)-SalI

Luc-SIE	ACAACAGCCTGATTTCCCCGAAATGACG
Luc-STAT6	AGCTTCTTCATCTGAAAAGAGCTTCTTCATCTGAAAAG
Luc-AGG	TTAGTCACTACTCTGCACTGAAAGGCTGTGCGCGGAGGGCGAGGGAGGGGCC
Luc-AGG- Δ 1	TTAGTCACTACTCTGCACTGAAAGGCTGTGCGC-----GCGAGGGAGGGGCC
Luc-AGG- Δ 2	TTAGTCACTACTCTGCACTGAAAGGCTGTGCGCGGAGGGCG------GGGCC

Figure S1. SIE-Luc, STAT6-SIE-Luc, and AGG-Luc constructs

A Human TNF-α promoter

```
(-1076)CCAGGGCTATGGAAGTCGAGTATGGGGACCCCCCCTTAACGAAGACAGGGCCATGTAGAGGGCCCCAGGGAGTGAAAGAGCCTCCAGGACCTCCAGG
TATGGAATACAGGGGACGTTTAAGAAGATATGGCCACACACTGGGGCCCTGAGAAGTGAGAGCTTCATGAAAAAAATCAGGGACCCCAGAGTTCCTTGGAAGCC
AAGACTGAAACCAGCATTATGAGTCTCCGGGTCAGAATGAAAGAAGAAGGCCTGCCCCAGTGGGGTCTGTGAATTCCCGGGGGTGATTTCACTCCCCGGGGCTG
TCCCAGGCTTGTCCCTGCTACCCCCACCCAGCCTTTCCTGAGGCCTCAAGCCTGCCACCAAGCCCCCAGCTCCTTCTCCCCGCAGGGACCCAAACACAGGCCTC
AGGACTCAACACAGCTTTTCCCTCCAACCCCGTTTTCTCTCCCTCAAGGACTCAGCTTTCTGAAGCCCCTCCCAGTTCTAGTTCTATCTTTTTCCTGCATCCTG
TCTGGAAGTTAGAAGGAAACAGACCACAGACCTGGTCCCCAAAAGAAATGGAGGCAATAGGTTTTGAGGGGCATGGGGACGGGGTTCAGCCTCCAGGGTCCTAC
ACACAAATCAGTCAGTGGCCCAGAAGACCCCCCTCGGAATCGGAGCAGGGAGGATGGGGAGTGTGAGGGGTATCCTTGATGCTTGTGTGTCCCCAACTTTCCAA
ATCCCCGCCCCCGCGATGGAGAAGAAACCGAGACAGAAGGTGCAGGGCCCACTACCGCTTCCTCCAGATGAGCTCATGGGTTTCTCCACCAAGGAAGTTTTCCG
CTGGTTGAATGATTCTTTCCCCGCCCTCCTCTCGCCCCAGGGACATATAAAGGCAGTTGTTGGCACACCCAGCCAGCAGACGCTCCCTCAGCAAGGACAGCAGA
GGACCAGCTAAGAGGGAGAGAAGCAACTACAGACCCCCCCTGAAAACAACCCTCAGACGCCACATCCCCTGACAAGCTGCCAGGCAGGTTCTCTTCCTCTCACA
TACTGACCCACGGCTCCACCCTCTCTCCCCTGGAAAGGACACCATGAGCACTGAAAGCATGATCCGGGACGTGGAGCTGGCCGAGGAGGCGCTCCCCAAGAAGA
                                              +1
CAGGGGGGCCCCAGGGCTCCAGGCGGTGCTTGTTCCTCAGCCTCTTCTC(+109)
```

The deleted sequence in Luc-TNF-Δ1： from -198 to -498 （marked with underlined）
The deleted sequence in Luc-TNF-Δ2： from -285 to -349 （marked with Italic shadow）

B lignment between human and mouse TNF-α promoter

```
Human :  TCCTTGATGCTTGTGTGTCCCCAACTTTCCAAATCCCC-GCCCCCGCGATGGAGAAGAAACCGAGACAGAAGGTGCAGGGCCCACTACCGCTT
Mouse:   TCCTTGATGCCTGGGTGTCCCCAACTTTCCAAACCCTCTGCCCCCGCGATGGAGAAGAAACCGAGACAG-AGGTGTAGGG-CCACTACCGCTT

Human :  CCTCCAGATGAGCTCATGGGTTTCTCCACCAAGGAAGTTTTCCGCTGGTTGAATGATT-CTTT-CCCCGCCCTCCTCTCGCCCCAGGGACATA
mouse:   CCTCCACATGAGATCATGGTTTTCTCCACCAAGGAAGTTTTCCGAGGGTTGAATGAGAGCTTTTCCCCGCCCTC-T-TC-CCCAAGGG-C-TA

Human :  TAAAGGCAGTTGTTGGCACACCCAGCCAGCAGACGCTCCCTCAGCAAGGACAGCAGAGGACCAGCTAAGAGGGAGAGAAGCAACTACAGA
Mouse:   TAAAGGCGGCCGTCTGCACAGCCAGCCAGCAGAAGCTCCCTCAGCGAGGACAGCAAGGGACTAGCCAGGAGGGAGAACAGAAACTCCAGA
```

Figure S2. Human TNF-α promoter sequence (GeneBank: KF887971.1) used for luciferase reporter (Luc-TNF) construction.

```
GGCTGGGCGTGGTGGCTCACACCTGTAATCCCAGCACTTTGGAGGCCGAGGTGGGTGGATCACCTGAGGTCAGGAGTTCAAGACCAGCCT
GGCCAACATGGTGAAACCCCGTCTCTACTAAAAATACAAAAATTAGCCGGGCGTGGTGGCGCGCGCCTGTAATCCCAGCTACTCGGGAGG
CTGAGGCTGGAGATCGCTTGAACCCAGGAGGTGGAGGTTGCAGTGAGCCGAGATCGCGCCACTGCACTCCAGCCTGGGCAACAGAGCGAG
ACTCCATCTC
```

Figure S3. The “AGG” elements of an human Alu consensus sequence

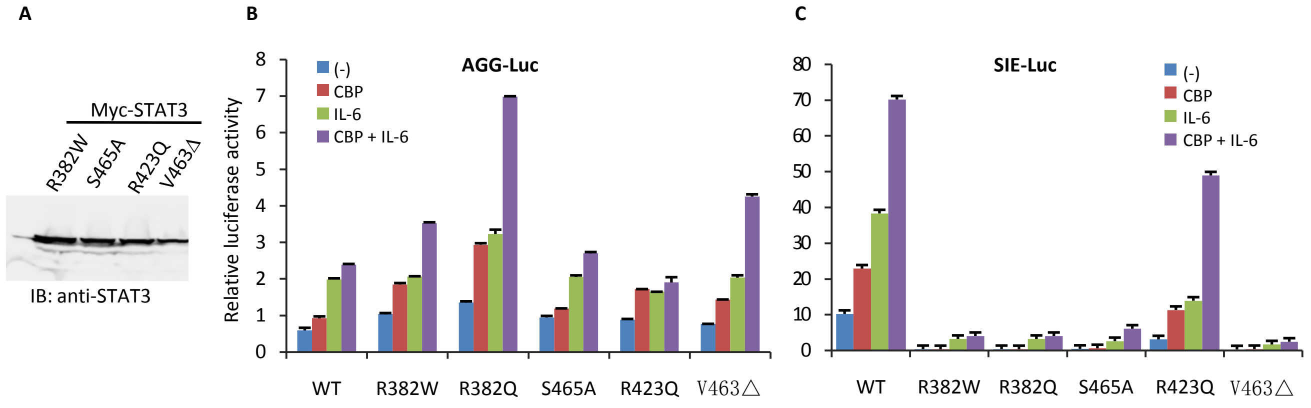

Figure S4. STAT3 mutants on Luc-AGG and Luc-SIE luciferase reporter activity analysis.

Appendix 1

promoter of STAT3 binding sequence

symbl	ref_NM	TAG_SEQ
Ptma	NM_008972	CTCCCGCTCCCCCTCCTCCCTCCCTCCCCCTCCTTCTTCCGAGCAGTCGCGAGGGAGGGGGAG
Mrpl30	NM_027098	TCCTGGGCACCGCCAGCAAATTCCAAGAGCTGACCCACTGCGCTTCCAGGAAGCGCACGCGTGAC
Mitd1	NM_026913	TCCTGGGCACCGCCAGCAAATTCCAAGAGCTGACCCACTGCGCTTCCAGGAAGCGCACGCGTGAC
BC031781	NM_145943	GCTCGCCCTTTATAGCCAGTCCCTTGAGCCCCGCCCTAGCAACCGCCCCTAGCAACGCCTAGCTCCAGCG
Eya1	NM_010164	GGAAGGGCCGGAAGGGCCTCGAACTTTCCAGTAAAAGATGTCTTCTCCTCCTCCTCCTGCTTACCCCGTAGATTAAGCACCC
Tmbim1	NM_027154	TCAGCAGCAGCGCATAAAAGCCAAGTGGAGTAGCACAGGCCTAGAGTCCCACGCTTGTTATGGAGATGTCATGCCAGGAAACGTTCAAGAACTTCAAAAA
Ddx59	NM_026500	GGGAATGCGCGGGATGCGGGCGTTGCCTGGAGCCGGAGGAGGAGGTTGTCGGCTCTGCCGC
Ddx59	NM_178052	GGGAATGCGCGGGATGCGGGCGTTGCCTGGAGCCGGAGGAGGAGGTTGTCGGCTCTGCCGC
4930523C07Rik	NM_001024470	TAGCAGAGCTGGGTGCGGTGAAACCTCAGCCAGGGACGCAGGGGGCGCTTTCCTCCACATCACACTGGTGGCACCCAC
Psen2	NM_011183	TTCCAAAACCCCTGGAAAGTCCCAGTCTTCCTCCACACTAGGGAAGGGTTTCCCCCCCAAACAGGGTACGCCCTGCGACATCTCACACA
Lefty2	NM_177099	TCTGCCCACTGTAAAAGTTTCCTAATCCACATCTGCTTCCTTCTTTGGGCCGTGGCTAATTGCCCAATCACTGTATTTCCCCA
4930511H11Rik	NM_030025	AACTACGTTTCCCAGCGTGCTCTGCTGGAAACACAATGGAGTTTGGCGTCATCCGGGTCACGTGGTCTCGCGTTT
Ctdsp1	NM_153088	CTTCAAGGGCAAGCGGGACACAGGTGAGAGGAGGGTGCTTTCTCCCAGGGCCGCAGAAGCCACCTCCG
Paqr8	NM_028829	CTGGGCAGCCCTCCCCTGGGCCGGGAAAGGGCGGAGCTGGGCGTCGCCATCCGGGTTGTCCCCTCCTCCCGGCCGCTCTC
Slc41a1	NM_173865	CTGAGTTCCCGTCACTCTGCTGCGTTTTCCCCCTCACCCCGCCCCCAGCTCAAGAAACGCCGGGGAAATGGGCGGGGCCTGGGCGGG
Fn1	NM_010233	CACGCGCGCAAAAACTTTAGCCACAACTTGAGCGGGCGCTTGAGGACCAACCCATCCCTGCCGC
Cul3	NM_016716	GTGGGCGGGGGAATTTCCGGAAGGGGGAGAGGGGAGTGTTGTGTTGTGGGCTGGCGGGGCT
Uck2	NM_030724	GACATTGCAGTTGACAGTCCTTTGTCTGCCTGCCCCCTCTCCTGAAGCACCAACACCCCTCCTTCCTAGC
Tomm40l	NM_001037170	TTTTTAAAGGGTGGGGGGCCCATCCCCCGTATGCCAGGAAAGCGAGCTCAGCTGCTCAGCAGGTA

Rrs1	NM_021511	CCCCGCGAGAAGCCGCTGCCCCGACCACGGCCGCTCACCCGCTGGCAGCAGTTCGCGCGCCTT
Adhfe1	NM_175236	CCCCGCGAGAAGCCGCTGCCCCGACCACGGCCGCTCACCCGCTGGCAGCAGTTCGCGCGCCTT
Slc9a2	NM_001033289	CCCAGCAGGGAGGAAAAGGGTTAGGCTCTGCCTGTTTCAGTAAATCTTTACAAGGAAACAGAATCTTCACAGGCAGTTCCACTTCAAAGCTGGAAAACT
Hrb	NM_010472	GAGGAAGATAGCCTGCTTACACGAAGCTCTTTGCACGTACAGGCACCCAGCGGATAAGGGCTGTAATTTATTGAC
Fbxo36	NM_025386	GGGCTCCTCCGCTGGGCCAGCCCCGCTCCCTATTGACAGCGCATCCTGTCGCCCCTCCCTCCGCAACGTTCAACTCGA
Trip12	NM_133975	GGGCTCCTCCGCTGGGCCAGCCCCGCTCCCTATTGACAGCGCATCCTGTCGCCCCTCCCTCCGCAACGTTCAACTCGA
Mybph	NM_016749	GTGGGGTATGAGGACAAGGAGAGTGTCAAAAACAAGGTAGGTTTCTCAGAAGCCACACCCCGGAACAAAGAAGGAGAGGC
2310003C23Rik	NM_029607	CGTTCCTATCCGGCAGTGTTAAAGAGCCTCCAACTTCCCCCATCCCTTACCGGAATATCTAACCCAT
Sulf2	NM_028072	CTCAGTCTCCCAGTTGGGACTGAGCGCTGGAGGAGGGTGGTATCATGTGATTTCTCTTATGT
Mybl2	NM_008652	ACTCCCAGGTTTGACTGCCCCGGGATTCTAGGAATGCTCTCCCCAGGGGTTATGTGTTGACTTGCTGATA
Cstad	NM_030137	CTCCGCCCAGCTGGGGGTGGTGTCTTCCGGGAAGTTGAGGGGTCGCACCTGGGAGGGAGGGGGAACTATCCGGTGC
Rpap1	NM_177294	GTCTTCTGCCCTTCATCCCACCACCCACCCACCCCCACTAGAGTGTTGGGAGCTACCACA
Prei4	NM_001042671	TGGCCCGACAGCCTTAGCCCATCTGGCTGGGGTCAGCTGAGGTACTGAAGGTCAAGGTGG
Prei4	NM_001042672	TGGCCCGACAGCCTTAGCCCATCTGGCTGGGGTCAGCTGAGGTACTGAAGGTCAAGGTGG
Prei4	NM_028802	TGGCCCGACAGCCTTAGCCCATCTGGCTGGGGTCAGCTGAGGTACTGAAGGTCAAGGTGG
Ndufa8	NM_026703	AAGCGTAAGCCATTCCAGGAAGTCTAGCGGTCCCGAGGGGCGGGGCTTCATCTGGTCTCCG
1700010A17Rik	NM_029309	AAGCGTAAGCCATTCCAGGAAGTCTAGCGGTCCCGAGGGGCGGGGCTTCATCTGGTCTCCG
Wfdc2	NM_026323	TGCCTTCCCCTCCCCCTCACACTAATCCACATTCCGGTAAGAGAGGGGCCAGTCAGCCAGGGAT
Mdk	NM_001012335	CCTGCAGGAGCGGGAAAATCAGCTCCGCCCCAGGCGCTGTGAGCCTAAGGCCCTAGGCAGAGGT
Mdk	NM_001012336	CCTGCAGGAGCGGGAAAATCAGCTCCGCCCCAGGCGCTGTGAGCCTAAGGCCCTAGGCAGAGGT
Mdk	NM_010784	CCTGCAGGAGCGGGAAAATCAGCTCCGCCCCAGGCGCTGTGAGCCTAAGGCCCTAGGCAGAGGT
Ada	NM_007398	CCCCCCCCCCGCCTGCAACTCTGGCGCGCATTTCCCGGAATTCTGAACCCCAGACCTTCCCTGCTGGCTACACCCTAGGAAGCTCC

3300002I08Rik	NM_027017	GGCACACGGAACATGCCAAGCAGAAATACTCAGGATTGCCCTGCTTCACAGAAAGGGCCATACAGAAACC
Cpne1	NM_170588	CAACACCTCCACAGCCGCTTCACAAAATGGCCGCCCGCCCGCTCCGGCGCTCCGCGTTCCCGGAGTAGGCGGGGCGCCGCTGCCGACGTCA
Cpne1	NM_170590	CAACACCTCCACAGCCGCTTCACAAAATGGCCGCCCGCCCGCTCCGGCGCTCCGCGTTCCCGGAGTAGGCGGGGCGCCGCTGCCGACGTCA
Rbm12	NM_170598	CAACACCTCCACAGCCGCTTCACAAAATGGCCGCCCGCCCGCTCCGGCGCTCCGCGTTCCCGGAGTAGGCGGGGCGCCGCTGCCGACGTCA
Rbm12	NM_029397	CAACACCTCCACAGCCGCTTCACAAAATGGCCGCCCGCCCGCTCCGGCGCTCCGCGTTCCCGGAGTAGGCGGGGCGCCGCTGCCGACGTCA
Rif1	NM_175238	AAATCTCAATGGAAGGAATGTGGGTGGTGGTGGGGTAAAGGACGGAAAGATTCCACGGATTTCCCCACT
Cstf3	NM_145529	TGTTTCAGGGGGTAGTTCTGATCACCTGGCAACCGCACCTTTGTTTCAGGGGTTAGTTCTGATCACCTGGCAACCGCACCTTTGTTTCAGA
Cstf3	NM_177253	TGTTTCAGGGGGTAGTTCTGATCACCTGGCAACCGCACCTTTGTTTCAGGGGTTAGTTCTGATCACCTGGCAACCGCACCTTTGTTTCAGA
Cstf3	NM_001037326	TGTTTCAGGGGGTAGTTCTGATCACCTGGCAACCGCACCTTTGTTTCAGGGGTTAGTTCTGATCACCTGGCAACCGCACCTTTGTTTCAGA
Asxl1	NM_001039939	GAATCAAAAAAAAGCTTTGAACGGGTTAACCGCGCGCGCGGCCAGGGGGGAGTCCAGCCACCGCCCCTTCCCAGCACCACCTCCCAGAAACCCTCAGGCCTCC GCTAGTTCCCGAACCTCGCGAGACCCCGCCCCA
Endog	NM_007931	AACCAAAACCAGAAAAATACAGTTTGCAGTAACAAGTGCCAACCAGAAGGTGGCGCTCTCACACATGACAACTCA
Adal	NM_029475	CGGCGTGCGCCGCACGGGTCCCGGGACCAGCAGCGCCGCAAAGGGGTCGCGCACGTATCCGTGTGCGG
Slc27a2	NM_011978	GCGGTGCGTTTATAAGGTACTTGATTAAGCAAAGGCACTGCGCTGTGTGATAAGAATAACTAACC
Rab14	NM_026697	CGCAGCCAGCGGAGCCCGGAAGAGGGTGGTGGAGACTAACCTGACGGCCACGCCCCTTCGTCAGTTCGCCCCTCCGGAGTAGACCCGCCTCGGCCTCGGCCCG CCCCCACTG
Rbm18	NM_026434	GCTTCGGGTACTCACCTGCTAGCCTCAGAGCTGTGCCCTCAGCTCATGCCCCGAAAGCAGCTTCCGACGCTGGGGT
Mrrf	NM_026422	GCTTCGGGTACTCACCTGCTAGCCTCAGAGCTGTGCCCTCAGCTCATGCCCCGAAAGCAGCTTCCGACGCTGGGGT
Orc4l	NM_011958	CAGCGCCTTGGAGCCTTCTGGGAAAACCATCTCATTTCCTCCCCTCCCCCTCCTCCTTGCCTTCCACCAACCAGCCTCCCGTCAGAACGGGACATGCGCAGTGAG TGCCTCCCGTCTCGTCTCCCCGAG
Rif1	NM_175238	ACCTGTGTATCCCAGGGCCACCGTCACCCCTCTACACCTGGGGTCCAATGGAAGTAAACCAATTC

Pak6	NM_001033254	CCTCTCATTTCCTCTGTGTTTTTTGCTGAGTCACCTTATCTCCCAGAGCTGCAGTTGGCTGTTGGCATCTTTTGGAGGAGGGGTGGGGCTTCCTGGAATTC
LOC433479	NM_001013802	ATTTTAGTTACAAATTAACATTTAGACCTATTGGCTATAAAGGGCTAAGGGGCCACAGCAGT
Nxt1	NM_019761	CAACATCCAATCAGAGCTCCCTCAACCGGATCTTCTAGGATAGTGCCTAACCCGGAACCTACACTGTCTTTGCCGCCACA
Stx16	NM_172675	CCGCTCCCGGAACGTTGCAAGCAATCTCATGAATGTCGCAGGGGGGCACCCGCGAGGAACGG
Tmem79	NM_024246	CATTGACTGACGTCCTCCAAACCAGGTTCATGTCCTTCATTTGCCCCTCTCGCTATGTGACCTT
Tpd52	NM_001025263	GCTGAGCACTGGGAAGCGGGCAGGGGAGGAGGCCATGCACATTCACAAACAAGCATCAGGAGTTG
Tpd52	NM_001025264	GCTGAGCACTGGGAAGCGGGCAGGGGAGGAGGCCATGCACATTCACAAACAAGCATCAGGAGTTG
Tpd52	NM_009412	GCTGAGCACTGGGAAGCGGGCAGGGGAGGAGGCCATGCACATTCACAAACAAGCATCAGGAGTTG
Sox2	NM_011443	ATGCCAGGAAGTTGCGTGACCCTCCCCCCATCCCCTGCACTCGGGAGCTTCTTTCCGTTGATG
Ccna2	NM_009828	TGATCTGGGATTAAAGGCGTGTCTCTATGCCCCGCATTCCAGTAAACTACCCCACCCCCACCCCCAATTAAAGATGGGTTA
Mcl1	NM_008562	GCCCCTTTCCCCTTTTACGGGAAGTCCTCGCCTGCGTCAGCACGGCCCTAAGGCGGCGGCAGGGA
2700083E18Rik	NM_001029890	CCTCTGAGATCCCGGAACTGGGGGGCTGGGGGAGGGGGAAGGGCTTGCTACCTCCGAGAAGAGAGGGAACCACATCCTCTACCGGGGGTCCCACCCCCAACC GAACGCCCCTTCCTGGCCCC
Sox2	NM_011443	AACAGAGCTTTCCCCCAATACTGGTGGTCGTCAAACTCTGCTAATTAGCAATGCTGAGAAATTCCAGTTAACAAGGGCATTCTCC
Wdr77	NM_027432	GCAAGATGGCCGCTCGCACATCTCGCGAGAAGAGATCAGCTCGCTTTTTCTCGGAAGTTCTC
Dnttip2	NM_153806	CTAGTCCGATGGCGGAGACCGCTGGCCAGAACTGCAGTGGAGCCGGGGAAGGAACGATTCTCC
2310010I16Rik	NM_025519	CGAGCCCGGCCAGGAAATGCCCAAGAGTGGGTGTCACCTGCCGAAACAACTTGTTGATTCCCAGGAGCTCTGTCGGCTCCGACTGTGGCTTACCTGTCTC
Tiparp	NM_178892	GACACTCCCCTCCCTCCTCCTCCTCCTCCCCGGAGCGCCGAGCCGCCGCGCGCCCCTCCGTCC
Scamp3	NM_011886	CTGGGCTGCGGAGACTGGCTTAGGGATATCACGTGCGGTACTTCCCGCTTTTGGGTTTTTTTTTCCT
Dennd4b	NM_201407	ACTTCCCGTCATTCGGCATGTGGGCGTTGGGACCCAAAGTGGCTCCAGTGCCGTTGTTTTCCCCCAAGGGAGAA
Adh7	NM_009626	CGTTCTGCTGTTTTTCCCTACTTAGGCTTTCATCTTACATCCTTTGTGCTAGCCCTTGTTCCCAGCCTCTCCCTGTTCCGAAAAGCATCACCTTCCATC
Cyp7b1	NM_007825	ATGCTTAATGCAAACCTGAATAGCTACAGCCTGCGTGGTGCAACTCCATTCGGGCCTCTCAACTGTAGCCCGTGTGTGAGTGTGTGGAGGGTGAAAGAAGTCTTT ATTAGGACATCCACTGGCT

Pcdh18	NM_130448	AAACCCTATAGACAAACTCAGAAAACTACCATAAGCAACTGGGACTTTAGAAACTCACAGCCATCTGGCCTATG
Kpna4	NM_008467	TCGTTCTTTTCTCCCCCGGTGTTACTTGAGCGCTTCATTCACTTTGGAAGGCAAAGAGGT
Hcn3	NM_008227	GCGCACCCACCGCTTCTCTAACTCTGGAAGGCGCAGGGGAAGCGGCTCACTGGCCCGAAGGGCC
Clk2	NM_007712	GCGCACCCACCGCTTCTCTAACTCTGGAAGGCGCAGGGGAAGCGGCTCACTGGCCCGAAGGGCC
Csf1	NM_007778	GCATTGTTCAAAGAATCTACTATACTACTATACTATCCTCTCCCCTGGCTGTAGGGAGAAGTGGGGAGCATCCAGCTACCTGGGTAGGAGAGGAAGGACTTGGTC C
Ddah1	NM_026993	GGATTATTTAAACTTTGATATGTGAAAGAGGAAAAAGAACAGGCTTTGTATCACCATCAAAGAAAG
Rpl22	NM_009079	TTCGCAGTGGGACAGCCCGTCTGACTCCTCCCTTTCCCGACAGCCGACTTCGCCCTGGGA
2410081M15Rik	NM_028603	GCTAGGGCTCTGGAGACTTCCCAGAACTGTTGGGGGAGCCTGGAAACTGTTGCTGGGGAGCCTGGA
Fabp3	NM_010174	GTGGGACTGCGGAGAAAGGCGCAGGCGGGAGACATTCCGCAGGGAGGGGCTAGCACGTGT
Spsb1	NM_029035	GCTCCTCACAGCCCTCCCCTCCGCACTTTCCCGCTTAGCCCCGCCCTACACCACGCCCCTTCTC
Sdhb	NM_023374	CCCTTTAATGAGGCTACCTGGCAGAGAAGCCAGATTCAGGGTGAGTTCCCAGAAACTTGGCCC
Scp2	NM_011327	GCGTCGGGGGCGGGGCCGCAGCGTCAGGGGCGGGGCCGAGTTACGGGAACCGACCGGAGGAGGCAGG
D630039A03Rik	NM_178727	TGGCTGTCCTGGAAAGGTCAGCCTCAGTGGGTGGAGGAGGGTCACAGGCTTGAAGAGGAAGCC
Lin28	NM_145833	ACTGCTCTGGTCTCTGACACCTCTGGGGTGTCCCTTCCCAGAACCTTGAGATTTGACACCCCCT
Hes5	NM_010419	CCCCACCCCGCAGTGCTTCTGCCGGGCAAAGCCCGCCAGCCAGAGGCAGCCTTTACCCCTCCCCCTT
Orc3l	NM_015824	GAGGCATGGCGTGTGGCTTTCGTAGGTCGATCGCCTGTCAGGTACAGTCCAGAGCGGCGAGCCAGCGGGAGCCAGGGCC
Rarsl	NM_181406	GAGGCATGGCGTGTGGCTTTCGTAGGTCGATCGCCTGTCAGGTACAGTCCAGAGCGGCGAGCCAGCGGGAGCCAGGGCC
Nipsnap3a	NM_025623	GGGGTTCGTGGTGCGCGTGATGACGACGCTGCGAGGTGGGCGGGTCTGCAGTGCGGAGAACGGGCCGTATTCTGGGAACTGGGCGGAGC
Cpne3	NM_027769	TATCGCCGCAGAAGTCGAGTGAGGGTGGAGAGCCAGCGAGGAGCACCGCAAGGCGGCAGGACCGCAGGGGCTTTAC
Dnajc6	NM_198412	GAGTTACAACCCAGTCCACCAGGTCGATCAACCCAGGCGTCTTTCACTTCCAACAAAGACCAGCT
Prdx1	NM_011034	AGTGCTGGAGCTCTACTCGCATTCTTCACGTGGCGCTTAGTTCCCGGACCTGTTGGAACCCTGAGGCTAAC

Psmb2	NM_011970	GCTGTCTCACTGGTGAGGTCGTGTACCCGGGAAGTGTCCCTGTCCGTGGCCTCTGCAGCCTCCCGCTTCCCGGCACCATGGAGTAC
C79267	NM_183148	CCCAGTGGTCAGCCGAGATTTCTCATCTGTCACGTGGTCCTAGCGCTGGCTCCTCTGGAATGCTGAGAGGCCACATCTGGCTGTGACGAGGCTG
Aco1	NM_007386	GGAGGGCGCACCCGGACTAGACTGCAAGCTTCAGCCATCGCTTGCAGCTTCTAGAGGGAGGA
Tal1	NM_011527	TTCCTGGAAACTCGCTTTGGGCGCGGCAGACCGCGCAGGACCTCACGGCAAGCTAAGTAACTGCTGGTCTCTCAGCG
Hnrpr	NM_028871	CTCTCTCTTATTGGCCCGTCAAGCTGCCCGTCATACGGTCTCGTGGGCCGATTGGTCGGCCGCACGGAGTCCCTGGAAG
Ankrd6	NM_001012450	GCAGAGATGTCCTAAGCAGTGGGTGAGGGGCTTTCCTGCAGGGCTGGTGTCCGGGCGTGAATAGCATTTGGTAACCC
Ankrd6	NM_080471	GCAGAGATGTCCTAAGCAGTGGGTGAGGGGCTTTCCTGCAGGGCTGGTGTCCGGGCGTGAATAGCATTTGGTAACCC
2610528J11Rik	NM_025572	GTGAGTCCTCCCTGCTGAGGACGCTGCAAATCCTGGCTGGTGCCCCGCCTTCCCTTCACCTGCACTTCCGCCCCGCCCACCT
Smap1l	NM_133716	CAGACAGATCCCTGGAAGGCAAGGTTCTGGTTGTGCTCAGCCATTAGTCAACAATGGGCACAGGCAGGAAAACAAAGCAATGCTGAGTTACTTGGTTTCTCTGC TTGTTTTTCCTGCTGCCAGAGGCATTTCCTTCCTGTGCTGACA
Tcea3	NM_011542	GCTATAGCCAAGGACTTTTCAGCTCCTCCCCCGGCCACCTCCTCACCTCCCCACCCCCTGCTGACATGTCAGTGATCCCCAAGCTTTGT
Agtrap	NM_009642	TAACAGGAAGTCTCCCCTGCTGAGCCTTCAGACATGCTGAGAGCTCTCAGGAGGCGGGAGCTGCCCAGCCTGGGCTGGTGGGTGTGACT
Epha7	NM_010141	CAGTAGCTAAAAGCCAAGTGCTCCCGACTGCACGCCGCTGGCCTAACGCGGCTCGTCCCCGCTTTCCCGGCAACCGATCTGGGAGGGAATCCTGGAC
Topors	NM_134097	AGAACCCATTCCTAAATTGAGCGTTCACGCCCTGCCCTCGGCTCCGCCTCTTCCTGGAGGACACGCTTTTCCGCCTCTGACGTC
LOC622404	NM_001037913	AACAGTGACTTGCGGGGGAAGTCTACGCGCGTGTGCACGCGGCACTCTCTGCCCGTGGTCCGGGGACTTTCCCCTAGGCGAAAGGATAAGGAACATGTC
Akap2	NM_001035532	TTGGCTGGTTTAAACAAGACATCATTTAATGATGTCAAGCCCAGACTCCTGCGACCCAGATTCCTTAGCCCTTTCCCCTTAACCCGCTGGAGTTTCTGGTGGCTTT GATTTTA
Akap2	NM_001035533	TTGGCTGGTTTAAACAAGACATCATTTAATGATGTCAAGCCCAGACTCCTGCGACCCAGATTCCTTAGCCCTTTCCCCTTAACCCGCTGGAGTTTCTGGTGGCTTT GATTTTA
Akap2	NM_009649	TTGGCTGGTTTAAACAAGACATCATTTAATGATGTCAAGCCCAGACTCCTGCGACCCAGATTCCTTAGCCCTTTCCCCTTAACCCGCTGGAGTTTCTGGTGGCTTT GATTTTA

St3gal3	NM_009176	CACAGCATCCCAGTGTCGGCCCGTCAGCTGAGGACGGACAGCTGCTGCCAAGCACCCACAATAGGCTTGTCTTTTCCCGGCAGAATGCATCGAGGGTGGAGTG AGAAGGTGAAAGGTCGGACACGTCATTCCTTTTCTGTGTACAGCTT
Zmpste24	NM_172700	CACCTGAAATATCCCTCCGGATTCTGTGTGCAGGGATCCAATTCAAAGGTTCCGGCCTTCTTCCTGGGAACGTCAGTGTGGTCGCCGCCTCTCCTTTGCTTTGGC GGGTTACCAGAGAGGGCAATGGACCCCGGTGCTTAA
Gjb3	NM_008126	AGGAGGCATCTCTGTTCCAATATCCCCTTAGGGTCTGTTCTCAAATTACTTCGAGTCTGAACCCGCCC
Phactr4	NM_175306	ATCCTACCTGGTCTCTGGCCGCCTGGCTCCAGCCAGAGCCTTCGGCCCCAGGGGAGGGGG
Necap2	NM_025383	CACTCTCCTCCATCTCTATCGCCCGAGCCCTCGGGACTTCCGGCTTCTCCCCGCCCCTTCAGGACCGGAAGTGATGCGACTCTATGACCAAGTTCTGTCTAGAGC GGAAGCT
Tmem142a	NM_175423	CCACAGGAGCAGCTGCAAAGAGAAGCGAGGCCTGACAGCGTGGGGCGCTTCCAGGAAGGCGAGC
Wsb2	NM_021539	CTCCTCTCCCCTTAGTTCCTCCCCCTTGGCTTTCCCAGCATCCCTCCCGCTGGGGCCGTTG
Noc4l	NM_153570	TCCCGGTTCCTGGAAACCGAAGTGTGCGCCGAGCGGAGTCGCGCCCGTGACGTCACCCGAGCC
Ddx51	NM_027156	TCCCGGTTCCTGGAAACCGAAGTGTGCGCCGAGCGGAGTCGCGCCCGTGACGTCACCCGAGCC
Ephb4	NM_010144	GGCTACTGCCCCAACATGCACAATCAGGGGTAGTAGGCATGGGACACCCAGCACCCCTGGACAGTCCCTAAGT
Fastk	NM_023229	AGGCGCTGCGAGCAGAGGAGCCGCGAGCCCCGCCCTCCGCTTCCGGGAAATGCGCACGTCGCGGGGGCGGGG
Crot	NM_023733	CCTAGGCTGCAGACCCCGGGAACGCAGCAGAAAGTCAGATAAGCGCCCTGCAGTCCCCGCTACA
Tnip2	NM_139064	CGTCCTGTCTACCAGACCTTGGGTCCCCAGACGACATGGCTGTAGGCCTTCCGTGGGGACTTGGGCGTCTCCAACTTCTCCACACAGGGGGAGCGAAAACCAG CCTT
Msx1	NM_010835	CAGCCTCTCTTGGGCCCTTTCAAAAACCAAACTGCACTGAAGTACTAATGGTGCCGCTGCCA
Apbb2	NM_009686	GGAAACGCTTCCTTGCCTGCTCCCGCCTCTGCGTCCCAGCCCGGTCGCCTGCTTCCTAGGCTCATTCCCTTGGCGGCCTGC
D430033A06Rik	NM_177007	TTTTTTTTTTTTTTTTTTTTTTCCCGGAAGAGACGAAGGAATGCTCAGTGCGCATGCGCGGTCGTTCGCGGCCTT
Cd38	NM_007646	CGGCGGGGGGGAATCTTCCAGATTGTCCCAAACAAAGAATCTTGTGTTGTGGATCAGCTGGAAGC
Gtpbp6	NM_145147	CCTTCCTTTCTCGTGACTCCGCCCCTTCCAGAACTAGGCGTCACCGCCGCCCCAATTCCCCGTGGCCTTCCTGTATAGCACTAGTAAGT
Cit	NM_007708	AGAGAAAACCATACCTGATGCAAACTGCAAGAGGTTTTATTGTTAGCTAGCTGAGGATGAACTCC

Serpine1	NM_008871	TTCCCTTGACTGGAACATCCCTGTGCCTTTGGCTGGTCGGTCGTCTAGACCCTTGGAAGTACCACAAGA
Brca2	NM_009765	GATGGGCGAGCACATGCTAGCCGGGAACAGGCACAGAAGGAGCTGCTCGCTGACTTCCCGGTGGCACT
BC057022	NM_001004180	GGGAAGCAGCTGGCAGCCGATCCGGTTTCCAGGTAAATCCCCGCCCCCACCCACACTCCGGGAGC
Ubc	NM_019639	CACCCAGATCCGTCCTTTGCAGGTGACCAGGGACTACCGCTTCCGAGCCGGCACCAATGGGGACCGAGAAAGGGCCCCAGCAACAGAGAGGGATTGGCTGAG GCCGCCTACG
Dhrs8	NM_053262	GAGCCACACCTTAAAGGACCAGGAAACAAGGAAGGGGACTCTTTGGGGAAAGGAAGAGAAGTAGCAAGAGGGGGATGGGAGG
Golga3	NM_008146	CAATAATACAAACAAGACTGACAAACACTTAGGAACAAGTTGTCAGAACTGTGGTGGAGGCGGT
Coro1c	NM_011779	CACCACCTGCATGCAGTGCCCTCATAGGTCAGACCAGACTGAGCTAGCCTGAATCTGGAGTGTCCGCAGGCT
Fbxw8	NM_172721	GGGACGGACTGTCCATCCGCCGGAAGTGTCCGTAGTCGGCCTGGAGACTTCCGGGAGCGCGTCTTGTAGGTTCCGG
Anapc7	NM_019805	GGCGATCAGAGATCCAGCTCATCCCTGTAACTATCTTTTCTTCGCACTTGGATCTAGCGGGTCACTTGGACGGGCAAGCCATGCCT
Rabl5	NM_026073	TGCAGTTAGCAGGACCAGCTTTGCCTTTTCTCTTTTGGTCCTCCTCAGCTTCCATCGTAGGCCACTTTTCCAGGCTCCCGGAACTGTCAGGGTT
Mospd3	NM_030037	TTCCGGGTCTTCCCGCTCCGATCTCCTGACGGACTCTGCTCTTTGAGAGTCCCTGACCTCCAGGTCACCGAATTGTGTCCCTGTGCCCTTTCCTTGAGCTCGGCAT ATGCTCTGCTCGGGATCC
A930017N06Rik	NM_175522	AGGGATGACTTGGATCCCCGGAAAGCACACATTAACTCCCCCCCCCCACACACACAGAGGCTAGT
Cyp51	NM_020010	GTAGGATGTAGTCTCCGGAATAGCTTGGCCGATTCCCAGCAGGTAGCAGCAGGGACAGTGGCTGAACAATAGAGTG
Serpine1	NM_008871	CATCTTGCCCTTCCTCTTTGTGTGTCTCTAGTTCTCCCCCTGAGACGTCTTGCTCACTGCCCCT
4933411G11Rik	NM_177880	CCCCAAGAATTGCACAGAAACTCTGTGTCCCAGACGCCTTCGCGCGCCACTGCATCTAGGGATCCTCGCGCAAGAACTTACCTCCTCCCAGCAGTTTGGG
Rbak	NM_001045482	CCCCAAGAATTGCACAGAAACTCTGTGTCCCAGACGCCTTCGCGCGCCACTGCATCTAGGGATCCTCGCGCAAGAACTTACCTCCTCCCAGCAGTTTGGG
Rbak	NM_021326	CCCCAAGAATTGCACAGAAACTCTGTGTCCCAGACGCCTTCGCGCGCCACTGCATCTAGGGATCCTCGCGCAAGAACTTACCTCCTCCCAGCAGTTTGGG
Fbxl13	NM_177076	CCAGGAAAAAGCTGCTTGCACTAGGGGCATCCCGCCTGCCTGGTGAAAGGAACCGCAGCACACAGGGTGGGAGGGCTTCCGATTTTAG
2810037C14Rik	NM_026034	CCAGGAAAAAGCTGCTTGCACTAGGGGCATCCCGCCTGCCTGGTGAAAGGAACCGCAGCACACAGGGTGGGAGGGCTTCCGATTTTAG

0610009O03Rik	NM_026660	AGGGAACCTCCCTGGATAGCAGCCACACCCCGGAGGCTTCGGAACAGGCACCTCCTCTTCCGGGAGGCCGTGCTGTGGCGGGAACTGGGTTTGGAGTCTCCCA
D5Ertd585e	NM_027922	TGAGAGGGTAATTACAGGAATGTGGGTGCAGGTGGAAAGCCTCCACACTGCAGCACAGACAGAGCACTCCTTCCCTTGTTTCCCCTCTCTCTATACTCTA
Rfc5	NM_028128	CGGTCTCTCATGATTCCTTGGGTGTGCACCGAATTGGGAATTGAGTGGGGGGTTTCCCCACTAGGTTCTTTCA
Ppp1cc	NM_013636	CAGAGCCGGAAGGAGCTGGTGAGCCTCGCAGCGCGCAGCAGACTGACAAAGTCCACCTTCAACTTGCGCGCGCCAA
P2rx4	NM_011026	CTCGCCGGGCTCTAGCCAAGCCACCTGGAACCAGCTTGAACAGGACACTGGGGACTACTGGACGTCCTGGGAGCTAAG
4930438O03Rik	NM_029112	GCACACTGTGGGAGGAGAGAGCTGACTCCTGCGTGTTGTGTCTGACCTCCACATAGCGAGCAC
Cldn4	NM_009903	TTAAAGCTCCTAGAGGAGGGGCGGAGGCAGAGGGAGGGGTTTCAGTCCCGGAGGCCTCCCTCGGGA
Ars2	NM_031405	AAAGAATGACGGGAAGAGGGTGGAGACGCGGAAGTCTCGCGTGGTTTTGCGACAAAATCGCCCCAGCTTCTCGGAGCTACGGAAGTTGAGCTGGGCCAAGCC AGGCCTTCTGAACGTGC
Bcdin3	NM_144913	GGGGGGGGGGCGCGGAACTAAAGATGTCAGCGAGGATTATGGAACTTGTAGTTTCACTAGGCTTT
Zcwpw1	NM_001005426	GGGGGGGGGGCGCGGAACTAAAGATGTCAGCGAGGATTATGGAACTTGTAGTTTCACTAGGCTTT
Gpr146	NM_030258	CCAGCATTAAGGCGGCCGGCCGTGGCTGGCCAAGGGGATTGTCCGTTCGGACTGGGGGCGGAGCCTGCGGGAACTAAGCACCGGTTCCAAGAAA
Uspl1	NM_001013378	TCGCCGCAGTTCCCACTGCGCCTGCGCCTGCTGGGCGGGCAAGCCACGCCTCTGCGGAGCCCTGCCGGAAGTGCTCTGGTCTT
Skap2	NM_018773	ACCACGCCCACACACAGCCGGGAAGGCCACGCCCATACGCGGAAGCGAGCAGGAGGCGAGGC
C1rl	NM_181344	CTATTCCTGAGTCTGTCAGCAGGTGGCAGACCTGCACCCAGAGTCTCAACTCATTTCCAGTAA
C87436	NM_146170	TGCGCAGGATGCCGGGAAGCGGGTCGCATGGGAAGTGCGTTTTCCAGCCGGCCGTTCCCGGAAGC
Trh	NM_009426	GGGGACCCGCCCGCTTTCCGGAAACCTGGTGGGGGCCGAGCGAGCGAGGAGGCGCGGCTGG
Ube1c	NM_011666	TAGCACTCACCTCAAGGTCTCAGAGTTGAGTATTTCCTGCACTGTAGGCCCTTAGAAGACAGAGCAAGGCAAGCATTT
Arl6ip5	NM_022992	TAGCACTCACCTCAAGGTCTCAGAGTTGAGTATTTCCTGCACTGTAGGCCCTTAGAAGACAGAGCAAGGCAAGCATTT
Tmem40	NM_144805	CCTCAGCCTGCCTCAGCCTGCCTCAGCCTGTTTCAGCCTGCCTCTGCCTGCCTCAGCCTGCC
Nanog	NM_028016	AGCCAGTTCTCTGGACTCCTCCCAGCTCTTACAATTCCTCTCCCGGACGGTTCCTAGAAGACAAAGGCAAGC
Grcc10	NM_013535	CTAGGAAACCAGAGAAGGATCAAAGCCCCTCTCACACACCGGGGAGCGGGGAAGAGAACTGTTTTGCT

Ldhb	NM_008492	AGAGATGGTGAGGCTTGGCCAAGGTCAGAACTTCCCATGCAGCTGCGAGGCTTTTCTCTGTGATGCA
Gm1070	NM_001033462	GAGCCGCAGAGCGGCGGAGCAGCTGGGAACCTGGGATTGTTCTGGAGCTCGCCGGAACTCACCGGAACTCGCCGCCCTGTG
A2m	NM_175628	TAATCCTTCTGGGAATTCCGGCTAACGGGTCAGGAATTAACCTTGGCAGTAACTAGGCCATCAATGACCCCTTC
Pdk4	NM_013743	CGGGATGACGTGGGCCCCCTTGGGATAGATCCCAGGTCGCTAGGACTTCAGGCCCGCCCAGACT
Cbx3	NM_007624	CCTGTTCCAGTAAAGCTCGTGGAGCGCCGCCACCCCGCCCCCACCCCGCGCCTCGGTGGCTTCAGAAAGCAA
8430410A17Rik	NM_173737	GGCTTCCTGACCCTTAGGGGTTTGTGGGAATAGCTAGAGAGTCTCAATTCGGATGGCCTTACACATTGAGGGTGGGCAGCACAGGTTTTCTGGCTTCAGATTCCG GCCTGGGAAGGG
Krcc1	NM_145568	CCTCCAATGGCAAGAAGGACCGACACCCAAGCTCCTACCCACACGGACAGAGCCGGTTAGTCCGAAATGGCGGGAAG
Prmt8	NM_201371	TTTCTCTCTTTCGGGCCATCTGTCAGGCATTGAGGGATAGAACAATGGGGGAGGGAGGCCATACTCAGGAC
Tulp3	NM_011657	GGTAAACTCCAGGGGAGCGGCTCAAGAGTGCTGGCGGCACGGGATGCACAGCGCATTGCAAATG
Ergic2	NM_026168	CGCGATACTTTCCGCGTTGACGGTATGTGATCTAATCAAGCGGCGCGGTCAACCTGAAAGGGGCGTGGCTACGTGGAAACTAAGTGAA
Ergic2	NM_026355	CGCGATACTTTCCGCGTTGACGGTATGTGATCTAATCAAGCGGCGCGGTCAACCTGAAAGGGGCGTGGCTACGTGGAAACTAAGTGAA
Ezh2	NM_007971	CTTGCTGGGTGGAAGCACCACAGTCGCTGTCTTTGTTCTTTTGCCAACGTGCGGTACCTCTCAGGAAAGCCATGGACAGGCAGGCGGAGG
Adipor2	NM_197985	GGACGAGGGCGTGCTGAAAACACTGGGCTAAAAGACGCCCTAGCCGTCCTAGGCCGTTCAC
Ncapd2	NM_146171	GGGGCGTGGCCCCGAGTCTGCGCTTTCCCGGCATCTCTACCGTAGTCGGCTAAGCATGGTCACCCGTTGTCTCTCCGCGGGCCGCCTAAAGCAGTC
Mrpl51	NM_025595	GGGGCGTGGCCCCGAGTCTGCGCTTTCCCGGCATCTCTACCGTAGTCGGCTAAGCATGGTCACCCGTTGTCTCTCCGCGGGCCGCCTAAAGCAGTC
Asns	NM_012055	GCGTGCGGGAAGTTTCATCATGTGGCTGGGACCCAACCCGTGCGCAGGGGCGTGGCCCGCAGTG
Hirip5	NM_020045	GAGAGCTGATGATTGGGCAGATAAAAGGGAAGGTGGAATGGAAGGTGGAGAAAGAAGAAGGAGAAAATGGATGAAGGAGAGGACGCGGAGGAGG
Clec12a	NM_177686	GCACGCCAAATTGGGAATTGAGTGGGGGGTTCCTCACTAGGTTCTTTCACAGCAAGGACGGTCTCTCATGAGTTCTTGGGGGGTCTCGTCATCCTGAGACTTGA ATGA
Etv6	NM_007961	TGCTGCGCCCGGGTCGCCGCGCCGCGACCCGCACACCCCACGCCGTGCTCGCCGGCTCCTACGCCCC

Cmas	NM_009908	GGTTCTCCCAGACGCTTTCAGTACTTGTTAGGACTCCTCCTGACTGGCATACTCCACCCCCTACCCTA
Kras	NM_021284	CGCGAGGGTGGTGCCGAGAGGGCGTCGGCCAACGCGGACCCCGACCCGGGGCTGGCGCTCCCGATAGCCCCGCCTCGCCGCTCGT
ZBTB45	NM_001024699	CATTGGCTTTCCATTCCTGCAAGCTCCAGCTAGGAAGGTTTCCCCACTTACTGGAATGTG
Sema4b	NM_013659	AGCTCAGCCACTGACAAGCCCAGAACTGGTTGTTTCAGGAACCGCCCAGAAAGCTGCTGTCCTTTTCTGGAAGATAGCA
Trim6	NM_001013616	TACCTGCCCCGACCCGGTCTACACCGGCTGCAACCAGTCGGAGCTAGGAGCCCTAGGGCCTCTCCAGAG
Bbc3	NM_133234	CCCTAGGTCTGGGCTGTCCCCACGCTGCTCCTCCTGCCTGGACCAGGCCTCGCCCCGCCCCTC
Aldh16a1	NM_145954	AGGCTTCCGCACTGTAGGGGAAATCACGCGCTCAGAGGGGCGGGGCTTCCGAGAAGGCCGCAGTGGATGACGGGAGCCCGC
Nop17	NM_029406	AGGCTTCCGCACTGTAGGGGAAATCACGCGCTCAGAGGGGCGGGGCTTCCGAGAAGGCCGCAGTGGATGACGGGAGCCCGC
AI428936	NM_153577	CCAGGTTCTTCCAGGAAGGGGTGGAGCTAGGCTGGCCCCTGGGCCCCTCAGTAGCCTCGGAC
Fah	NM_010176	CCACCCCCCCCCATCTCCATCCCGGAACTGTCAGACCGTTCCGCCCCTATGTCTAAACCTCCCC
Nup98	NM_022979	AAGAGGAGGAGCGTAAGGAATTGCCGGGAAAGGGGGGGGGGGGGGGAAAGGAGGGACGATGGGGAGGGATGTAGAGTCCCACTCCCTCTGCCGCAACGGT<br>TGGCTGAGAGA
Arl6ip1	NM_019419	CGCCCCGCAGTCTGCTTATATAGGCTAGCTGCCTTTCCCGACAGCCCTCGCGAAACTCGCTCCTCATTGGG
Hsd3b7	NM_001040684	GGGGTGGAGGCTAGGCTGGTCTCCTTCCTGGAATGGGTCAGGAGGGAGGAGCGAGCGGAAGGAAGGA
Hsd3b7	NM_133943	GGGGTGGAGGCTAGGCTGGTCTCCTTCCTGGAATGGGTCAGGAGGGAGGAGCGAGCGGAAGGAAGGA
Cic	NM_027882	GGCAGCAGCCAATAGGCTTAAGCGCAGGGGGCAGTGCTTGCGCGGAAGGCCGCGGGGGAAGCGGGGAAGG
Zfp36	NM_011756	GAGGGGGGGCTTCCTGGAAGCCGTGACGAGGACGCTCAGCCCGGACGCTTGGGTTCCTTCTC
Fchsd2	NM_199012	CCCCAGCGGGCTGGCGGGAAGGCGCGCGGGAAGCCCCCAGCTGCCTGCTCGGTGTGGGGGGAGGGAGGACG
Sbk1	NM_145587	ACCCGGGTGGCTTTGCACAGCTGCTGCTTCAGCCAGGAACCCCCCACCCCCTTCTTCCGTTCTGCTATACACCTTTA
Leng1	NM_027203	CCCAGGACTACAGCCTCCAACACCTGTCAGCTCAGACCCTAGGCTACTCCTCAGACTCACCTTCTTCAGGCAGAACAGT
6330581L23Rik	NM_146185	CTCCCAGGAGAGGGAAATACAACGCCTGGAAATGTCAGGGGCTGAAAGCTGTGGCCTGAACAACAGATACAA

Ppme1	NM_028292	AGCAAAGCATCATGGGTGTTGTAGTCCATTTGCAGGGGCTCTAATTGGCGCCTTCTTTTGGCACCGCTTTCCGGGAACTGTTG
AU020772	NM_001017985	AGCAAAGCATCATGGGTGTTGTAGTCCATTTGCAGGGGCTCTAATTGGCGCCTTCTTTTGGCACCGCTTTCCGGGAACTGTTG
Relb	NM_009046	TCGGCCGGGAAGCGAAGGCTTGGCACTTCGCAGGGTCCGGGTGCGAGTCGCTGCCCATTGCCT
Ccne1	NM_007633	GGTTTCTGCACAGGCCTGGGGCCCAAGCGGAGCCAATGACATTCCTCAGGAGTTGCTAGGCTGACTGGAAGTGGGCTT
2310044H10Rik	NM_197991	CCCATTTTTGTATTTTTGAGACAATCTCAAGCAATCCAAGCTAGCCTCAAACTCCTTGATCCTCCAGCCTCAGCCTCCCAAGCACGGAGATGAGAGGCTAGCTAC CACTACTCCACGCAGCTCAGGTTCCGAGCAAA
Btbd1	NM_146193	GCACCCGGCGCGCCGCCGCCATGGTCGAGCCGCCCCGAGGCCGGCACCGCCCCCCTGCCT
Mesdc1	NM_030705	GCCACACCCACTCCCTTTCTTTGTGCAGACCTTGTCTGCCAGAAACTCCTGGAACACCTGGGCCTCCTCCCAGTAA
Nupr1	NM_019738	CTACTTATAACGCTGTCTCCTCCCTAAACCACGGGGTTGGCCCCCGCCCTCCAAGCCCTGTCTGATGCAATCTGGCCCTTTCCCA
Zfp629	NM_177226	CTTAACCCCTTAAGTGCCCTGCCTCGGGGGACGGGCTAGATGCCGGGAACTCTAGGGTCCTTCTCAAGGGGTGATTCCAGGCTGAGGACCCGGGGACCAGTTCA GGGTGACTGGGACGGAGCAGGAAGT
B4galnt4	NM_177897	CCAGGAAGCAGTCAGTGTTCCTTCCATGGTCTCTGTTTCAGTTCCTGCTTCTAGATGCCTGCTTGAGTGCACGCCCTGGCTTT
Nipa2	NM_023647	TCGGACAGCCTTCGGCCGCACCGCGGCCAGCACCAGAAGAGCCGCCGAGAACCTGCCGGGAAACCTG
A230056P14Rik	NM_001033396	TCGGACAGCCTTCGGCCGCACCGCGGCCAGCACCAGAAGAGCCGCCGAGAACCTGCCGGGAAACCTG
Pwwp2	NM_001033206	AGAAGCAGAGTGGGGGAAGGGCCTGGACCCGGGGAGTGTCCCCGCTACCCCTCGGTTCCTGGCAAGGCCTCCTAGGGGGCATTCCAAGCCTGCGTGGCGT
Chmp2a	NM_026885	CCTGCTGCGGCCGCCGTTTCCGGATTAAGCGACCTGACGTCACACGACCCACCCTCTCCGGGAATGCTGGG
Npas1	NM_008718	GTGCCTGTCTTCGCCCCGCCTACAGCGGCCCCTGCCTGCTGGATAGTTCCCATTCCGGGAACTATCTGGGATAGTGTTGGCCTGGTGTGTCTGA
Bcl3	NM_033601	TGGAAGGCCCTGTGCCTGGGGACCCAGCAGGGATGGAGGGGAGGGGAAGGGAGGCAGGCCTGGAAT
Erf	NM_010155	GGAGGATGATCAAGAGCACAGCCATTTCCCAGCCTGAGTTTACCCAACCCCCTCAACCCCGAGCCTGAAAGAGGGCAGCTGTCCAGGGGTGGGGTGGAATGGG TGGGAAACATTTCCCA
Zfp36	NM_011756	GCAAGCACTTCCGGCCTAGGGGGAGGTGAACTCTGAGCTGAGTTTCCAGAAAAACAAGCAGGGACGGGAAACGCGGGGGTGTGAGAGCCAGTG
Herc2	NM_010418	TCGAGAACCTCGTCCCGCTGCAAATACCTTACCAACTTCGGGTCCCTGGAAGCAAACACA

Zfp446	NM_175558	GCAGTGGGACGTGCTCGGTGTCACTTCCTGGAAGTGCCCCAACCTCCTGGGGCTCTCCCACGCGTCAGAGGTGAGCTCACTCC
Mark4	NM_172279	ACCTCAGGGAGGGCTGAGACTGCAGGTGTGAGCACCACACCCAGTTGTCTCTGGTTCTGGGAATTGTTTGT
Apoe	NM_009696	GAGACAAAGTCCTCTGCCAGCTCTCGGGGCCAGACTGCACAGAGGCGAGTGACCTGGAGCCACCCTCCTGGTGAGGCCAGGCCTCTGATGTTC
Bcl3	NM_033601	CTCGGGGCTTTCCCGGACATCCCGGGAGGTGGAGCACACACTGGGGTTGAGTCCAGGTCACTGAGTCACAGGGAGGCGGAGGGGGGCGGGGCGGCTTTGTAG ACTTAGAGAGGCAGGGGAATAGCACCGCATGGC
Samd4b	NM_175021	GCGCTGGGGTGAACTGACTGGAAAGGGGCCGGCTCTCCAGAAGGGTGACAGCAACGTGGCTTG
Gmfg	NM_022024	GCGCTGGGGTGAACTGACTGGAAAGGGGCCGGCTCTCCAGAAGGGTGACAGCAACGTGGCTTG
Gmfg	NM_001039192	GCGCTGGGGTGAACTGACTGGAAAGGGGCCGGCTCTCCAGAAGGGTGACAGCAACGTGGCTTG
Nfkbib	NM_010908	AATTCCCCAACACGCCCCCTCATATACCCAATCAAAATGTTTTAAATAGCTACACCCCGCCCCTGTACTGCACCACA
Sirt2	NM_022432	AATTCCCCAACACGCCCCCTCATATACCCAATCAAAATGTTTTAAATAGCTACACCCCGCCCCTGTACTGCACCACA
Capns1	NM_009795	CTTCACGCACAATAAACTAGACTGCAAGGCTGACTTCGTCTCTCTTAGCTTGCCAGAGTCTCTGCCCTCTTACTCCCCCTAC
Chchd8	NM_183270	CTGAAGGCGGGCTCTACACGGAGAGTTCACCAATGGGAATTGCAGAGGGCTCTGTTCTTCCGCGTGTGTGCTCCCGCTGGGTC
Art1	NM_009710	GAGTCTGCCTGGTTCCTGTGCTTTCCTGGCACTGCTATTCCGTATGCAGGCAATGTGCAGCCAGTGTCTGATCCTTGCCCTGGTCCCCAACCCTCTTAG
Cyb5r2	NM_177216	CTTCCTGCCCCGCGCTTTCCCGGCAGAACTCACCTGGAGCGAGATGCCTGACTTATCCTCGAGATGAACAGTGCCTTTGATGGTGGGGATGATGAAACATCCGGC AGTG
Cttn	NM_007803	CCCGCTCACCGCACCAGCAGGGTCCACCAGGCTCCCGCCGCGCCCCGCCGCTCCCTGTGCCC
Ccnd1	NM_007631	CTCAGTGCTGTTACTAGGCGGACCGGGCAGATCTCCACGAGGAACTTCGTTGAGCTCTGCGCTC
Nol3	NM_030152	GAGTTCGCAGAGCCAGTGAAGCATGCCCTTCCCCCACGCCATTCCCAGCAGCCCTGGCCGC
Rbm35b	NM_176838	CCTCCCATTCCGGAGCGCGCCTGCGTCAGTCCAGGCTTTCCGGTAAGGACACTCCCTTCCAAC
Lsm4	NM_015816	AAGCTGCGTTTCCGGGAAATGGGCGGGCTTAATGGCTCACGTGGGCTTCGTTGCCCTCTTG
Hook2	NM_133255	CTCCCCTAAACCGGCTCAACCGGTGGACGCACCCTTTTCCCAGAAGGATGCGTTCCTACGTTCTGCGCGCCC
1700041G16Rik	NM_175196	GCCACAAAGAAGATCTTGTGCCGGTAAACACTCAGGAGGTAGTGGTGAGGGGTGGGGATGACCG

Unc13a	NM_001029873	CCGTAAATGACGGCGGCCAGGAAGCTCAGGGGCCGCACAGGAAGGAGCAGGGACTTTCCTGTCG
Jak3	NM_010589	CCGTAAATGACGGCGGCCAGGAAGCTCAGGGGCCGCACAGGAAGGAGCAGGGACTTTCCTGTCG
Card15	NM_145857	GTCCCCAAGGGCAGAACACAAAGCACTGCAGGCAGGCCTGTTCCCGGATACCTGTCTCACCAGTCC
BC057552	NM_172502	GGCCGGCCGGTTGGGTGACAGCCGGCATTCCTTGTCGCTTCCCGCTACGCTTGTCCATCAAAGCTATCTTGGTTTTTGGTTG
Hook2	NM_133255	CCTTAGGGAGGGGTTGAGTCACTCACTTCCCAGCAGTACAACTCAGGCTGGGTCCAGCTGTTCCTGGAAACATGTTCGAGGTGG
Galns	NM_016722	GGAGTGGGCTGGGCCTTACGGGAAAGGGGCGGGACCAAGGCCGGACCTGAGAGTCTGGGCGGA
Trappc2l	NM_021502	GGAGTGGGCTGGGCCTTACGGGAAAGGGGCGGGACCAAGGCCGGACCTGAGAGTCTGGGCGGA
Large	NM_010687	CCGGAGCCCGGGCCGGGTCTCGGGAAAAGGGTGCTAGCTGGAGCTCCCCGCCCTTCTTTCCCGGACTCCGGCTACCCCAGA
Hook2	NM_133255	GAGCTGGGGAAGGCCCCGTTCGTTCGGCCCTGGGGCCCCAGTTTCGGGTCGGGCAGGAAGGG
Katnb1	NM_028805	CGGATTTTTTAAACACAACCGGGTTCCAGGAAATGCAGGAAAAGGGCGGAAAAAAACCAAGCCT
Thap1	NM_199042	AGTGTGGAGGGCCAGAAGGATGGTGCAGTCCTGCTCCGCCTACGGCTGCAAGAACCGCTACGACAA
Aars	NM_146217	TTGAGCATTGCATCAGCCGAGCCCAGGAGCCTTCCCAGAAACCGGCTTGTTAGCTCATTCGCG
Trappc5	NM_025701	CCAATTCCATTTCACAAGAGTGTTCTTGCTTCACCCAGAGAGGCGTCCTAGGCTCCAGTTCA
Slc25a4	NM_007450	CCTCCTTTGCAGAGGGCATTGGGGGCGAAGACGCTTCCGGGAGCTAGGGGTGGGTTGGGGGCTGTGCCTGCAGCTTGGGACGTTTAAAGGG
Kifc3	NM_010631	GGGGGATGGGGGAGAACTACTCGGGGGACACAGAGGGAGGGCCCAAACAGTAGCCTTCCTGGAAGCGACCCTT
Tssk6	NM_032004	GCCACCAGCGGAAGTGAGGCAACGCGCGACCACCATTTTGAGTGTGGCGGAAGTTCCGTCACCCAGGAGGTTCCCTATAATTTGGAAGCG
Rbmxrt	NM_009033	TTTAAAAAGACTGGGCAAGCAGGCCAGGTGACACGCCCCTTTAATCCCATGCCTGGAGGCAGAGGCAGGTAGATCTCTTAGTTGCAGGCCAG
Asf1b	NM_024184	CGCTGCTCTCTGTGAGGTGCGTCTACCGGAAAAGCGGAACGAGGGGCGGGGGATGCCGGTGCGGTAACCCTTGGGATTGGACAAGAG
Mt3	NM_013603	ACCCTGAGACCTGCCCCTGTCCTACTGGTGAGCCCCTTCCCCCTCCTGCAGCACTTTGCCCTTTCC
Sntb2	NM_009229	GCTTTCAGTGAACCAGGACAACCTCGCCTCCCGGAAGCGGCCTCGCCGGCCTTCCCCCCGCCCCTCCGGAAAGTGGGCGTGGCCCTCATGA
Aprt	NM_009698	CGTTCCTTCCCTCGCGCATAGTACAAGGGGCCTGAGATTCAGCCCCTGCTTTCCTGGAATTAGCCCGGAAGGGA
Dlc1	NM_015802	ACCGCACACCAGGCCCCGCCCAAAGCTCGCCCAGGTCCCGCCCCACGCCTCCTTCCCCCTTTCCTGG

Ssbp4	NM_133772	CGCGCAGCCCTTGCTCGCCTTGCCGTACATGCTCCGCACCTGTCAGGCGCGCGTGGCCATCGCTGCCC
Ssbp4	NM_133772	CCAGGGCTCAGCTGGGCCGACGCCGGCCTTTCCCATTTCCTGCTTGGCCCGCCACCCCCTC
Mtap1s	NM_173013	CCAAAGCCTAGCGGGCATTGACAGAGGGGTGGAGAGGAGGGGGCGCTTCCGGCCGGGGTCTCCGGTG
Elmod2	NM_178736	AGGAGGGATACCGGGAACTCCAGCCCTGGGATTCGCAGACACACCCTCTCTGCGCCCGGGCCCGAGGAGCTGTCAAATTCGAAATCCCCG
Dpep3	NM_027960	GTGCCTGAAAGTGCTGGTGCCTTCAGTGGGGTCAAATATTCTGTGATGTGCTAAGTTCTGTGTAGAGGAGTG
Nfat5	NM_133957	TTGATGCTGTTCCGTGGCGCGCCTGTCCGCCCTCCGCCGGCCGCCAGGCTCTTCCGGGCTCCCGGACCTCCCCCCCTCCGC
Nfat5	NM_018823	TTGATGCTGTTCCGTGGCGCGCCTGTCCGCCCTCCGCCGGCCGCCAGGCTCTTCCGGGCTCCCGGACCTCCCCCCCTCCGC
Clcn3	NM_173876	AGGTTGCATTCTCCCATACCAACAAGGCAGGGCAAGTAAGCAGCTCACTGCAGCACGGAGGACAACAGCCAGCCTGTTCCCA
Armc6	NM_133972	GCTCCTCACCGGGTCTACGCAGCCACGTGGGGAAAGGGTGGGGCTCGCAGCTGTCACCCGAGACTT
Sfrs14	NM_172755	GCTCCTCACCGGGTCTACGCAGCCACGTGGGGAAAGGGTGGGGCTCGCAGCTGTCACCCGAGACTT
Nr2f6	NM_010150	CAATCTCAGCAGGTGCCCTACTGCTGAGCAACCACCAGCCCTCTACTTCCTGTAAATGCTGGTTCTAAAGCAGCCTGGGGT
Ier2	NM_010499	ATTCAAATAACCCCTTTCATTTGCTGTGCAGGCCCTTTAAGAAACACCCCCTGCCAGCGGGCAGACTTCCCGCCACTGCAGCCGGCCGACTTCCC
Junb	NM_008416	ATACTGAATTTGTCAGCTGCGGAAGTGCACTCCGATTGGCCGACGCGGGAGCCAGGCCCCCGCGGCCCCCTCCTAGAGCGTGGGGAAGGGGAGGAGGCGGCTC<br>GCGTCACTGTCAGGAAGCGCGTGTCCTTG
Mt1	NM_013602	CTTCTGGTGTGTCTGAAGTCAGCTACAGTGTACTTAAAATAATAAATCTTAGCCGGGCGTGGTGGCGCACACCTTTAA
Mt1	NM_013602	AGCCAGTCGTGCCAAAGGGGCGGTCCCGCTGTGCACACTGGCGCTCCAGGGAGCTCTGCACTCCGC
Gfod2	NM_027469	ATCCAAAGCACCGATACCGGGAACACGCCGCCTGGTTTCTAAGGAACAGGGGTCTCTGGTAC
Vps4a	NM_126165	GCCCTTTACCGGTATCCCACCAGACCCCGCCCCCGGCAGGCCCCGCCCCTCTTAGAGGAGCGGTGTCTGCAGTTGCAGGCCATGGCCAC
Sf3b3	NM_133953	GAATCGCTTCAAGGCCTAAGCCACCGCCACTGCCGATCCGCCGGCGTCCGCCATTAGCCTTCAAGACGGGCCTTCGGGTGCCGCGCGAC
Cog4	NM_133973	GAATCGCTTCAAGGCCTAAGCCACCGCCACTGCCGATCCGCCGGCGTCCGCCATTAGCCTTCAAGACGGGCCTTCGGGTGCCGCGCGAC
1700030J22Rik	NM_027103	CAAGTCGCGGCCCCCAAGAGCCGGGCTGTTGCCCTAGGTTACGGCCGCGTCGGAGGCGGGCCTAGGTGCAGGCATGCCGGAAGTGACGTAACCCC

BC019943	NM_144927	CTCTAATCTACACAAGAACCAGAAGTGGAGGATCCTCCTAAGTTCATGTCAGACGGGTAATGTGCCCACGTCGTAACAAGGTTTATGGGCGGCAC
Gsr	NM_010344	ACGCGCTGCGGGGCGGGGACAGGGGCCCTCGGGCGCGGTGCTTAGTCACGGCGACGCTGCACCC
Zfp42	NM_009556	AGAATCCCGATCGCCTGGCCTTGAGACTCAGTCACATCTCAGTACCTCTGTTACAGACTTGTAACCGAATTTACACAAG
Hmgb2	NM_008252	CCTCTCTAGACCGGTTCCAACGGGTCTGGGAGTTAACGACCTGGGAGACCCACCGAACCTGCTAGGCTTAGCCTGGAGGGACGGGC
Arrdc2	NM_027560	GGCTCGTATTCACTCCTGAGCCCAGCTGTGTGAACGAACACAGGTTCACAGCCTTATGAACCAGGCTGCAG
Gcsh	NM_026572	CGCAGGCTGTAGGCCACAACCCGCAAGCTCCTCGACACTTGCAGCGACATGGTCAGAGGTCCGGAGGGTAACTGCGCTTCGCCTCCGCCACCGGGCCGGT
Taf1c	NM_021441	GTCTCTATAGCCACGTGGTCTGCCACGTGCTCTCAGCATTCCCGCAACAGCCTCACCTGACCCCG
Itgb1	NM_010578	GAGTTCAGGGCAGATCTCTGTCACCTGACTATTTCCCAGAATTGCCCCTCCCCCTGCCTCTGCCTCTCTGCCTCACTGCC
Tdgf1	NM_011562	TGGTGGGGGGGGGGAATCTGCTATTTCCGAGAAGGCTGGGCCTCCTTCATTAACAAGCTAATGG
Lrrc2	NM_028838	TGGTGGGGGGGGGGAATCTGCTATTTCCGAGAAGGCTGGGCCTCCTTCATTAACAAGCTAATGG
Lrrc2	NM_028838	GAGGGACATCAGGGATTTTCCTGGAAGAAAGCTTTGCTGGGCGTTCATGTGACTCATGTTC
Icam1	NM_010493	GCCCTCGTTCCGGAGGGGAAGGCGCGAGGTTTCCCGGAAAGTGGCCCCGACAGCACCGCCCCT
Tdgf1	NM_011562	AGGACCCTCAGGTGGGCGGGGAGACGGGGCCCCACGCCCATTACGGGGCCCCACGCCCAT
Cmtm6	NM_026036	GAGGGCGGAGTTGACCGGAAGCGTGGGCGGAGTCCCAGGAAGTGACGCCCCCTACCGCTCG
Loh11cr2a	NM_172767	GTCTTCCTGGAAGTTCTGCTTTAGTTACTTTCACTTTTAAAGCTGCGGAGACTTTAGGAGGT
Tmem24	NM_027909	GGGTGGAGTGGGGGAGGGTTGAGTTGCACACGTAGAGGATTCCGGGATGAGAATAGGCGGAGTCT
Gtf2a2	NM_001039519	TTCCCGAGCGCCAGCAGGAGAGACCACGCCCCTTCTCGGAAACGGCGGTGGGGGCCGGCAGTGGCTCCTGTGGCGTCACC
Vill	NM_011700	TGTTGCCCACAGGCTGAGTGAAGGAAGGTGCAGAGGCAGTTCCCAGAAGGTAGGCCTGGACTGGGGTGGGGGTGGGGGACCCGGTAGAGGGGTTGC
Pkm2	NM_011099	GTGGTAAGGATGTGGCCATCAGTGGGGGAGGAGCTTCCTTCTGCCTTTAATTCTATCTGGG
Smad6	NM_008542	GCACCACGCCCCTTTCCGGGCAGGAGCGGGGCCCCGAGCTCAATGAAAGAAATTCCGCTACTCTCATTGGCCTGG
Plekhq1	NM_153119	AATGGACGGAAGAACCTAGGCGGAGCTGAAGTAGCAGGAAGAGGGCGGAGAGAGGTTTCCCGGACCAAAGCAGGGAGAAGGACTGGGAGGGGGTAGAG

Plscr1	NM_011636	CCATTTCATTCCACCGGAAACACCTCTCATCAGGCCTTCACCTTGAGTGCCTCCTTTCTCATGCTTTAG
Stag1	NM_009282	GCGCCTCAGCTCCCTCCCCTTTCCGGGCAGGCCCGTCCTGTCAGCGGAGCAGTGAGCAGCGCGGCCAGCAGAGGGCGGTCGGG
Cish	NM_009895	GTCTCACGTCCAGCGATACGATTGGTCAACTCTAGGAGCTCCCGCCCAGTTTTCCTGGAAAGTTCTTGGAAATCTGTCAAAGGTGTTTCCTTTCTCG
Trim71	NM_001042503	GTTATTGGGACTGATCGGAATTCGGAATCCTTTTAATCTCCAAAGGAGCAAACTCACTTCC
Arhgef12	NM_027144	GGCGCGACTGGAGCGCGCCGAGCCGTCCCCTCCCCCGCCGACTTTTTTGGGCCCTTCTCCCTGGGACTTCGGGAAGCCGCTTCACCC
Scap	NM_001001144	CGCGCCGCTCGTGCCCGGGAGTTCCGGGATGCGGGCGAGCCCGGGCAGGGGCGCGCGGGCGTGGGCGC
9530077C05Rik	NM_026739	GTCCGCGTTGTGCACGCTGGGAGTTGTAGTTCGCTTCCCTTCTCTCCCTACGAGCAGGGTGTCAGTCGCAGGAGCGCCTCCAGCGGCGAGCATGTTTGGTGGCG TGGAGTGCGG
Bcl9l	NM_030256	GAGCCTCTAGGGCTCAAGACTCCTCCCCTTCCCTCCCCCACCTTCTACCGGGATCTCTGCTGGGGGCGGGGAAAGGGGACAGAGCAGGGGGCGGTTGCTTGAG GTGTCT
Suhw4	NM_146224	GGCGCATATGGCCGCGCTCGCCTCCCGCGTTGGCGGGAAGACTGGGAGCGCGGACGCGCGAGGC
Parp16	NM_177460	CCGGGGCGGGTCCCGGGGCGGTTTCCACTTGGGGCGGTGGCGAGGATGACTAGCAGCCGCATTGGGCA
Ttk	NM_009445	AGAAGCGTTTCTCCTAAGAGCAGCTGAGCTCATAACTGAGACAGCATCACGGGGATGGGGGCGGGGGGGGGGAA
2610101N10Rik	NM_026476	CTCCGCGGCGCCGGGGCCGCGTGACTTCCGCGTCGAGGCGCTTTTACGCGCCCCGCCCACCCCG
Slc25a36	NM_138756	CTTCTCCAGTAAGGCTTTACCGCCGAACCTCTCCAAACAGTGCCGCCAACTGAGGACCGAAGTATCACATGCCGGAACTATGGGGGGCATTCTCATT
Icam1	NM_010493	TCACTGCGAAAAGCAAGAGCAGTGGAGCACGAGGCAGGATCGGGAGTTCAAGGTCATCCTCAACTACAGGGCGTTTGAGGT
Spbc24	NM_026282	GTTTCTAGCTGTGCAACTTGTGCCAGTGACCTTGGCACAAGATCTGGCAGGTTCCTACCAGATC
Hspa8	NM_031165	GACCGAGGGTCGTGTGGGGGAGGGGCGCAACAACCTTCGCAGCCATTTTGTCCTCGCCCCACTTCCGTCTT
Gclc	NM_010295	CCCCGCGGGGAACGCGGCAGCCTCTGCTCGGGACCGGACCCCCGTGGGCGGCCAGCCCGCGC
Parp3	NM_145619	CTCCTAAGAAACAAGTTCCAAGTCTGGGATCCCATCTAGCGACCCTTCGTGAGCCTTCAGCTAGTTTTT
Rnu3ip2	NM_145620	CTCCTAAGAAACAAGTTCCAAGTCTGGGATCCCATCTAGCGACCCTTCGTGAGCCTTCAGCTAGTTTTT

Ube1l	NM_023738	CTTCCCTCTCACACCGCGTGCCTCGTGCTGAAGACAATCCAACCACTTGCCAGGAAATGGGAGGAGCCTGAGG
Dalrd3	NM_026378	TGGCGACGGCGCAGCGCTCCATGAGAGGCGTGGCCGAGCATGGATTGGCTTCCCGGCAGCGCGAGGGGGTGTGG
4733401H18Rik	NM_023247	TGGCGACGGCGCAGCGCTCCATGAGAGGCGTGGCCGAGCATGGATTGGCTTCCCGGCAGCGCGAGGGGGTGTGG
Myd88	NM_010851	TCCAGTACGCTCGCCTGTGGAGTTTCCTACTTCCCTTAAGTCTCCTGCCAGCCCCTCCTTCTTTCGCTTTCCGAGAAGCTCCGCCCTTTGCTAGAATCTAGACTAC GGGGCCAGAACTACCCAGCCTA
Acaa1a	NM_130864	TCCAGTACGCTCGCCTGTGGAGTTTCCTACTTCCCTTAAGTCTCCTGCCAGCCCCTCCTTCTTTCGCTTTCCGAGAAGCTCCGCCCTTTGCTAGAATCTAGACTAC GGGGCCAGAACTACCCAGCCTA
Sec63	NM_153055	AGGTTTGGCAACCTCGCTTAGGTTGCAAAAGCTCAGGTCCCAATCGCACCTAGGACGCTCGCCTCTGCTC
Socs2	NM_007706	GCCGAGTGTGCGCGCGACTTCCCAGCCCGACGCGCCTCCCTCGCTCCCCGCCTCCCGCCCCTCGCGCC
Tbc1d15	NM_025706	CGGTCGGGCTAAAATAACATCCGGGCGGGAAAGGGGCGGGGAGGAGCGGGAACAGCAGTTAGGCGCATGCGT
Dnmt3l	NM_019448	CCTCAGAACTCTCTCCCTCCCCCACCCTTTCCCCGAAGCCCTCTCCCACTCTCAAGGCTT
Cd63	NM_007653	CCGGCTCCTGGCTCGAGCCTTCCAGGCAGCGGCCACGTGGCCTTGTCCTGGCGGGGGGCT
Cdc2l6	NM_198164	GTGCTTCCCGCTTTTCTAGCCTAATGACAGTCAGGGCCGGGTATCTACTTCCTAGCTCAGCAAAGGTG
Crsp3	NM_027347	CAGCGCTGTCAGCTTACGGGGTCACCTCTACGAGCGGAACCAACAGGCTGAGATACAGATTTCAGAGGGGCTGG
Suhw2	NM_177475	AGAGAGAGAGAGAGAGAGAGAGAAGAGCCTTTGGGGTGGGGGAAGGGAAGGGCAGCCCAGCCCCTGGGC
Ikzf4	NM_011772	GGGTGAGCCCAGCCACTCACCTTCCAGGAAGCGTGGATTCCTGGAAGTGAGGGGAACCGCCGTCATTTCGCAGGCAGTGGCAGCCGCACGGTGAGCAGGGGC CCACGGCTGCAGCCGG
Tfam	NM_009360	CTCTCTTGAGGGTAAAGGGGACTTCCAGCTCGTCCTTTCAAACCTCCTGTCACAACAGCAACAAGA
Ptges3	NM_019766	AGCGGAGCGAGGCCGCGCCGGGAGCCCCGGCTGCCGGGAAACGGCCCCTCGCCCTCCCCCCACCCTCGGCCCTTCGCCTTTCTC
Pdss2	NM_027772	GGGGTGCCAAAGGGCGGACACCGACGTCCTGGGGCACAGGCTCCCTTCCATCTCTCGCCTTCCGG
Midn	NM_021565	TTCCCGTTCCGCCCCCCCCTTCCACACGCGGGCGCCCCCAGGCGGAAGCACCAGGCCAACAGGAAAGGGGGCGGAGCAGGGAAGCAGGCCAAACACCCCCCC AATCCTAATGTGAGGT

Syn3	NM_013722	CTCCCTAAGGCCAGAAAACCCACCTACTCCACTTTGCCTTGGCTTACCGACAGTGCCAGCTACGGTGGCCCGTGTGTACCTCGGACTGGCGCCGCCAATTACAGGATCTGCGGGAGCGCTG
Helb	NM_080446	TTTCCAAGGCTCTGCTACACAGTTTGAATTACTACCAGTAACCAACTGGAGACAATTGTCTTACTTAGTTG
Mbc2	NM_011843	AGGGTTGGATGGCGGGAAAAGGGGGGGAAAGAAACCAAAAGACAGTATTTGACTGGTCACGGAGAGAACGGGGCGTAGCGTC
Ndufs7	NM_029272	GGAACTCTTTGGAGGACGGACCGCAGAGGTTGTCTGAAGGCCGAGGCCAAGATGGCGGCGCTGGCGGGTG
Ap3d1	NM_007460	GGTGGAGCTTGAGCAGAGGACCTCAAAGCCCGCCCCCAGAGTGACACACTTCCTACCAGGTCACGCCTAGTCCAGCAGGGCCACACCTCCCAACAGTGCCACTTCCCATGGGCCA
Mgat4c	NM_026243	ATTATTTCTCAGGACAGTTTCTAAGAGCCCCTAACAATAGCATATTTGCATACTGGGTTCCAGCAATGGTCAGGGTGGGTCAGGGTGACTTTCTTTGAATGAACACTCCTTTAACCCAGGAAGCAGGTGGGTGGAGGAATGTCACTGTTTTATGAT
Stat3	NM_011486	GGGGGGAGGGAGGAGACATTAGCGGAATGTCCTGCTGAAAACTCAGCTGAGTTCCTGGCAGT
Stat3	NM_213659	GGGGGGAGGGAGGAGACATTAGCGGAATGTCCTGCTGAAAACTCAGCTGAGTTCCTGGCAGT
Stat3	NM_213660	GGGGGGAGGGAGGAGACATTAGCGGAATGTCCTGCTGAAAACTCAGCTGAGTTCCTGGCAGT
Socs3	NM_007707	TGTAATGTTTAGTCACTACTCTGCACTGAAAGGCTGTGCGCGGAGGGCGAGGGAGGGGCC
6030413G23Rik	NM_178664	CGATGCACAGACTGGACCGCTTCCGATTGGTGCAGAGCCACGTGGCTCGCTAGGGACTTACGGGTATGGGAGGGCTGTTAGGGGAACAG
Hoxb13	NM_008267	AGACTCTTAACTTTCAACTTGGCCTTGACCTTCCTGGAAGTGTCTGGCGGGTATGTAAAGG
Cnp1	NM_009923	GTTGTGGGAAGATCTGATACAGTGTGACAGTACCATTGTGGGAGGGTCTGATACAGTGTGACAGTACCAGAGCAGT
Arhgap27	NM_133715	ATGCCATCCACATACATGAGAATACAACTACACACCATCCACATACATGAGAATACAACTACGTGCCATCCACATAGATGAGAATAC
Cobl	NM_172496	GATATGACCCTGGACCTTCCTGGACATCCTATATCTACTAGAATAAACCTAGACCAGATACAGGGT
Tha1	NM_027919	CTCTGCCTGATTCCCGAATTCCAGTCCTTAGAAAACAAACCCTTATCTCATTGTCTGGCTCCAGGCTTCTGCTGGG
Mllt6	NM_139311	CAGGTCAAGGACAAGTTCTCCCCCTCCTTGCTTCCCCCCTCCTGGGCGGGGACTCATCTCC
Nf2	NM_010898	AGTATACTCCTACTCTAGCAAGACTGGTTGAGAAGGAGCGGGCGGCGCCTTTCCCGGGAAACCTGTAGAG
Upp1	NM_009477	GTTTAATTGGATTTGAAAGGGCCAGTCTTTTCCGGGACTGGAGAGCAGGGAATTTCTTGTCTCTTGAGTTGGTAAA

Upp1	NM_009477	TCTGTCCTTGGGTGACCTTAGCCGAGAGTGAGAGCAGCTTCCCGCAATAAACCAGCATTGAATGTTATCTA
Rtn4	NM_194052	GCCGGGAAGGTGAGTCACGCCAAACTGGGCGGAGAGTCTGCCCTCCTCTCTCAGTTGCTCA
Rtn4	NM_194053	GCCGGGAAGGTGAGTCACGCCAAACTGGGCGGAGAGTCTGCCCTCCTCTCTCAGTTGCTCA
Rtn4	NM_194054	GCCGGGAAGGTGAGTCACGCCAAACTGGGCGGAGAGTCTGCCCTCCTCTCTCAGTTGCTCA
Rtn4	NM_194051	GCCGGGAAGGTGAGTCACGCCAAACTGGGCGGAGAGTCTGCCCTCCTCTCTCAGTTGCTCA
Psmd3	NM_009439	TCCTAGAAGATCCGCCTTCATCGTGCACCTCGGCTGTTGATTTATTTTCCCTCCCTGGAC
Map3k14	NM_016896	GGCTGCATTCTTTTTGAGGTTACGGGAAGGAAGGGCGACTGTACGCATGCGTGGGCATTCCCGGC
Vdac1	NM_011694	CTCCCTGTACGCCCCTGTGACTCAGCAGCTTTCCCTGCCTGGCTTTAACTCCGGGAATGGGCTGGGTGTTTGTTGCCGGGTAGATAGAGCTGCAGACT
Pipox	NM_008952	TTTGTCTCAGGACTTTGCCCGGAATTTCAATGCCTCCCAGACCAAGCTGGGCCCCGGGAATGCTGCCTCCCC
Nme1	NM_008704	ACAAAACAAAAAGGTGGGTCTGTTGACGCAGCCCTTCACTTCCAGGAACTTGGCAGGCAGGCTAAGACAAAAGGATCACAAGTTCAGT
Irf1	NM_008390	TGGCGCGGGCCCGAGGGGGTGGGGAGCACAGCTGCCTTGTACTTCCCCTTCGCCGCTTAGCTCTACAACAGCCTGATTTCCC
Tcam1	NM_029467	GCCCTTGGGGGAGGCGGCCTTTTCCCGGAGGAGCGGGAGCGGAAGCTGCACGCGGAAGCGCGGAGG
Ddx5	NM_007840	CAAAGCACGCTGGGAGCCGCTCTATGACCTAATCACCCCGCCCCCCTGGCTCGGAGGCCCTCAACGCC
Ccdc45	NM_177088	CAAAGCACGCTGGGAGCCGCTCTATGACCTAATCACCCCGCCCCCCTGGCTCGGAGGCCCTCAACGCC
Vdac1	NM_011694	GTGTATGACCGTGGGCCCGAGAGCGCTCTTGAATAAGAGTCCTCTTGCAGTTTGCAGCAAGACCGTTTCT
Asb16	NM_148953	GCGAATGGGGCGTAGTTTCCCAAAAGTCTCATCCCTTCAGTCTCCACTTCCAAGGTCAAA
Pnpt1	NM_027869	CAGAAAGCCTGCAAGGATTGCTTTCCCGGCAGTTTGCAGGCTCCACGGAAACGAAACTGCGTCCGACTCCGCCC
Gfpt2	NM_013529	TTGGGCCAGGTCTTTTCTCAGGAGATGAGGGGGCTGGAGAACAGAGAGGAGAGGAGGAGACAGCCGGGAA
Trim25	NM_009546	TCTTTGGTCATTTGAGCCTACACACAGTAGGCCTCTGGCTCCCCCACCCCCACCCTTAACCTTTGATCTGCACACATTCCTGCACTAAGCT
Socs3	NM_007707	CTGGCTCAGGTCTCTAATCCCTGAGATCAAAAGGTAGAGGCAGGAGGACCAGAAGTTCAAGGT
Xpo1	NM_001035226	GGCCCAGGTTCCAAACGCTCCGCGGCGCCATGGGCTGAAAACTCAACCGAGATGGAATCTCCCAGTCTGAACCGGT

Xpo1	NM_134014	GGCCCAGGTTCCAAACGCTCCGCGGCGCCATGGGCTGAAAACTCAACCGAGATGGAATCTCCCAGTCTGAACCGGT
Cyb561	NM_007805	CGGGTTCGGCGCCCGGGTTCCCGGTCTCTAGCTTCTCGCTCGCTACTCTCCACGCCCAAAC
Slc16a6	NM_134038	CGCCCGCGCCCCGGCCCCGCTTCTCTCTGCCTATCTCTTATCCTATACCGGCTGCAGCCGGTCTCCGCCG
Slc16a6	NM_001029842	CGCCCGCGCCCCGGCCCCGCTTCTCTCTGCCTATCTCTTATCCTATACCGGCTGCAGCCGGTCTCCGCCG
Rnf185	NM_145355	TGAACCTCCGACCCCTCCTACCGGGAACTTGGTCACCTGACGCGGAGCCGCCTCTAGTAGAACAACTTCCGGTTCCAGCCGGAAAAGGC
8430429K09Rik	NM_027820	TGAACCTCCGACCCCTCCTACCGGGAACTTGGTCACCTGACGCGGAGCCGCCTCTAGTAGAACAACTTCCGGTTCCAGCCGGAAAAGGC
Fignl1	NM_021891	GAGGTGTAGCCTCCGGGCCGCCACTCCAGAGCCTGCCCGCCCCGGCGCCCTCCGGCCTTAACCAGTTCAGCAGCAGCTATCAAATAGTGAGACTCGAATACAGGAAGG
2010316F05Rik	NM_026465	GCCCGCAGCTCCGAGCCTGCCATTTTCCCCTCTACGTGGGCAGGCTTACCGGAAGTGCCACGGCTTCGG
Scgb3a1	NM_054037	GGGAAATTGTGTTTTGTTTTTGCCATTTTTCATCATTGGTGATCAAATCCAGGGCCTTCCTTTTGTGAGACAAGTACTCCACTGGTGTGCTCTACGGTTCAGCCCAG
Scgb3a1	NM_170727	GGGAAATTGTGTTTTGTTTTTGCCATTTTTCATCATTGGTGATCAAATCCAGGGCCTTCCTTTTGTGAGACAAGTACTCCACTGGTGTGCTCTACGGTTCAGCCCAG
Wdr79	NM_144824	TCCAGGGATGCCTTCTTATGCAAATAGCCTCCCTGCTCTTGCAATCTCTTTGACTGGATTTAGTGAAG
Eif5a	NM_181582	CCACTGTGCTGCTTGCGGCGTCAAGAACGCATGCGTCTGAGCAGGCGCCACACCTCGGCTTTAACTGGAATTGAACAACTCACAAACACGTTTCTGAGAACCTTTCTA
Nr1d1	NM_145434	GGCCGCACGCGGCACTGGAGCAGGTACCATGTGATTCCAGGGAGCGCCTCGTGCCCCAGTGACACACTTTTCCAGCAGCCGGCACGTTGAGAT
Fasn	NM_007988	CGCGCCTCCCTGAGTTTCCGGGACCCTCTCCCTCCCTCCCTTTAAATTCCTCTTTTCAAAGGGTT
4930412M03Rik	NM_177098	ACCGGCAGCCAAGGCCCCGCCCCTGGTAGAGCCGGGCCCCTTTCCGGGATTGCAAAGCCG
Ndp52	NM_029755	CCCCACACCCTGCCCTGCCTGTAGACCCTGTCTCAAGGTCTGAATTCCGGGATCCAAGGGCTCAATTGCA
Tmem106a	NM_144830	GGCGTTGTCACCTGGTAAGGCGTCCCTGGGACCCCATCTCTTTACCCTTTTCAAAGCAAG
Rps27a	NM_001033865	TTAGAACTACTTGTCCCAGGATGCATTTCCACACTTCCCGGATACCTGATTACACTGTCTAAGCTGTGGAGTGAGGGTTAGGAGAGAGTCAGGTGAG

Rps27a	NM_024277	TTAGAACTACTTGTCCCAGGATGCATTTCCACACTTCCCGGATACCTGATTACACTGTCTAAGCTGTGGAGTGAGGGTTAGGAGAGAGTCAGGTGAG
Alkbh5	NM_172943	GGCAGTCCCGGGACGTGACTTGTGGAACGTCCGCTTCGGGAAGCGATGGCGGCGGGAATGGGGTGTGGGTGCCC
Rpain	NM_027186	CAGAATCCTTTGCGCGCGGCTAAGGGGCGGTGATTTGGGCGCTGTTTCCCGCTAAGGGAGGAGATGGCAGAGTCCTCGGGGTCTCCGCACCGCTTGTTGTAC
Nup88	NM_172394	CAGAATCCTTTGCGCGCGGCTAAGGGGCGGTGATTTGGGCGCTGTTTCCCGCTAAGGGAGGAGATGGCAGAGTCCTCGGGGTCTCCGCACCGCTTGTTGTAC
Flot2	NM_001040403	CCCTCTTTTCCGGGTGAGGCAGACCCGTTAATTCCGAGCAGGCTAGGGCTTGCCAGGAATTACTGCCATCACCCGCCCTGCAAACGCGGGTGC
Flot2	NM_008028	CCCTCTTTTCCGGGTGAGGCAGACCCGTTAATTCCGAGCAGGCTAGGGCTTGCCAGGAATTACTGCCATCACCCGCCCTGCAAACGCGGGTGC
Cdc42ep4	NM_020006	GAAAGTTCCCTAACTACTTAAAGGAAGTGGGGGTGGGGTGGTGTAGGCCTCAGCCTCCTTTTTCCCCAGAGTTGCCTCACGGTCCCTCAGCT
D230014K01Rik	NM_172573	ctcctttattttacaaattaaggtgggaagcaggtttactagaaatcacccgagtgctgactcactccttgttggcag
Fos	NM_010234	CCCTGCGCTGCACCCTCAGAGTTGGCTGCAGCCGGCGAGCTGTTCCCGTCAATCCCTCCCTCCTTTACACAGGAT
Mycn	NM_008709	TACATACACAGCAGGGGCTGTATGGTAAGTGTTCCTGGATGCCGCTGGAGGTAGGCTCCATTG
Odc1	NM_013614	GGAACCGATCGCGGCTGGTTTGAGCTGGTGCGTCTCCATGACGACGTGCTCGGCGTATAAGTAGCGGCGCGTCG
Sntg2	NM_172951	AACCCCGCCCATCAGCTCTGCCCTTCCACCTCCTGTCAGTGAGGGGTGAACTGGTTTGTG
Arf6	NM_007481	CCAACTCGAGTACAAAAAGTGCGGGCTACTTTGGCGGATTCTATGGAACACTGGCCTCCCA
4930534B04Rik	NM_181815	TGAACTAGCCGCCCAGGCACCGCTAGAGCACAAGACGCGCCGCCGCCGCCTGGCCCTCTGGGAGCTGTAGTCCATCCCACACTCC
Smoc1	NM_022316	GGAATTCCCATGGCCCGAGCTCCCGCATCCAACCTGCTGTCGCCGCGGCCACGCCGAGCGCAGCAGGCGCCACGCGCAGAGCACACGC
Adck1	NM_028105	GGTGGCGAAAGCACTGTTTCCTGGAATTTGGGTTCCGGGCTGCGCGGCGCAGCTTCCCTACCCTCTGGTGAGTAGCACCCGCGCACAGAGTCCTGGCCAAT
Ankmy2	NM_178910	TGGGTGGCCAGGAAAGGCAGAGAATTCCAGGAAGAGAAAAAAAGAGGAACAAAGGGTGGAAA
Ankmy2	NM_146033	TGGGTGGCCAGGAAAGGCAGAGAATTCCAGGAAGAGAAAAAAAGAGGAACAAAGGGTGGAAA
Twistnb	NM_172253	TTGTGGACTGCAGATGAGTAGCCTGGTGAAGGTCAGTTCCCAGAGTCCTGCCAGTTGTTTCCGGGAGAATGATCTGTCCTCCGGAGAGCACTGGGTGTGAGCTG TGC

1200003C05Rik	NM_024205	GAAGCAGGTTTACAGGAACTCATCTGAGTGCTGACTCATTCCTCGTTCGCAGTCCCTCACCGGAGAACAGAATTG
Ckb	NM_021273	AGAGCCACAGAGCCGCCGACCTTCCTCAAGGTGCCTGCGGGCATCTCCTGGGTGTTCTTGGTGCAACACCAGAGG
Brf1	NM_028193	TGTGCGGCCAAGGCCCGGCCCGTGGAGCCTGCACTGATTGGCACAATAGGCTTTGGGAGCGTCCTGGGCTGGATGTAGTGGGAGTAACTTGGGGAGTCAGCAA GCCCTAGAGGGCAGGATCAAGGACTGCCGGCG
Nup153	NM_175749	GGAGCCTCTGGGAAATGTAGTTTTTCTGGCCAGAACGCGGATTCTCGGAAAGGCAGGCTA
Map3k1	NM_011945	TCTCCCCACCCCCAACAGATGCTCCCAGCTGCACGTCCCTCTTCCTGGCAGTCAGGTGACA
Tmed9	NM_026211	GGCCAAGCAGCCCCTTTTATAGAGCCAGGACTACCTGACTGTTGCCAGGTAACTGTGGGGCG
Il6st	NM_010560	GCTCAGCCTCCGGAATCCCGGACGCCGGGAGCCGCGTTACGGGAATCGCAGAGGCCTGCAGTCCCGCCCCTTC
Hist1h4a	NM_178192	ACATCACAAACCCCGCCCCTCAGCCAAGACCCGAAAGCGCGCCGGCGGGAAAGCGACATCACTGATCCCTCCTCT
Hist1h1a	NM_030609	ACATCACAAACCCCGCCCCTCAGCCAAGACCCGAAAGCGCGCCGGCGGGAAAGCGACATCACTGATCCCTCCTCT
Cart	NM_013732	TTGAAATCGGGTGCCGGGAAGAGGGAGGGGGAGAAGAAGGACGGGGGTGTGGAGGAGAGCCCGGG
Hist1h2bb	NM_175664	CCCTCTTCTGAGTTTCTCATTTCCTCCTGGGTCCTGGTGAAATGAACTCTAGTTTCTGGAAATACCTCACTGA
Ippk	NM_199056	CCGCGTTTCGGAAGTGAGTCACTCTGCCAGCACATAATATGGCTTCCACTCCACCTACCACCCTGGACGTCGAGGGTAAGGTGGGCGGG
Hist1h4k	NM_178211	CCTGCAGGGAAGAGGCGGGAAGTTGCGCCCGCAGCTGTGGGGGAGGGGAAGACTTACCAA
Hist1h2bn	NM_178201	CCTGCAGGGAAGAGGCGGGAAGTTGCGCCCGCAGCTGTGGGGGAGGGGAAGACTTACCAA
Peci	NM_011868	GGAGGCTTAGGCCCAGCAGAAACTGCAGGGCAAGACCGGGAAGGGGCGGAGCCAGAGGTGCGCTC
Arsk	NM_029847	GGTGTCTGCTCCCCGTCCTTTGGGTACTTACGAAGGAGTCACTGGCGACCAGCACCACGTTGGGCGCCTGGTTC
Eef1e1	NM_025380	GGTGTGTCGCCAGAATGAGCAGGGTCCTATTCCGTGTTCCCTCCGCCAGGCGGCTTCCAGGAATCTGTACCGCTCGCCC
Zfp459	NM_177811	AGGAATCTTCAGCATCACAGGACTCCAGTCCAACTTTCGGGAAGATTGACGAGTGTTGACCAAAAGCCGGGTGGGATTTGCTTGTGACGTCGGAGTCACGTGG GTAC
Atg10	NM_025770	GTTGTGTTGGCTGCAAATCTGAAAAGCCTGCCAGTCACTTAACTTCCTTTGTTTCAAGGTCACG
Elovl7	NM_029001	GGGAACCGGCCCCCTACTGCACCCTGGGTTGCTCGGGGCCAGGCTCTACCCGCGGCGAGGGCGGCAGCGGGAGAGGCTGGCATGACACCCCACCCCTCGGCGT

		CA
Arl15	NM_172595	CCCCAGTACTGCACAGTTTGCCTTCTTTAAGGTGTCTGCCCTTCTAATCCGCAGGTCCAGGGCGTTTCAAGGCCACAGGAAGGCGCTCGGGGCAACTGC
Isgf3g	NM_008394	TCCCTCTGGTGGTAACCGCTTGCCCTGCAACTCAGACCACGTGGTTTCTAGGAAATGCACCTCCCCGGA
Zfp219	NM_027248	CTCTAGACATTGCTTTCCAGGAAAAGGTCAGTTTGCCTGCGGGGGGGTGGGGGTGGGGGGGGATATTTTGCAATACT
Spry2	NM_011897	GGGAGGGAAAGGGGAGCGGGAAGTGAGGTGGGAGGGCTATTGGTTTATTCTTTGTCTACTG
Gzmc	NM_010371	GTCCTGCCTTCCCCACTTTTCCCTACTGCCTGCTACAGTATGTGGAAGACCAACTGTGTGCTACA
Sncg	NM_011430	GCCTCCCCACAACCCCCTTCTGTGTCTCCAGGGATTCGGCCTCTGCACATTTCCAGGAGTGGGAGGAGGGCCTGAC
D14Ertd436e	NM_172599	GTTTTCCGGGGTCATTCTGGGAAGGGAGTGTTCCTCACAACCTTACAACCTTCTAGCAGAAGGTCCCCAGCGTGCTACTCTAGCAAGGACTTTCAGCGGTCATCT TTTGTTCTCTAAGGTCACACATTCCCACAGGT
E430028B21Rik	NM_178668	GTGACTGGGCGGTGCCTCCGGGAAACACTAGGCTTTCTGTCATTTCCAGTTGCTCCGGTTATGGCTGTGTGT
Ednrb	NM_007904	GAGCACCCTGCACATGGATTGACGTTGCTCTGCCATATGTGGATGAGATGTGGTGAGCTGCCTGAGATCATGCCTTGACTTGCCCTCAGTGATGAAAGTCAAATC ACCCTTTCCTTCCCGAG
Slc15a1	NM_053079	CACCCTTTAAGGGTTGATGAGTCTTCCACATTCGCTTTGTTTATTCAATCACCTGCCCTTAGGAACTCCCAGCCACCCTG
Tep1	NM_009351	GACTGGTTGGAAGCAGAGAACTGAAAGAAAACCAACACCCTAGTGAGGCCAAGGGGTGAGGCTG
Pnma2	NM_175498	TCCCTCCCTGCCCACGCCCCCCGGCGCCGCCACGCCACCTCCGAGCAAAGTTGGATCTGAGCGGGCGGTCGCTGCGGCAGAGGATGCCAGTTCCCGGCAACGC GGGGCGGGGCGGAGTGGGGCGGGGCAGGAGGAACGCAG
Dcp1a	NM_133761	CAGCGGATGAGATTAGCTGAAGTACCACGCTCGGCAAGGCTGGGCCGGTAGGCGGAAGTGGAGGGCTGA
C130032J12Rik	NM_178684	GAGCGGGCGACCCTCCCCCACATCCGCCTGCGTGACCGGCTGGGGGCTGGTTCGCTCCGGTTGATCCCGGATTCCCTTTGTTCCCTT
C1qtnf9	NM_183175	CCACTGAGGGTGAGCTCCTGCCCTTCAGAACAGAACAGTATCCTTATGCTCTTGAAACCGCTGCACGTTTGCCTGACCGTTAAGACAC
Clybl	NM_029556	AGGCGGGGCGAGGGCGGGGCGTAGGCGGGCCAGTCGGGAAGATGGCGTTGTGCGTGCTGCGGAACACGG
Vps28	NM_025842	GGTGCTCGCTTTTTCCGCCTGCGCCTGCCGGGAAACCCGATCGGCAGCTCGGCACTCATCC

Naprt1	NM_172607	TCCACCCCCTACGGCACTGGTCCAGAGAACCAACTCACACGAAAGGAGGGCCTTTCCCGGCAG
Pabpc1	NM_008774	GGAGAGGTGGCGGCGGCTGCCGCTGCTGCGGGCGTGGGGGTTGGGCCCGAGGCGGGGCGGGGCTTC
Oplah	NM_153122	CTTGCTTGTGTCCCGCTGCGCTTCCCGAAGGCCCTGCCCCCACCTCGGGGTGGGGCCTGGAGAA
Mtmr12	NM_172958	ACTAGGAGATGCCCCCACCCACCCTGTTAATCCTTCCTGGAAATGCTCTTGTAGAACTGCCCATA
Sh3bp1	NM_009164	GTCCTGCCTCAGCCCCACCCCAGGGTTCCTGGAAAGGCCTTTTGAACTGTGGAAGTGGGGGTGGGCAAGG
Ptk9	NM_008971	TGTCGTCACAAGCCCCGCCCCTTAAGCCATTGGCCATGCCCTTTCCCGGCAATCACCCCC
Cbx5	NM_007626	GGGAAGTGGAGTTGGGAAAGGAGTAAAGACGGAGAAGGGGGAGGGGAGGGAGGGAGGACAGG
Amacr	NM_008537	CCAACTGATCCAATGCTAGGGTGGGCAGGAAGTGCGCAAGCGCGCAGCCTTCCCGGACGGGGC
AK162044	NM_177597	CAGGGATGCCTACCTCGCCCTCATCCTTACTCCCACGCGGGTCTCTGCTTGGCCCACCGCGC
Myc	NM_010849	AGGAAGCATCTTCCCAGAACCTGGAAACCCTGCAGCCCTGCCCCCATCCGACCTCCGCCCTCGTTGGCTTCGCAACGCTG
1700088E04Rik	NM_138581	AGAAACTCCAAGGAGCTAGAGTGTCAGCGTCCTCACTAATCGCTCCTTGTTACCATGGTAACTAGCGACACGCCTCCACGCACGCGCATTAGCGT
Polr2f	NM_027231	AGAAACTCCAAGGAGCTAGAGTGTCAGCGTCCTCACTAATCGCTCCTTGTTACCATGGTAACTAGCGACACGCCTCCACGCACGCGCATTAGCGT
Ropn1l	NM_145852	CATGGTGTCGGGCAGCGGCATCGTTCTGGGGAGACCGCGGCGGCCTGGGACGTTGTGCTGCGGGGAGGAGAGGCAGGGT
Myc	NM_010849	CCTCCCCCTTTCTGGGAAGTCCGGGTTTTCCCCAACCCCCCAATTCATGGCATATTCTCGCGTCTAGCGCCTTGATTTTCCCCACCCCA
Apobec3	NM_030255	GGGACAAAGAGGTGGGCAGGAGCCTTCTGGGCTTGATCGCCTAGAGCCCTCCCATTCCGGGAGCTG
Mettl7a	NM_027334	CCCGTGACCTGGATGACCCTGCACTGAGCTGCACTACAACACCTGAATTCCAGCGCTCCTACAGTCATGTGGGAAGCAAAGACAGAACTGCACGAAAGCCAGT AAGTCAGCTGGT
Cox6c	NM_053071	AAGACATCCTAGTTCGCCGCCCCCACGAGAAGCCGAGAAACCCGCAGTTGTCAAACCCCAGAATCTCCTCTCTCTCTCCACCATTCTCACCTCTACACCAACGTC CTTCCCGGTTCTCGGAGAAACGGAGA
Zfp406	NM_198644	CAGGCGCGCAGGGCGCTCCCCTCGCTTGCTCTCCACCGCCTCCCTTTTTTTTCTTTCCTGCGGCAGGATCAAGCCAGCTTCCAAACG
Jrk	NM_008415	TGCGGACCCCGTGCCGCCGCTACCGGCCGGAAACGCCAGCCACGCAGCTCTCCACCGGCCCGCTCTTCTCGGCCTTCCCCGACCCACTTCC
4933427E11Rik	NM_025756	TGCGGACCCCGTGCCGCCGCTACCGGCCGGAAACGCCAGCCACGCAGCTCTCCACCGGCCCGCTCTTCTCGGCCTTCCCCGACCCACTTCC

Aaas	NM_153416	CGGGTTTCGGGACGGACTTCCGGGACTTCCTGGCGCGCCCCCTTGCGTACGGAGCGGGCACGACGCTGACACGTCCGAGGACCGACCTGTGGCGTAAGGA
Chrd	NM_009893	CCCTTTCCCTGCTCTGTCCTGCTCCTCCCATTCTGGGAAGGCTCCACTCTTCTTGACAAAGGTCAGTGCCTGTA
Prodh	NM_011172	TGGGTTAGCCACGCCCTGGGGCCACGCCCCGATCACGCCCCATTCCCGGAAGCCCCACCT
Liph	NM_153404	GAACCTGCCCTGGAATGTGCCGCTCCCTGCTGGGTCAGTGTACCCACCTGATAAGTATTTGTTGA
Bcl6	NM_009744	GAACCCCAGTTCTAAGAATCAACAGAGCTCAATTCTCGGAATTTGAGCTGCGGGCTTTACCATTGC
5730409G15Rik	NM_027446	AGGCGGACCGGGCCGGGTTGGGCCTGACTGGGGAGCGTTTTAAACAGTTCCTGGAGCTGGAGCTGGCAACTTGGAGGCGAAGCCT
Lpp	NM_178665	TGCCTAGCTCTAACTTGGGTCTAAGCTCACAGTTAGAGGCCGGGTCGTGCCTGGAGGCGGAAA
Coro7	NM_030205	AAGATGTAATTTTCAGGGTCAAGGGAGGGCATCCACACGGGGTATACCGGAATCTGATACTG
Dnaja3	NM_023646	AAGATGTAATTTTCAGGGTCAAGGGAGGGCATCCACACGGGGTATACCGGAATCTGATACTG
Fyttd1	NM_027226	CAGGGCCAGGCTAGTCGTTCAGACTCGGTTTCGGAGGCGCTGCCAATGGTGTGGACCGTCGA
Rrn3	NM_001039521	TGGTGTAAGACTGTGGGCCCCAGAGTGCTCTTGGAATAAAAGTTCTCTTGCGGTTTGCATCAAGACCATTTCTTATGAGTGATTTGGGGTGTTGCCTCTCCTGAGT CAGAACGT
Pros1	NM_011173	CCCTCCCCACCCCACCCCCGCCCCCCAGCTGCGAAGAGCTTCCAGGAAATGTCCCAGTCACCGCTTCCCCTCCCGGGCTGGGGGCTGGGAG
Ube2l3	NM_009456	GGTAGGCCAGGCGGCTAGAACCCCCTCACTTCCTGGAGCGGGGGAGGGGCTAACCGGGAA
Eif2b5	NM_172265	ACCTCCCAGTAGCACCTACTGCGGTCAGCCACGCCCTGGTCTTGCACGGGGTGGGTGGGGT
Trap1	NM_026508	TGCCTGTGCGAGAACGGGAACGCAGCAAACCGCAGCGCGGGGGCCCTGAAGACTGTACTTTTTGA
Dnajb11	NM_026400	CACGTACTTTGACACCTCCTGGGGTTGCACTTCGGTGAGCGCTCGCAAGTCACGTGTGCACA
Ifitm7	NM_028968	CCCCACATACAGAAATGGGGGAGTGTCTGTGTGCTCTTTCAGCCTTGAGGTTCAGAGCATAGCTGA
Morc1	NM_010816	ATCTCCATGAGAAGAGCCACATAGCAGGCTGAGCTTCAGCGCTGAGAACACCAGGGGGCGAAGGCGTCTGTTGAGCTCCCACTCTGCAAGTGAGCCAGGCCGT ACGCGTGGACGTAGGCGTGGGCGT
Tfg	NM_019678	GCCCGGCTTCCTCACTGGATGACCGAGTGTAGGAAGTGCGAGCCGAGGCGGACACGCATG
Pou5f1	NM_013633	CCTCCCCCCAGGGAGGTTGAGAGTTCTGGGCAGACGGCAGATGCATAACAAAGGTGCATG
E130307M08Rik	NM_026530	CCCAGGGCCTTGGGCGCCTAGTAACACCGCCCCTTCCCGCTCGCTGATTACACGTGAACGC
AW049765	NM_145934	CCCAGGGCCTTGGGCGCCTAGTAACACCGCCCCTTCCCGCTCGCTGATTACACGTGAACGC

Zfp13	NM_011747	GGAAGCCCCTTTATCCCCTTACCTGAAAGGGATGGGTCAGGCAGGGAACTTCCTGTTCCATTTCCCGG
Zfp206	NM_001033425	GGAAGCCCCTTTATCCCCTTACCTGAAAGGGATGGGTCAGGCAGGGAACTTCCTGTTCCATTTCCCGG
Ly6g6d	NM_033478	CCTCTAGGGCAGGGGAGGGTTCAGAGTGTGACCTTGAGAGGCTCTGACGGGAAACAGACAAAACAAAA
Ly6g6e	NM_027366	CCTCTAGGGCAGGGGAGGGTTCAGAGTGTGACCTTGAGAGGCTCTGACGGGAAACAGACAAAACAAAA
Stk38	NM_134115	TTTTTCTTTTGAAATAATTATAGACTCGCCAGAAGTTACAAAACGAAAACCTGGAAGTTCCGTAAAAGCCAACACCCACCCA
Lama1	NM_008480	GACAATAGAGACACTCTGATATATCTGGGAAAGCATTCTCTAAGAGTTACTGTCAAAGACAAGGTCTAAATG
Tmem8	NM_021793	ACTGTGTGCTGACTACTAGGCCAACACAATACAGTACGTTTTTGCATTTGCTGTTCCCTAATTTCCTGGAAGGGTTC
LOC547127	NM_001045538	TCTTGGTGTCTTGCGCACAGGCGTGCTCACACAGACACTGTCTGACCCCATCCCCACCCC
Pde9a	NM_008804	GAATGCACAGGCTGCTTGGGGAAGGAACTTTCCCCACTTCCGATTCAAATTAAGTGAGGGTGGCCTTT
Pim1	NM_008842	CCCGCCACACCAACGCCCTTCCATTTCTCACAACTTGCTACGCTCCAGCTCCAATCCTCGACCCCCCTCCACAACCCAAATCTCCGGGAAACACAGAGCCGGCAGAG
Stk19	NM_019442	GCCAGGAGCCGTCTCTTGCGATTCATAACCCGGATTTCAGGATTCCGGGAAAGGAGTCCCGAAGACCG
Dom3z	NM_033613	GCCAGGAGCCGTCTCTTGCGATTCATAACCCGGATTTCAGGATTCCGGGAAAGGAGTCCCGAAGACCG
Flot1	NM_008027	GGCATCCCCCTGGGCTGCCGAGAACTGTAGTGCTGCGGACCCGAGCGGAGCCTGCCGGGAAAGGGGCGGACCGAGGG
Nrtn	NM_008738	GCCCCTGCCACACCTGGTTGCCGAGAAACCAGGGTTGAGGCTCCTCCCTCTTTGACATCCTTTGCCAAATCTTCAGCTCC
6330416L07Rik	NM_176962	TGGAGCTCCGGGCGACACTATCTCCCTCAGGAGACAGAGGTGTCAACCATGAGGCTCAGAAG
Pknox1	NM_016670	CATCCTGGGAACCCCTCCATACCTTCCCTTTCTCCTCATGTTCCTATCCTGTGCTGCCTCTGGTGTCTCTCCTACT
Pou5f1	NM_013633	ACACCGGAGTCCCTGGAGGAAGGGAAGCAGGGTATCTCCATCTGAGGCTCTGTCTTTGAG
Riok2	NM_025934	TCCTCCTAACCCGTCCTGGGATCAGCGTCCCAGGCCAGCGGGGCAGCTCCTAACCCGGCCCTCCTCCGCCGGACAGGGGTGG
BC049807	NM_001002008	AGAACCGGAGCCAGGCGGCCCTGGAACCGCCCAGGTCAGAGGAGCTGTGGGGTTGGAAAAGGGCGGGAAGGGAACAGGGTGTGATCGGTTTG
Hcfc1r1	NM_181821	GTGGTAGGAATCAGGGTTGAGGGTACCTGAAGATGGCAATCTGCCCGTAATTGTTGCCAGCTGCCAGAAACTTCCCGCAGGGGGAGACACT
Rps10	NM_025963	TCTGCTCTCCTTTGTGACCTTTGCCGGCTCCATCTGAATCTTTCCCGACAACTGTGCTCCCCAACCCCCACGCTTGGCTTCATCTCAGT

Cdkn1a	NM_007669	TCAGTGCAGGGTGGTGGAGACCTGATGATACCCAACTACCAGCTGTGGGGTGAGGAGGAGC
Rab5a	NM_025887	CTGAAGTGGTGTGAGAGGCCTGTGAGTGAACCATCAGACCGAAAATCGCCGCAATGGGAAG
Zfp13	NM_011747	CAGTGCAGCAGCGCTCCTTGTGTGGTGCTGCTCTGGGCTGCAGTTACTGGAATGGAAAGTTCTGCCTAAGGCTCTGGTCCCATCCCCCTATCGGAACTCCCACTG TTTGGGGGGTTGG
Pnpla1	NM_001034885	GGCAAGTCTATAGGGTATTTTCTTGATTAATGATTGATGGAAGAAGGCTCAGCCCACTGTGGGCGGTGCCATTCCT
Rps28	NM_016844	TGAATGGCATGGACCCCAAGTTGGCTTGAAGGCTGTAAGGGAAAGGAGTTGCACGGATTTACT
H2-T24	NM_008207	GGTGGGGAAGTGATGAAGCTGGGAAGTCGAAACCGAAACTGTAGCACTTTTCTACTGCTG
Calm2	NM_007589	CTTTCCCCGACCCGCGGGGCGCCGGCCGCTCAGCTACGCACTCACCATGCTGCAAGGGCTACGGGTTTCCGAGACGCGACCAGACAACCGCTCGAC
1600002H07Rik	NM_028056	CATTTTTGCCGCCGCCAAGGAGTCCCGCGCCTTCCGGGCAGAAGGCCGCCCACTTCCGGGTAGGCTACCCAATCTTGAGCACAGCTTGC
U2af1	NM_024187	CTTAGGCTGTGGACAGCCGCTCCGCGCGTGCTCAGCACCGCTGTTTCCCGCTCCCGCCGCTGGGACCGCGCCCTCTCCCCTTGCTG
Pou5f1	NM_013633	GGGCAGGCATTATAGGTGTGGCATTCCGCATCTGGCTTCCCAGGATACCTTTTCATGCTGGTGGACCATCTCTGGCTGGGGACGTG
Tubb5	NM_011655	CTTGGAATGACTTCTATGATAGAGCCCTTCTAGCCGCCGCCGGTAACTGGCGCCAGACTCTCTGGTAGGCGTTTGGAAATCTGCGCGGGAAGTAGTCCGTGGATG ACGTAGCGACGGAGG
Gna-rs1	NM_008136	TTAGGGGTCCATCCCTTCTCCAGGGCTTAAAACATGAATCAGGGCCCAATTTCTCCTCACCTGACTTGGGATCACCAGGCTTCCCGTTAGCCATGGGGGGAGGGC CCAGAAGGCTGGGTGGTCCTAAGAGCAGACGTGAGAAAACAGCCATCAGA
2410137M14Rik	NM_029747	GAGGTGCCTGTTTAACATACCAAAGTGGAGTGGCCTCCAGATTCTTCTGTCCTTGAGTACCTACCCTCAACAGGGTATGGCTGAAGTA
Spats1	NM_027649	GGGTTTGAATGTCCTGCCCACTCCTCCTCCTCCTGACTCAGGGCTCCCGAAAGTACTCGCCCCGCCCCGTCCTTGGGTCTCCTTTCCACCTCTGAGTCCCACCCT AC
Spats1	NM_027649	CTTCCAGCCGCTTGCATGGCACTCATTGTCTTCAAAAGCTCTAGGGAACTCTAAATGATCTTGCCCCTGATCGGAAGAGGAACACTTTCCTGGCA
E130307M08Rik	NM_026530	TGAGGTTCTGCCCTGACCACGCCCCCAGGCCCCCCACTAAGAACGGTCTTGAGAGGGCCTGGGCGCCTGGCTTGTGGTGGTAG
AW049765	NM_145934	TGAGGTTCTGCCCTGACCACGCCCCCAGGCCCCCCACTAAGAACGGTCTTGAGAGGGCCTGGGCGCCTGGCTTGTGGTGGTAG
Fbxo15	NM_015798	GCTAAAATGGCACGCAGCCACCGCCCTTAGTTCCCAGATGTGCTTTATCATAACAATGGAATTCC

Eif1a	NM_010120	GGGCCAGTGCCGGGAAGTCGACGGAAAGGGGCGTGGCCGGCCGTTGCCTAGGAAGGGCGCGT
Osbpl1a	NM_207530	CTGTCCGCCTCCGCCGTGGGGCGGAGCCTCTCCCTCCTTTCTCGGAAAGCAGCAGCGGACTAG
Wdr55	NM_026464	GTCTTAGAGGAAGCTGCACGCTGCGCTCGCTTAATTCGCATCAGCTGCGGCAGGCCCGCCTCTTCTGAAATCCTCCCAGTGCACTTCCG
Tcf4	NM_013685	GGAACGGGGCGCCAAGCGCGCTCCGAGAGCCACGACCTGAGTGAGTCCCACCCGCCGGCGGGACCGCCCTGG
Ccdc68	NM_201362	GAGGCTAAAACTTCCAGGCAGTGGAGAGCCAGAGGCTCCTCCCCGAGGTCGGGGACACTGGACTCTGGCCTGGGCG
Slc6a7	NM_201353	CTGCCGTGAGGCCGCTGCGGGCGTGGGGTGCTACGAACGCCCTGCCTCTCCCTGCGCAGCGCCCTAGG
Lman1	NM_027400	GGGGCGTGGACGGGGTCAAGGACGGATACTCGGTATAGTAGAGGTGCGGTGTGTCCAGCTCTGTAGTGCCTTTGGGGGAAGGTGATTAC
5133400G04Rik	NM_029485	CATCACTGTGGACAGCTCCCACAGCTCCTGTGATAGCTGTAGGCTGAGGAGATACCATAGTGCCAC
5133400G04Rik	NM_027733	CATCACTGTGGACAGCTCCCACAGCTCCTGTGATAGCTGTAGGCTGAGGAGATACCATAGTGCCAC
Cxxc1	NM_028868	CTGCGTCGCAACGTCGAAGCAAATGCGGCCATATTGCCCTCTGGCGGCCACCGTACGGAC
Pura	NM_008989	CACTTCCTGTCAATGGTTCCTGGAGCAAAAGCCTGCTCATCTAGCCTCTCTGCTTTATGCTAATTTCTCCCCTCAATCCCCTCTCGGCGGGCGTCTCAAGGCCAAG GTGAGACCCTCCCCGGAGATGAAAGGCTCGCTGC
Napg	NM_028017	AGCCCCGCCCTCTCCGCGCATTCATTCCACCCATTTCCGGGACAGCTTTGCCACAGGAAGTTTGAGTCCTTCGCTTCCCGTAGTCCCCTT
Smad7	NM_001042660	CGGGCGAGCTCTGCTGGTCTAAATCGGGCCACTAACCGGCTCATTCCGCTCCAAACTCGCCCGC
Ier3ip1	NM_025409	CCTAGCTGAGCGTCCGGTGCGTGAGAGACTGTGTCAGGGGAATGAGGCAGAGAAGGATAGAAGACACCG
Ccdc5	NM_146089	AGGTAGGCAGGAGGAGACTCTGATTCCACACTGGATTGGAGTGTAGGCACAGGAGACATCA
Atp5a1	NM_007505	AGGTAGGCAGGAGGAGACTCTGATTCCACACTGGATTGGAGTGTAGGCACAGGAGACATCA
Tut1	NM_197993	GAGCTGGAACTCTCAATCCTACGAACGAGGTTTCTGGAGGAATATGGCAGCAAAGGACACGA
Dak	NM_145496	CTGCCTGCGTACCGGTACTCTCGCTCTCATCCGGGTACTGCGACCTCTGGCGGTTAGGAG
Ddb1	NM_015735	CTGCCTGCGTACCGGTACTCTCGCTCTCATCCGGGTACTGCGACCTCTGGCGGTTAGGAG
Rab3il1	NM_144538	CTTTGCTCACATTGTCTGGGGCCTGCTTGCTCAAGGTCATCCTGTGGAGCGCCTGCAAAAG
Hells	NM_008234	CTCCCCCAGCTACTGCTGGCCTCCTCTTCCAGTAGAGATTTAAATAATAATGAAAGACTTC

Lgals12	NM_019516	GATCTGGGTCCCCAAGGCTAGTGGTGCCCTTCCCCCACCCCACATCAGCCCTGCTGGGAAATGAGGGAGGAGGGTGCACT
Hrasls5	NM_025731	GATCTGGGTCCCCAAGGCTAGTGGTGCCCTTCCCCCACCCCACATCAGCCCTGCTGGGAAATGAGGGAGGAGGGTGCACT
Klf9	NM_010638	CGGGAAAGGGCGAGGTGAGGATCTATCCCAATGTGAGGTCTGACACGTGATCCTCTACCCCCAGCGGGCCATCTGTTCTTTTTCCT
Gldc	NM_138595	TGAGCCAAAGAAACCCTTCCTCCCTTAAGTCGCTTCTATCAGAGCTTCATCACAGCAAGGAGAAAAGAGACAGAGGCAGTGCATAACCTCGACCAGGAGTGGC TCT
1810055G02Rik	NM_028077	CCTCTTCTCCCCGGCTTCTGGGAAAAGCCCTAGACACAGACATAGCCTACAAGTCCCTGG
Efemp2	NM_021474	AGATGGAAAGAGGTTGGGTAAGGGAAAGTTAAGGAAGACCTGGTTATTGCCGCTAGCGGTCTGGGCCAGGCACG
Ppp2r5b	NM_198168	GATTAACAATTTGCATTTGGCTTAGGACCCCAGGAAAGCAGACCTGGCCTCTAGGTTCTCCCAGCATCCCTCAGTTCCTTCCTGGCATACCC
1810013C15Rik	NM_194348	GATTAACAATTTGCATTTGGCTTAGGACCCCAGGAAAGCAGACCTGGCCTCTAGGTTCTCCCAGCATCCCTCAGTTCCTTCCTGGCATACCC
Ubtd1	NM_145500	AGCGCACGCTACTTCCAGGAAGGCAAGAGGCCGGAGCGTAACCCTTGGAACCCAGACCTTCTCACCTAACACTCCGCAAACAACGCGGTCCTGCGGTGCAAGC ACCAGGTTTGGACCAGAACACCCGA
Mms19l	NM_028152	ACGGCCCCTCCCCAGAGGGCGGGGCCTGGGCCGGGGCGCGGCTCCGGGAGGGGAAGTGACTGCC
Ubtd1	NM_145500	ACGGCCCCTCCCCAGAGGGCGGGGCCTGGGCCGGGGCGCGGCTCCGGGAGGGGAAGTGACTGCC
Gm962	NM_001033448	CCCCTCAACTGTAACAGGGTTTAATAATCCTTTCTGGCTACCGAGAGCTGGGACACCAGACC
Nolc1	NM_001039351	TTGGAAATCTACAGTAGGAAGAAGCCCATTCAGCAAAGGTAATGCGCGGGAATTTGCAAGTTTTCCCGCCTCGGACAGGAGCGTGGC
Nolc1	NM_001039352	TTGGAAATCTACAGTAGGAAGAAGCCCATTCAGCAAAGGTAATGCGCGGGAATTTGCAAGTTTTCCCGCCTCGGACAGGAGCGTGGC
Nolc1	NM_001039353	TTGGAAATCTACAGTAGGAAGAAGCCCATTCAGCAAAGGTAATGCGCGGGAATTTGCAAGTTTTCCCGCCTCGGACAGGAGCGTGGC
Nolc1	NM_053086	TTGGAAATCTACAGTAGGAAGAAGCCCATTCAGCAAAGGTAATGCGCGGGAATTTGCAAGTTTTCCCGCCTCGGACAGGAGCGTGGC
Zdhhc6	NM_001033573	GCCGGGGGCCGCGGAACGCCGCCTGGGCACGCGGGGCTCGCTGGCGCGAGGCTGCTGTGGGTGGTGCTG
Zdhhc6	NM_025883	GCCGGGGGCCGCGGAACGCCGCCTGGGCACGCGGGGCTCGCTGGCGCGAGGCTGCTGTGGGTGGTGCTG
Vti1a	NM_016862	GCCGGGGGCCGCGGAACGCCGCCTGGGCACGCGGGGCTCGCTGGCGCGAGGCTGCTGTGGGTGGTGCTG

Ddx3x	NM_010028	CCTTCCCCCTTTCTCCTCCCCGCCCTCCCGCAGTTTTCCCGTCCCTCCCTCCCATTCCCAGCACTAGTCAAGCGAGTTTC
G530011O06Rik	NM_001039559	GGTGTTATTTTGTTCGACTCGGGTACACACTTCTCCTGATAGGGACAAGACCTACCTTTAGAATG
G530011O06Rik	NM_001039559	GAATCTCCGAGGCAACTGTCAACTGACCTGTAATGAAAAGTACGCTCTACATGGAAGTCCACTCGAG

Appendix2

The sequence from chip-clone results

1、

GGTGGGGGGGAGTAGCTGCTGCCAGGTACGTCTCCCTCCAGAAACGACTCTGAGACCAGACAAAATTCGTGCGCTGCTCTCTTCAGGCAGTGGGGTG
AGGACAGCACAAGACTGAGAGGGTGCTTGGAGGCGCCCACACTCCCGGCTCTCCTTGGCCATAACCCCACAAGCAGGGCTCCAGGCAGAGCACGGC
AGTCCCCCTGGGATGAGAGGCAGAGACTGGAGTGAGGGGCTTAGGTGTGCATGTTGCTGGTGGGGAGTGCTTGGGACCTCTGCAAGCCTGTGGCTGA
GGGTGAGGAGGTGCCGGAGGCTCACCAGAGAGCAGTGACTATAGGCCCTGTGCTGTTATCCGTCACTGCATAACCAATCAACACCGATTTAGTAGCTT
TAAAAAACATGCATTTATTCTCAGTTTTGGGGGGTCAGGAGCCCACACACAATTCCGCTGGGTGGCTCGGGGTTTCAAGAGTGCCACCAAGGTGTTGG
TATTTCTTTCTGAAGCTTGAGGTCCTCTTTGAAGGGCACGTGGTTGTTGGCAGAATTCAGTTCCTTGTGGCTGTAGGACTGAGGTCCCTGCTTTCTTGTT
GGCTGGCAGCCTGGGGCCACTCACTGGTTCCCTGATACGAGGCCCCTCCCATGCCCCCCAACACCAAACACACCCAACAC

2、

TTGGTGGTGTCCTAAGATTTGGGATTTACCAAGTACTGAGAATAGGACCTTCAAAAAATATGAAATGCCAAGAGGATCTGTTTTAAAGCAATGGAATTA
TTTGAGAATTTTGAAAAATACAGATTCTCAGGACCCAGTCTGAATGAGAATCTCTGGGGAAGAGGCCAAGGAAACAGAATTCAGGTAAGCACCTTGG
CTGCTTCCAGTGGTTGGCCAATTTGGATATCCCTGTTGTGTCCTAAATTATACCTAATTTGATTAAAGTTTTGGTGGCTGGGAGGTTTGCTAGTTGAAAA
ACACATACAGGAAAAAAAAAAATGTACAGGGGAGTGTATTGCTTTGGTGTAGTCGTTTAAGAGTTGACAAAAACCCAAACACACCCAACACAAA

3、

CGTTTTTGGGTATTAGGGTTTAGCACAAAGATTATAAAACTATAAACACAGCCTAAAATAAAATGATTTTTTGTACAACTCTTTGATAAGTAAGACTAATT
TAATATTGTTGGTTTAATAAAAATAGCTGTATCTTCTGAGTTATTGACAAAATAACCATATATTTAAGGTTCTTAGGTGAACACCTGATATGCACAGGCTAT
AAAAATGGTTAACAGAGAAATAACTGATGACTAGCTTTGTCTAATATCTCAGTTTTAATAAGTAATCTAGGTATAATTATTAAAAATAAATTTTGTAAATGT
AAATGGTATATGTGTTTATAAATAAGCTTTTTATGTAATTTAAAATATTAAAGTTTTATGGTATGTTAAATTAAACAACAGATATTCATTAAATGTCTGGTTC
ATTTCCCAATTTTTCTTTTTTTTTCGGAGACAGGGACCCCTCTCTGTCGCCCAGGCTGGAGTGCAATGGCACAATCACACTCATAGCAGCTTCGACCTC
TCAGGCTCAAACAATCCTCCTGCCTCAGCCACCCATGTAACTGGAACCAAAAACACACACCAAACACACCCAACACAA

4、

GTGTTGTTTAAATCATATTCATCAGACATTAAATGAAAAACACAGCATGTTCCTACTTAAAACCCATTATATATTGTATAGTTTATTTATATATTAAAAAAGG
GACTTTACATTTATATATCAGAAAGCTAATAATAACCATGCAAGGGATGATGACTAAAATGTTCTTAATAATCAAGATTTCTATTCTAATATAGAAACATAT

TTTTATAATGTTTATATGTTAGAACATACAACCCCACACCAAACACACCCAACACAA
5 、
GGTGTGGTTGGTTCACAGCTATTAAATGATGTGTTCAAGATCATTCACTTTGTAAATATACTTGATTCCAAAATCAAGAGAACCTATGCCTTTAAACCAG
AGCTTCTCAAACGTCTCCTTGAAAGCTTTCCATGTTGGAGAAGCACAGATGTCTATAGCTGAGGTTCTGCAGCTACAACCAAACACACCCAACACAA
6、
TGTTGGTTTGTACTGATGAGGGAGCTGGAAAAAGAGAATTTAAGCCTGAAAAGTGTGGAGGGGGCTTTAATGAGGGAGAGGAGGTACAACCTCTCAA
TAGGAGGAAGAACTAGACGAGAGAATCTTACTTCTCTTGCGAGCTCACTTCTCATAGTCTCCTCAGGTTCTGAGAGGACTTAGGGTTGGGATCCAAAA
CAACCCAAACACACCCAACACAA
7 、
TTGGTTTTGGTTTTTTGGACATGAAGTCCTTGCCCACGCCTATGTCCTGAATGGTAATGCCTAGGTTTTCTTCTAGGGTTTTTGTGGTTTAATTAGATCCC
ATTTGTCAATTTTGGCTTTTGTTGCCATTGCTTTTGGTGTTTTGGACATGAAGTCCTTGCCCACACCCCCCCAAACACACCCAACACAA
9 、
GGGGGGGCTTGGCAGGCAGATCACCTGAGGTCAGGAGTTCAAGACCAGCCTGGCCAACATGGTGAAACCCCGTCTCTACTAAAAAATACAAAAATTA
GCCAGGCATGGTGGCGCACGCCTGTAATCCCAGCTACTCGGGAGGCTGAGGCAGGAGAATCGTTTGAACCTGGGAGGCAGAGGTTGCAGTGAGCAG
AGATTGCGCCATTGCACTCCAGCCTGGGTGACAGAGTGAAACTCCTTCTCAAAAAAAAAAAAAAATTACAAAATATAAATGAAAGAAATCAGAGGAGA
TCCAAATAAATAGAGAGACATATCATGTTCATGGATTGGAAGACGACACGGTAAAAAGGAGTGGAGTGCAGTGGCTCATGCGTGTAATCCCAGCACTT
TGGGAGGTCAAGGTGGGAGATGGCTTGAGGCCAGGAATTCAAGATCAGCCTGGGCAACATGGTGAAACCCTCTCTCTACCAAAAATACAAAAATTAG
TCAGACGTGATGGTGTGCACCTATAGTCCCAACTAGTCAGGAGGCTGATGTAGGAGGATTGCTTGAGCCCAGGAGGAGATCAAGGCTGTAAGTGAGTT
GTGATTGCACCAAACACACCCAACACAA
10 、
GGGGGGTGGGGTACCAAAGATCACCGGAGATAAGGATTTCAACCCAGCCTGAAAGTGATGGAGATACCCAATCTCAACTCCTCTGGACTCAATTAAGA
CATAGTGGTGGCGCGTGCTGGCTTCCCAGCTACTCGGGAGGCTGAGGGAGAGAATCGTTTGAACCTGGGAGGCAGAGGTTGCTCTGAGCTAAGATTG
CGCCATTGCACTCCACCCTGTGTGACCGAATGAAACTCCTTCTCAAAAAAAAAAAAAAATTACAAAATATAAATGAAAGAAATCACAGGAGATCCAAAT
AAATAGAGAGACATATCATGTTCATGGATTGGAAGACTACACGTTAAAAAGGAGTGGAGTGCAGTGGCTCATGCGTGTAATCCCAGCACTTTGGGAGG
TCAAGGTGGGAGATGGCTTGAGGCCAGGAATTCAAGATCAGCCTGGGCAACATGGTGAAACCCTCTCTCTACCACAAATACAAAAATTAGTCAGACGT
GATGGTGTGCACCTATAGTCCCAACTAGTCAGGAGGCTGATGTAGGAGGATTGCTTGAGCCCAGGAGGAGATCAAGGCTGTAAGTGAGTTGTGATTGC

ACCAAACACACCCAACACA
11 、
GTTGGTTTGTAATGTGCCAATTAGTACATAATTTGAATTTAATTGCTCTTCCTGATCATAGAATAGAGTAGACGGCTAGGCTTGATAAGGTTAGTAAAAAT
AGGAGGCCTATTTTAAAATTAATTAGAGGGTCTGGTATAGGGATGGGGGTTCACAACACCCCCAAACACACCCAACACAA
12 、
TGGTGTTGGTCATACCAGTTAAAACATGGGGGAGGCTGGGCGTGGTGTTTCATGCCTGTAATCCCAGGACTTTGAGAGGCCAAGATGGCCAGATCATT
TGAGGCCAAGAGTTGGAGACCAGCCTGGCCAACATTGCAAAACCCTAAAAATACAAAAATTACTAAAAGTAAAAAATTAGCTGTGTGTTTGGTGTTGT
TTGGCAGGATGGTCTCGATCTCCTGACCTCGTGATCTGCCCGCCTCAGCCTCCCAAAGTGCTTGGATTACAGGCATGAGCCACCATGTGTAGCCAGTAC
TTCTTTTTTTATGGCCAAATAATATTCCACTGTGTGGATATACCATACTTTATCCATTCATCATCATCAGGTAATGGATATGTTTTGATTCTCTGCCCTACTTT
ATTTTTGAGACAGAGTATCACTCTGTTGCCCAGATTGTACTACTGCAGCCTCCAAATCCTGGGTTCTCAGCCTCCTGAATAGTTGGGATTACAGATGTGA
ACCAACCAACCAAACACACCCAACACAA
13 、
GTTGGTTTGTAATGTGCCAATTAGTACATAATTTGAATTTAATTGCTCTTCCTGATCATAGAATAGAGTAGACGGCTAGGCTTGATAAGGTTAGTAAAAAT
AGGAGGCCTATTTTAAAATTAATTAGAGGGTCTGGTATAGGGATGGGGGTTCACAACACCCCCAAACACACCCAACACAA
14 、
TTGGTGTGTTCCAATTAGGAAAAACGGGGAAAAAAGAAAAAAATTGAAAACATTATCTTGGAGACCTGTAGCCAGAAAAATCAGAATTCAATCCAAA
CTGTAGAAAACCCAAACACACCCAACACAA
15 、
TTTTTTTTTTTTTGTGAGACACAGACTCTCTTTTTGTCACCCAGGGTGGAGTACAGTGGCACAATCTCCGCTCAGTGCAACCTCCATGTGCCGGGCTCG
TGCATGTGACGTGTCTCCCCCTCCAGAGTATGGGGGATTATCACCACCCCCCCCAAACACCCCCAACACATATCTCTAGAAGATCCCGGGGCACCGATC
TCAAATTCAAAATCGTGGATATATGTGTTTCGTGTGAGAAAGTGTTATCCTCTCACTATTCCACACAATATAAGACCCAGAAGCATAAAGTGTAAACCGT
GGGGTGCCTAAAGAGAGATCTAACTCACATTATGTGCGTTGCTCTCAGTGCCCTTTTTCCTCTCGAAAAACCTGGCGTGCCCTCTGCTTTAGAGAATCG
CACAACGCGCGGAGAGAGGCGGTGTGCATAGTGGGCTCTCTTCCTCTTCCTCTCTCAGAGACTCTGTGCTCTCTCTCTTTCCTGTGCCGAGAGCGATAT
CATCTCTCTCAAGGGGGGATATAGGGATATCCACAAAATCGGGGTATAACACGAGAAAAACGTGAGAGCAAGGGGCCAACAAAGGCCCAAGACCCAT
AAAAACGCCGCTGCTCTCGCGTTTCTCTAGACGCTGCGCCCCTCAGAAGAATCTCACAAATATCGACTCTCGACTGAGATGTGGAGAAACCCCACAAG
TATATAAATATACGACGTTTTCCCTGTGAAATCTCTCTCGTGCGCTCTCGTGTCCACACTGTGCCGTTCAGCATACACGTGCCCCTCTCTCTCTTTCGAAA

AGCGTGGCTCTCTCTCATATCTCACGCTGTATGTATCACAGCTCTGTATATCTCGCGCTCTAAACGCTGGTGTGTGTGCGCACCCCCCCGTTCCCCCAAA
CGCTGCGCCCTTCCGGAAAATAGCGTGAGAGTACACCGGGTGAGACACACTTATCCCTCTGGCAGCCACTGTGTACAGATAGAGAGCGGT

16、
GGTTTGGGTTACTGTGTTCACTGTCGTATCCCAGTGCAAAGAACACAGAAGGCACTCAAGATATAGCTACTGAATGCATGAATGGTGTCTCACAACCCT
GTGAGGCTGGCATTATTGACATCCCAGGTTACAGATGAGGAAACCAGGGCTCAAGGAGGTTAGGTGACTTGCCTAAGGCCTCACAGCCCTGGACACTC
CCATCACACTGGCCTTCTGCTCCCCAAACACACCCAACACAA

17、
TGGACACCGCCAAAGTCCGGCTGCAGGTAGAGGGCGAGGCGGGGATGGGGGTGCATCTCAGGGCAGAAAGGGGCACCTGTGGGGGACACCCCTTTG
ATTCAGGCTTTGGTTTAACCGCCGTTTCTCAGGTAGGTACAGCAGGTGGCCGCCTGAAACACCAGTTTGACGTTGTCGCGGAACTGGATGCATTAGGG
TGGGAAGCTGGAACTTTTCCAGGGTTAACTGCAAAATCTCAAGGACCGCGGGGATATGAATTGACCATGATTATTGTCAGACTCCAAAAGTTAGGGGG
CACCCAGTGTTCTCAGAGCAAAGACCGTCAGCCCCTCTGGAGCTGACCAGCCAAGCTCTGTCTCCTTCCATCTTTGATTCAATGGAGGGGAGGGGGGT
GAATCTTCCCCTCTTGTACCGTTCTGTGAATTTATGGATCTGATGGATGGAATTTTATGAAGATCCGTCGGAAATCCGAAGATAAGGCGCGCAGTCGGAT
TTTGCTCAAGATTGTTCTAGGATGGGGCCTAATTTTGTAACTTACCTCCCGTGGATGAAGGAACTGTTTCTGATACCGGTAGATAATTTTGTAGCCAGAT
CCATCGAGCACGTAAACACCGGCTACCAAAAAACTTGATAGATAACAACTTGTTCTGGGGGTTAACGTGAACCGATCGTATATGGATAGAATTCGAAAT
ATGTATAGATAATTGATACTTTGAATCTTCCACCATATCTGGGGATTTTCAAGAAGGAACAACCTTCTTACATCCTGTGCGTCAATCCAGGTCTTTCCATC
ACGACGTGATCTGGCTGTCTTTATAGCGAATGTTTAACGATACGCCCTATGCTTAGCCTGTCCTTGCGAAGTTAAGCAGTTTTAGACGAGAGATGATATT
AGCAGGTTCCGAT

18、
GTTTGGTGTGCTCCTTGAAATGTCTTCACCCCTACACCTTGCTTCTAAAGGTCCTATCCCTTCCCTGAAGCCTGCCTAATCTATTCTAGATAGAGCATCCT
CACCCTCTCAAACATGTCTGAACACACACCCCCACCAAACCCACCCTACACAA

19、TGGTGTGTTGAGCTGTGCCATTGAAAGAGTCTTGAACTCAGGCCGGCCTGGAGGTGGAGGTGGAGCTGTGCCAAACACACCCAACACAA

20、
GGGGTGGTGGGGACCCAGGAAGGCCTGAGGTGAGCTATTTACCAGCCAGCGAAGGACTACACTTGACCCTAGAAGGACAGGGGTTTGAATCGTGTGG
TCCACTTATATGTGGAC

21
GGTTTGTGGGTGGGGACCACAACGCAAAAAGAAAGCAGGAATAGACTGGAAATAAATTAATCACCATGCGCTGTGCCCCCCTTTTATGAAATGGGTTC

ATAACTAATAAAACCGGGCCGATCACCCTTCCCAATAGTTTCGCTTCAAGAAAGCATTATGGCGCGAGATGTAGTTTTAGCTAACATCTAGTCTGCTTCC
AGTGTTTCACCCATTGTGGTGCTTTCTCAGAGTCATCTGCTCGCCTATCTCAATTAAAAGCCAGGCCGCTGAGAGGCTTGCTACATCACAAATTTTTAAT
TGAAAAAACAAAGGTGTTTTTAAAGGTATACACATTTAAAGTTAATAACATAGGTGCCAAATTGGATTCAACCTCCACACCAGTTTTTCGAATACCCCC
GGGACAACACGGCCTCCAGACACACCCATCATAATTCGTTAAAGGATTAATTTTTACCGTTCCTTAGACCTCACGTGGAAACATTCCGGTAACTTATCGC
GGATCCCCTGGTGGTTTTATTTCTGAGCACATTCGAATAAGTATCCTCTCCTGAGAAACCAGCTGTGACTAATGAAACCAACCAATTCCAATAAGAAGT
GTATCATTCATCAACCCTTCCGAGTCGGGATAACTGGCGTGACTGCTGCATTTTGAATCCTCTAACTCGCTGCGAGAGGCCGTTTGCTGTAGCGAGCAC
TTCAGCTCATCTCTTGGCTGACTACTGCGCTCCATCAATCTGGGTCGCAGAAGGAAGAACCTCTCACCAAGGTGCGACCACAGGATCCACACCCTTAA
GGCCACCTTTCAAGATATCACCTGAGGCAAGGCCCCCTAAAGGCTTACACAAGAAAAGCCGCGTCGGAGGCGCAATCCCTATGCGCCAACCACTACA
AGAGTTCTCAACAGTGACCTCAAAGTTTAAGTGGGGAGACGCGCTGGGACTTAAGGATCCCTGGCTTCTTCCTCGATAGATGCAACGAGGGCTTCTGT
TTCTACCTGCAACGATCCGGATACC

22

GTTGTTTGGGGTATATCTTATCTGAAAAATTATTCCTGGAAAAGATTCTACTCTTAAATTATCTCCACGTTCAGTGCCACCACCATATTATAGGTGTTCCTC
ATTTATAAAAGATGGATGTCTAGTATTTTTTATTGATTAGAATTAAGAAATCATTTTTATACATTTTAATTGTTTAAAGAACATATAAAATCAATGAAAAATA
TATCCCAAGGAAGTGCTTCGAGACAGGCATGCCCATGCCTATATAGACACAGAGAAACTTAACTCTGTGTGATCCTAGAACACAAATTTTTTATTGAGA
ATCCTTTACAGTTTTTAAAGTTATACACATTTAAAGTTAATAATATTTGTGCCAAATTAGCCTCTGCCTCCATTCAAGTTGCTATAATGAAATATTTGTAAC
ACCACCACCAAACACACCCAACACAA

23、

TGTGGGTGGTACACCTGAGGTCAGGAGTTCGAGACCAGCCTGGTCAATATGGTGAAACCCTATCTCTACCAAAAATACAAAAATTAGCCGGATGTGGT
GGTGCATCTGTAGTCCCAGCTACTCGGGAGGCTGAGGCACAAGAATCGCTCGAACCCAGGAGGCAGAAGTTGCAGCTAGCCGAGATCACACCAGCCT
GGGTGACAGAGCAAGACTCCATCTCAAAAAATAAATTTAAAAACAACAAAACCAAACACACCCAACACAA

24 、

TGTTGGTTTGTACTGATGAGGGAGCTGGAAAAAGAGAATTTAAGCCTGAAAAGTGTGGAGGGGGCTTTAATGAGGGAGAGGAGGTACAACCTCTCAA
TAGGAGGAAGAACTAGACGAGAGAATCTTACTTCTCTTGCGAGCTCACTTCTCATAGTCTCCTCAGGTTCTGAGAGGACTTAGGGTTGGGATCCAAAA
CAACCCAAACACACCCAACACAA

25 、

GTACAGAAACAGTGAGATTGATTACAGCTCAGCATTTGCCTTATTTGAATGGAGTTTGAAGATTCAGCAGTCAATGAGTGGTTAAATTATGGCTGCTGG

GATTGGCCAACACTCAGCTATTATTACAGACGCATACTACTAAGTTAGGTTTTTGTCTGACTATTAAACTAGGTTACAGTTCATCCAAAAGGACTCAAAT
ATAGAAGTACAGAGTCCTTCTCAGACCATATTTAGTTTGCTTTAACAACTATAAATGGATTTCACCAAAAGATTGACAGTAACTAAATCTGGGCAGTAAG
ATACTGAGTAACTTGAAATTAGCTTTTTTTCTCTTTTTGCTTTTCCATATTTTCCAATTTTTCTGCAAAGAATGTGGATTGCTTATAAAACAAACACACAC
AAATAGTTAAGAATTTAAAATAAGAAAATTTAAAAGTAAAATTAGCTATCATTCATTTGAATACCAACCCACCCCAAACACACCCAACACAA

26

GTTGGGGTTTTTGAAGCTGTTGACCTTTAGATGGGGTTTTTGTGGGGACTTTTTTGTTGATGTTTTTGTTGTTGCTTGCTGTTGCATTCCCCTTGCCCTGT
GTGGCTCCAAGATGGGCTGTTGCACCACCCTGTCTTTCCTCACTCTCCATGGGTCATGACAAGTGCCCACTCAGCCCCAAAGAGAGAGCCTGGATACT
TCAGTTGCCAGTGCAGGATTCACTTGCCATTTTTGTTATTCTCGGTGGGAGCCTCTGAGCACAGCTGTTTCTAGTCCAATTTTACTTTTTAAAATTGACA
AATAATAATTGCACAGATTTATGTGGTATGTAGTGATGTTTTGATACATATAATGTTCAGTGATCAGATCTGGGTAATCAGCACATCCATCATCTCAAACAA
CAAACCAAACACACCCAACACAA

27

GTTGGGGGGGACTACCAAATGATTCACCCCTACACCTGGCTGGAGACTTTCTTGATCTTGTTTTTGTTGTGCCTTGCTGCTGCTTTATCCAGCAGATTCA
CCCTTACTAGATGGGAAGGTTGCACCCCCCCGTCAACGTCCTCTCCATGGATCTGACAGATGCCCACTTCCCCCAACGAAAGAGCATCATACTTCTGTT
TCCAGCGCGTGATTCACTGGCCATTTTTGTAACTCTTTTGGGAGCCTTTGACCAAAGGCGTTTCTTTCCAACTTTTAGTCTTTAAAGTTGAATGTAAATA
ATTGTCAGAACAAGGTTGATGTCCTGATGTTTTGTTCAAATACTGTTCAGCGACCAGATCTAGGTAATCAGCCCATCCATGGGCTCAAACAACATACGC
GGATCGAGCTCACATTTCTGGTAGAGGATCCCCGATCCACCGAGCTCGAATGCCTAATCATGGACATAGCTGTTACCTGCCGAAATTGAAAAGCGATAA
CACTTCCAAACAACATGATAGTCGGATTCATCAATAGTAAACCCTGCGGTGACTAATGTGAGAAATAACTAACATTAATTGAGTTGCGGGCAATGCCCA
CATTACCTCAGGATACCTGGCGTGTCAGCTT

28 、

GTGGTGGGGACCCAGGAAGGGCTGAGGTGAGCTATTTACCAGCCAGCGGAGGACTACACTTGACCCTTGTAGGACAGGGGTTTGAATAGTGGGGTCA
AATTTTATGAGGACTTTCTCCTGCCTGTGCCACTCC

29 、

GTGTCCTAAGATTTGGGATTTACCAAGTACTGAGAATAGGACCTTCAAAAAATATGAAATGCCAAGAGGATCTGTTTTAAAGCAATGGAATTATTTGAG
AATTTTGAAAAATACAGATTCTCAGGACCCAGTCTGAATGAGAATCTCTGGGGAAGAGGCCAAGGAAACAGAATTCAGGTAAGCACCTTGGCTGCTTC
CAGTGGTTGGCCAATTTGGATATCCCTGTTGTGTCCTAAATTATACCTAATTTGATTAAAGTTTTGGTGGCTGGGAGGTTTGCTAGTTGAAAAACACATA
CAGGAAAAAAAAAATGTACAGGGGAGTGTATTGCTTTGGTGTAGTCGTTTAAGAGTTGACAAAAACCCAAACACACCCAACACAA

30 、
GTGGCGGTGACTAGCTGCCCGAAGCCACTCCCACAATTCAGTCTGCTCCTGCTGAGAGTCTCACATCTCCTGGTGCCTCCTGCTCAGGAGCATCGGTC
TCACCCTCCGAAGCAGAGCCCCTTTGCCTCATGAAGCGTTTCCAAACAGCCTACTCACAAGAATCAAGGAACGCCCGAAGCCCAGTTCCAGGGCCCT
GCCCAGGCCTGAGGAATCTAATGACAATGCCATAGTGAAGGATCCTGGCCTGGTTTAAGGTCACAAGGACACGGGGGTAGGGGAGTAGCTGAGGTTG
GGAAGAAGTGTTAGGAGTTGGTCTGCAGCACACTTCAAAGTCACGTCTTCCCATCTCGAGCCTTCCATCATCAGGTTAAATTAAACTCTGGAGCTGGG
GTGGAGGGAGGCCTGAGCCCAACCAAAGCCCAGCTGAATGGTTTGAGAGATCTGAATCAGCCGGAAGCAACTGCTCTTGTCCCCAGAAGCCGTGTCT
CAGTTCCACCTGGCATCGCCAATTATCTGTGCCTCCGACATAGGGTGTTTTTTATTCCCTCGAAATAGAGCTATAATTAAATCCAAAATTGTCCTGGAGAC
TTGATAGTCATGGCAAAAGCAGAACACAGTGTCTGCAGACAAAAAACCTCTTTCAATAAAGATTAATTATTCGCAGCCCGTGTCCCAGGCCTGGACCA
TGTGGGGAATGAATATGAAGGGGAGGGAGTACCCAGCTCAGAGGAACTGGAGGGGCTCCTTCCCTCCCCCCACAGTGCAGCCCCCCATCTTCTCTCA
GAGACTAATCGTCGACCTGCAGGCATGCAAGCTTGGCGTAATCATGGTCATAGCTGTTTCCTGTGTGAAATTGTTATCCGCTCACGATTCCACACAACAT
ACGAGCCGGAAGCATAAGTGTAAAGCCTGGGGTGCCTAATGAGTGAGCTAACTCACATTAGTTGCGTTGCG

31 、
TGTGTGTGCATGTGTCTTTATAGCAGCATGATTTATAATCCTTTGGGTATATACCTAGTAATGGGATGGCTGGGTCAAACGGTATTTCTAGTTCTAGATCCT
TGAGGAATCGCCACACTGACTTCCACAATGGTTGAACTAGTTTACAGTCCCAAAAACAAACCAAACACACCCAACACAA

32、
GGGGTTTCGCCATGTTGCCCAGGCTGGTCTCCAACTCCTGACCTCAGGTGATCCACCCACCTCAGCCTCCCAAACTGCTGGGATTACAGGTGTGAGCC
ACCATGCCCAGCCTGGTGTCCTTATTTTAACAAAGGAAATTTGGATATGCTGAGGCACCCTAAGGATGTGCACACGCAGCGTGAATGCCGTGTGAAAAT
CCAGCAAGAAGGCAGTTGTCTGCAAGCCAAGGAGAGAGTCCTTAGGATAAACCAGACCTGCCAACGCCTTGATCTGTGACGAAAAGCCTCTAGAGCT
GTGAGAAAATAAAGTTATGTTTAAACTACCCAGCTGTGGTAATCTGTTACAGCAGTACCAGCAAACTAATACAGGAAATGTCCCCATGAGCCCCCACCA
AACACACCCAACACA

33 、
GGTGTGTGTGCATGTGTCTTTATAGCAACTTGATTTATAATCCTTTGGGTATATACCTAGTAATGGGATGGCTGGGTCAAACGGTATTTCTAGTTCTAGATC
CTTGAGGAATCGCCACACTGACTTCCACAATGGTTGAACTAGTTTACAGTCCCAAAAACAAACCAAACACGCCCAACAC

34 、
TTTTGTTTTTTCACTGGTCAGAACCAGTGTTGGTAGTAAGAATTATTGTCCCAGAAGAGAATGGAAATGCTTGCCAACTCTACTTTCAACCCTTGCTGG
AATCCCTATGTTGTGGGTAGAGATATGACTAAATTAAGCAGTTTTAAGTCTAGGACACCAAACCCCAAACACACCCAACACAA

35 、
GGTAAAGGTGTGTCATGATGGTTTGCGGCACAGATCATCCTATCACCCAGTTATTAAGTCCAGTATTCATTAGTTTTTCTTTCTGATACTCTCTCTCCCCC
ACAACCAAACACACCCAACACAA

36 、
TGGTGGTTGTACTAAAAATAAGATGGGGTAGCAGGTGCTACATTTACTACCCTTTCTGAAACAACCAAACTGGCTGAAATATATAAAATACTTTTCAAGG
TACTGGAACTTAGGTGATGTCTTAGTGCATTCAGGCTGCTATAACAAAACAAACCAAACACACCCAACACAA

37 、
GTGTGGGTTGACACGAAAGGTAACAAGACCATCCGTGCGGACTGGCGCACGCCGTTTCGGCATTTTTCAACGTGGCATATGCCGGAAGCTACGCACAC
GCGCGGGATGCGAGATTGGGCGCTATTTGACGCAGCCACAAACGGCGGGACGACCTGCCCTGCGACAATCAGCTATCAAAAGGACGCGGGCGCTAGC
GGGTTTTTCCGGGTGCTAGCGTATAACCGCACCAAACACACCCAACACAA

38 、
GGGGGGGGTGACACGAACCGTAAAAGACCATCCGCGCGGACTGGCGCCCGCCACCTCGGTTTTTTCAACGGGGTTTATGCAGGAAGCTACCTACACT
CGCGGGATGCGAGATTGGGCGCTATTTGACGCAGCCACAAACAGAAGGACGACCTGCCCTGCGACAATCAGCTATCAAAAGGACGCGGGCGCTAGCG
GGTTTTTCCGGGTGCTAGCGTATAACCGCACCAAACACACCCAACACAA

39 、
GTTTTGTTTGGTAAAGGTGTGTCATGATGGTTTGCGGCACAGATCATCCTATCACCCAGTTATTAAGTCCAGTATTCATTAGTTTTTCTTTCTGATACTCTC
TCTCCCCCACAACCAAACACACCCAACACAA

40 、
TGTTTGAGCATGTCCTTAGACACATGTTCAGATATGCTTCGTTATTCCCTTTAAGCCTTTTCTTTACTTTCTCCTTCACTTTTGTTACTCTCAATGCTAATG
AATAATTGACCTCTTCGCCACAAAAAACCAAACACACCCAACACAA

41 、
TTTGGTTGCGGGTGCCTGCTACCCAGCTAGCTACCCAGTAGCGTGTAGTCCCAGCTACTCGGGAGGCTGAGGCAGGAGAATGACATGAACCCGGGAG
GCGGAGCTTGGAGTGAGCCGAGATCGTGCCACTGCACTCCAGCCTGGGCGACAGAGCGAGACTCCGTCTCAAAAAAAAAAAAAAAAAAAAGAAAA
AAGAAACTGTTATTAACAGCTCTATACCAACAAATTGGATAACCTAGAGTAAATGGATAAATTCTTAGAAACAACCAACCAAACACACCCAACACAA

42 、
TTTTTTTTCCTGTAAGGCTACACAGAAGAATTCTCAGTAACTTCCTTGTGTTGTGTGTATTCAACTCACAGAGTTGAACGATCCTTTACACAGAGCAGA

CTTGTAACACTCTTTTTGTGGAATTTGCAAGTGGAGATTTCAGCCGCTTTGAAGTCAAAGGTAGAAAAGGAAATATCTTCCTATAAAAACTAGACAGAA
TGATTCTCAGAAACCCCACACCAAACACACCCAACACAA
43、
TGTGTTTTTTATGCCTTTGCTGATCTGGGTGACTCCCTGGTTGGCTCAAAAAAACCTCAGCTCTTTTCTAAGCGAAGCTCCGCCCCCCTATAAACTTTC
ACGCTTGACCCGAAAAGAAAAAGGCCTGGGGGTATTCCTTTTCACAGCCAGTTTCACACTAGCTTTTTTAGTTCCAACCAGCGACGCCTGGTATTTGTA
CGGAATTTTTGTTCTTCTCTATTCAGCGTGGTATGGACTTTATGCTGCGATGGCCATCAATCTTTGGATTTTCTTTTTAGTCGTACCTCTTCCTTTTTTAGC
CAATCTTCCTTGGGCAGGTGATACGAACTATCTGCAACCCCAAAACCAAACACACCCAACACA
44、
GTGGGGTTTCTCTGGCCCTAAAATTATAGGTACAATTGCATTAATCCCCCACCCCCCGAACCACAGCTTGAGGAAACCACAGCTCTGCTACAATTTTT
45、
GTTTTGGTGGGGCGTGTAGAAGTCCACCAGCTGTTGAAGATGAGGCCAGGCGTCCTCAGAGGAGAGAACAGAAAGTGCCAAGGCCAGGGGCAGCGG
TAAGGAGCACACGGCACACTGGGGAAGAGTCAAATGCTGGAGCCCAGGGTGTGGGCGGAGCGGTGGGAGATGAGGACACCCCGATGGCTTGGGGTC
AGGTTCTGAAGAATGTTGGGTACTAGGCTCGAGTTTGGACTCTATGGTGGGGGAAATAGAGAGCCCCTGACAGTCTTTAAAGAGGGGAGAGATGGATC
AGGCCTGTAGCATTTCCTCAGGGAGGTTATAAAGCAGGTACAGAGGTACAGACACATCTTCTGTGCTTATTTATGTCATGGGGCCTTTGCACATAGTTGG
AGCCATGATTTACATAGCTGTGCTTCCCCATACCACCACACCAAACACACCCAACACAA
46、
GTGTGGTGGTTTTAAAACATATATTCATAAAAAAAAATAAGACTAAAGGCTTAAAAGATACCAATGCTCGAATTTTCACTAAGTGTTTGATTTGTATTATCT
AACATGGATTATTGTTTGATTTTTATTGGTATTTTTAAATGGAATGTAGGGTATTTTAATTGTAAATAATAATAGTTTACAATAAGTTGAAATTATAAGATTT
ACAAAATTAATTATTGATTTAATAAATGGGCATAATTCGAATTTTACATAAAGGTGCTTTTATTTGTTTAGAAAATCGACGAAATTAGTGCAAATACTTCA
AAATAATCAAGCTTATGGACACAGAACTCAAGCCTCTTTCAATGATTTACAATGAAAAGTCAGGTTTTCATGCCAATCAGAAAGATGATATTTATGAAA
AACTCATGACCATCTGGACCTCACATGGTTTTGAAATTCAAGTTTTTGATATTGGTTCAGAGCCTGATATAAAAACCCTGATGCAAAAAATCTTTCAACG
TCATGCACAATACTCAGATTGTGGTGTCATTGTTGCTGCAGGTGGTGATGGTACGTTGAATGCAGTCGCCTCGCAAATGTTAAAGCAAAAAATTCCTAT
GGGAATACTACCTTTGGGTACTTTTAATTATGTGGCGCGTGTTCTACATATCCCTTTGAATATTTTAGAGGCAGCCAATGTGATTGCCACAGGGGAGCCG
CGTGCCTCACATGTGGCCTGTATCAATGATCAAATTTATTTAAATAATGCCAGTTTAGGTTTGTATCCTTTGTTTATTAAAAAACGCGAAGAATATAATCGT
CGGTTTGGACGCTTTCCTTTAAATGCGCATACGTCAGGTTTAGATGTCCTGATTCGTGATCGTAAAGAACTCAAATTAGAGATTGAAGTAGATGGGCAA
AAGTATCCTGTAAAAACACCAAACACACCCAACACAA

47、
GGTTTAGGGTTATACATTTGAGAGACAGAAGAAAGCAAACTACTTCTTAACAAAATTGAACCTGTAACTTTGACCTAGTAGTCTCAGGCTTTCACTGAACTAAATGAGGCAGATTATAAACTGTGTATTGTTTAGTGGAGACTTTATGTTCTGTTAATACTCTGAATTTTAAATTGTTGTATAGATTATTATATCTAGTGTACAAATTAATACCAAATTCCGTATTAATTCAAGTATAATTGTACTTTACAAACTCTTAGCTTAAAAACAGTTTTCATTATGTGACATGTCAGAAACAACCAAACACACCCAACACAAA

48、
TTTTGTGTGTGAAAGCTCACTCTGGCTGCTGTGTCATGAACAGATTTTAGGATGCAGAGGTGGAATCAGGGAGCCCACTGAAGAAGATATTCATTGAAGGGGTCTGGGGAAGGATGATGGTGACTGGGACCAGGGTGAGGGAAGTGGAGATGATGCCGTGAAGTGCTCCAATTTGAGACAGTTCTAAGGAGCAATTTACAGGACTCAGTGAATGCGTAGGGGAGGGAGAGGAAAGCATAAAGATGTCTAAAAGCATTCCTCATCCAATTTAGATTATTAAAAAAGGATATGGTGGAGAAAGCACTGAATGTGGAGATAGACGGGGGCCCAAAACACACCAAACACACCCAACACAA

49、
GTCTAAAAGAAATGAGGAAATGACAATGATGATGGCAGCAGCTAGCAACTGTACAGCTCAATAACACCATGAAATAGGTACTGCCACTATTCCTATCATACGGATGAGGAAACTGAGGCATAGAGTGGTCACACAGCTGGAAGATGGCCAAGCAAGGATTCAAACCCAGGCAGTCGGCCTCGAGAATTCACACCTCTAACCACTCAGGACAACGCCACTGTATCATGGCGCTCCACGATCAAAGGCCCGGAGGCTGAGAGTGCTTAGAAACTGATAGAAGAAAAGTAAAGGCAGGAACAGACGGGCCTTATTACCCTTCATAGGCCTAGGAGTCTGAACCTCATCAGGCAATGTGGGGCCTGAAGAGGGTGACCAAAGGGACTTCTTGCAGGGTCCCCCCCCCCAACCAAACACACCCAACACAA

50、
TGGGGTTTGGAGCTAGAAATAAAATTTCCGATGTTACCCTGCAACCTCTGTATAGGGGTAAAAACCAACACAAACAGTCCCGCCCCTATTCCAATCAAACGGATGAGGAAACTGACATTAAATCTGGAAAGTTTAATGGAAAAGCTTCCGCTTTTCTGAAATTGAGGCAATCGGCCTCGTGAATTTTTATTTCTTACAACTCTTGATGCGCCACTGTATCATGGGGGGAAAAGATCAAGGTTTGGAGGCTGATAGTGCTTCTAAACTGATCCCCAAAAGTGAAGGCTGAACAAACCGGCCTTATTACCCTTCATAGGCCTAGGAGTCTGAACCTCATCAGGCCATGTGGGGCCTGAAGAGGATGACCAAAGGGACTTCTTGCAGGGTCCCCCCCCCCAACCAAACACACCCAACAGA

51、
TTTTTTGTTGGAGGGCAAGGAGAGTAAAATAATACTATTTTGAATGTATTTTCTCTTCTTATGTGCCATTATCTCCTTTCCCTACTCATGGGAGTAAGAGACATGTAGATAGAAGATTCACACCCACTGATTGTTGCAGCATGTCCTTTAGCTTTATGTTTTTCCCCCTCCATCAAAACTTTTACCAAAGCAGTGTTTAGTTGTCTGATTGAGTTTTATATCTTTTCTTTTTTCTAGTTAAGCCAATGAAGATGCCAGTAAGAACTGCCAAACACACCCAACACAA

52、GTTTTTTGGTTTATATATAGAGATTCAAGTAATACTATTTTGAGAGTAATTTCTCTACATGAGTGGCTTTATCTCCATTCCGTCCTCATGGGAGTAGAAGACAAGTAAAAAGAAGATTCACACCCGCTGATTGATACGCCATGTCCTTTAGCTTTAGGTTTTTCCCCCTCCGTCCAAATTTTTACCAAAGCAGTGTTTAGTTGTCTGATTGAGTTTTATATCTTTTCTTTTTTCTAATTTATCCAATGAACATGCCAATAAAAACTGCCAAACACACCCAACACA

53、GTTTTGTTGTCATAAAATTTAACTTCATTTTAATCAAGGTGACAGGTAAATTCTTAAAATACCACCCAAACACCCCAAAAATCTCAGGCTTTCGCAGTCCTAAATAATGAGCATTATTAACTGTGTATTGTCTAGTCTAGACTTTGTGTGAAATTGTTACTCTGAATTTTAAATTGCTGTATACATTATTATATCTTGAGTAGTAATTCCTGCGGTGCCTAATATTAGATCTAGTATAATTGAATTTTACAAACTCTTATGTTAAAAACACTTTTCATTAACTGACATGTCCGACACAATCAAACACACCCAAAACAC

54、GGTTTAGGGTTATACATTTGAGAGACAGAAGAAAGCAAACTACTTCTTAACAAAATTGAACCTGTAACTTTGACCTAGTAGTCTCAGGCTTTCACTGAACTAAATGAGGCAGATTATAAACTGTGTATTGTTTAGTGGAGACTTTATGTTCTGTTAATACTCTGAATTTTAAATTGTTGTATAGATTATTATATCTAGTGTACAAATTAATACCAAATTCCGTATTAATTCAAGTATAATTGTACTTTACAAACTCTTAGCTTAAAAACAGTTTTCATTATGTGACATGTCAGAAACAACCAAACACACCCAACACAA

55、GCAGTGCCAGCTTGCATGCCTGCAGGTCGACGATTTGTGTTGGGTGTGTTTGGTGGACACCGCCAAAGTCCGGCTGCAGGTAGAGGGCGAGGCGGGGATGGGGGTGCATCTCAGGGCAGAAAGGGGCACCTGTGGGGGACACCCCTTTGATTCAGGCTTTGGTTTAACCGCCGTTTCTCAGGTAGGTACAGCAGGTGGCCGCCTGAAACACCAGTTTGACGTTGTCGCGGAACTGGATGCATTAGGGTGGGAAGCTGGAACTTTTCCAGGGTTAACTGCAAAATCTCAAGGACCGCGGGGATATGAATTGACCATGATTATTGTCAGACTCCAAAAGTTAGGGGGCACCCAGTGTTCTCAGAGCAAAGACCGTCAGCCCCTCTGGAGCTGACCAGCCAAGCTCTGTCTCCTTCCATCTTTGATTCAATGGAGGGGAGGGGGGTGAATCTTCCCCTCTTGTACCGTTCTGTGAATTTATGGATCTGATGGATGGAATTTTATGAAGATCCCGTCGGAAATCCGAAGATAAGGCGCGCAGTCGGATTTTGCTCAAGATTGTTCTAGGATGGGGCCTAATTTTGTAACTTACCTCCCGTGGATGAAGGAACTGTTTCTGATACCGGTAGATAATTTTGTAGCCAGATCCATCGAGCACGTAAACACCGGCTACCAAAAAACTTGATAGATAACAACTTGTTCTGGGGGTTAACGTGAACCGATCGTATATGGATAGAATTCGAAATATGTATAGATAATTGATACTTTGAATCTTCCACCATATCTGGGGATTTTCAAGAAGGAACAACCTTCTTACATCCTGTGCGTCAATCCAGGTCTTTCCATCACGACGTGATCTGGCTGTCTTTATAGCGAATGTTTAACGATACGCCCTATGCTTAGCCTGTCCTTGCGAAGTTAAGCAGTTTTAGACGAGAGATGATATTAGCAGGTTCCGAT

56

TGTGGGTGGTACACCTGAGGTCAGGAGTTCGAGACCAGCCTGGTCAATATGGTGAAACCCTATCTCTACCAAAAATACAAAAATTAGCCGGATGTGGT
GGTGCATCTGTAGTCCCAGCTACTCGGGAGGCTGAGGCACAAGAATCGCTCGAACCCAGGAGGCAGAAGTTGCAGCTAGCCGAGATCACACCAGCCT
GGGTGACAGAGCAAGACTCCATCTCAAAAAATAAATTTAAAAACAACAAAACCAAACACACCCAACACAA

57

GTGGCGGTGACTAGCTGCCCGAAGCCACTCCCACAATTCAGTCTGCTCCTGCTGAGAGTCTCACATCTCCTGGTGCCTCCTGCTCAGGAGCATCGGTC
TCACCCTCCGAAGCAGAGCCCCTTTGCCTCATGAAGCGTTTCCAAACAGCCTACTCACAAGAATCAAGGAACGCCCGAAGCCCAGTTCCAGGGCCCT
GCCCAGGCCTGAGGAATCTAATGACAATGCCATAGTGAAGGATCCTGGCCTGGTTTAAGGTCACAAGGACACGGGGGTAGGGGAGTAGCTGAGGTTG
GGAAGAAGTGTTAGGAGTTGGTCTGCAGCACACTTCAAAGTCACGTCTTCCCATCTCGAGCCTTCCATCATCAGGTTAAATTAAACTCTGGAGCTGGG
GTGGAGGGAGGCCTGAGCCCAACCAAAGCCCAGCTGAATGGTTTGAGAGATCTGAATCAGCCGGAAGCAACTGCTCTTGTCCCCAGAAGCCGTGTCT
CAGTTCCACCTGGCATCGCCAATTATCTGTGCCTCCGACATAGGGTGTTTTTTATTCCCTCGAAATAGAGCTATAATTAAATCCAAAATTGTCCTGGAGAC
TTGATAGTCATGGCAAAAGCAGAACACAGTGTCTGCAGACAAAAAACCTCTTTCAATAAAGATTAATTATTCGCAGCCCGTGTCCCAGGCCTGGACCA
TGTGGGGAATGAATATGAAGGGGAGGGAGTACCCAGCTCAGAGGAACTGGAGGGGCTCCTTCCCTCCCCCCACAGTGCAGCCCCCCATCTTCTCTCA
GAGACTAATCGTCGACCTGCAGGCATGCAAGCTTGGCGTAATCATGGTCATAGCTGTTTCCTGTGTGAAATTGTTATCCGCTCACGATTCCACACAACAT
ACGAGCCGGAAGCATAAGTGTAAAGCCTGGGGTGCCTAATGAGTGAGCTAACTCACATTAGTTGCGTTGCG

58

GGTTTAGGGTTATACATTTGAGAGACAGAAGAAAGCAAACTACTTCTTAACAAAATTGAACCTGTAACTTTGACCTAGTAGTCTCAGGCTTTCACTGAA
CTAAATGAGGCAGATTATAAACTGTGTATTGTTTAGTGGAGACTTTATGTTCTGTTAATACTCTGAATTTTAAATTGTTGTATAGATTATTATATCTAGTGTA
CAAATTAATACCAAATTCCGTATTAATTCAAGTATAATTGTACTTTACAAACTCTTAGCTTAAAAACAGTTTTCATTATGTGACATGTCAGAAACAACCAA
ACACACCCAACACAAA

59

CAAATATTCTTCAGACATTTCTTTATGCATACACATTTATAAAGAAAATAATTTTATCTTTACAAAAATGAGGACACATTATACACACTATTCTGCAAGTTG
ACTTTTTCTCTACATGAGAGTAAGGCAATATAGTATGGTGAGTGGTTAAGGTTACATGGACTTTGAATTCAGACAGATAAGGGTGTGAGTCCCATCACA
GGGATAGTATCTCTTACCCCCTAGTTTTGTTGTGAGAAGTGAGATGATGCACACAGAACATTTTAAACTGTGTTTTGGCACATGATGAGTTTTTAAAAAT
ATTAACTAGTGGCGGGGCGTGGTGTTGCACCCATAATCCCAGAACTTTGGGAGGCTGAGGTGGGTGGATCACCTGAGATCAGGAGTTCGAGACCAGA
CTGACCAACATGGTGAAAACCCTGTCTCTACTAAAAACATACAAAACAAACCAAACACACCCAACACAAA

60

TTGCGTATTGGGCGCTCTTCCGCTTCCTCGCTCACTGACTCGCTGCGCTCGGTCGTTCGGCTGCGGCGAGCGGTATCAGCTCACTCAAAGGCGGTAATA
CGGTTATCCACAGAATCAGGGGATAACGCAGGAAAGAACATGTGAGCAAAAGGCCAGCAAAAGGCCAGGAACCGTAAAAAGGCCGCGTTGCTGGCG
TTTTTCCATAGGCTCCGCCCCCCTGACGAGCATCACAAAAATCGACGCTCAAGTCAGAGGTGGCGAAACCCGACAGGACTATAAAGATACCAGGCGTT
TCCCCCTGGAAGCTCCCTCGTGCGCTCTCCTGTTCCGACCCTGCCGCTTACCGGATACCTGTCCGCCTTTCTCCCTTCGGGAAGCGTGGCGCTTTCTCAT
AGCTCACGCTGTAGGTATCTCAGTTCGGTGTAGGTCGTTCGCTCCAAGCTGGGCTGTGTGCACGAACCCCCCGTTCAGCCCGACCGCTGCGCCTTATCC
GGTAACTATCGTCTTGAGTCCAACCCGGTAAGACACGACTTATCGCCACTGGCAGCAGCCACTGGTAACAGGATTAGCAGAGCGAGGTATGTAGGCGG
TGCTACAGAGTTCTTGAAGTGGTGGCCTAACTACGGCTACACTAGAAGAACAGTATTTGGTATCTGCGCTCTGCTGAAGCCAGTTACCTTCGGAAAAA
GAGTTGGTAGCTCTTGATCCGGCAAACAAACCACCGCTGGTAGCGGTGGTTTTTTTGTTTGCAAGCAGCAGATTACGCGCAGAAAAAAAGGATCTCAA
GAAGATCCTTTGATCTTTTCTACGGGGTCTGACGCTCAGTGGAACGAAAACTCACGTTAAGGGATTTTGGTCATGAGATTATCAAAAAGGATCTTCACC
TAGATCCTTTTAAATTAAAAATGAAGTTTTAAATCAATCTAAAGTATATATGAGTAAACTTGGTCTGACAGTTACCAATGCTTAATCAGTGAGGCACCTAT
CTCAGCGATCTGTC

61

TTGTGTTGGGTGTGTTTGGTGTGTTTTATGTATGTATGTATGTATGTATGTATGTATGTATGTATGTATGTATGTATGTATCTATGTACTCCAATTTAGACACT
CCACCTTCTCTCCAATCCTAACATATTTTATGGTTTGACCGGAAGTGATAGAGTAATCAACTGTTCTGCCTCCCTTAAACTGACCTGACCTCAACCCCCT
TGATGTCTAATCCAAAGCTCTTCACCTGTATCACCCATAGCACTCCCGTGACTTCCTCCCATCCACCCAGCACATTCCAAAGCCATCTGTCTCCTACAGA
TAACCATGAACTCTGATGTGACAACCATTCTCTCTCACCCCACCCCCAAACACACCCAACACAAATC

62

TTGTGTTGGGTGTGTTTGGGTTGTTTTTGAGGTACCTGGTCAGAGCTAGGAGCAGTTCCCACTGCTAAGGGAAATTACAAGGCTTTGTTTTTATTACAC
ACCCTGCTGCTTTCCACAAAGGTTTTAAGACTGGGAACTGAGCCTCCTCTATCTGAACAAGTAGAATAGAGGCCTGAAAGCACAAAATTGGCTTTGTG
TCCAGCAGACACACCCATTCTCAGGAAGGTGAGAATTCCCTCCCTGCTGAGAATGGAAGTAGGACAGAGAGAGAGGGAGGCCAGCATGAGGGTGAG
TCTGTCTTACCTCTGGAGTATCCTGGAATGGAATGAGTGAGTGTCAGAGGAGAGAACTGTCACTTCCCCAAAACCAGCCTGGCAGGAGAAACCATGA
AGTCTTAACACAGTTCCACTGCCACCTGGTGGCGGCGTACTCAATTTGCAGGCTTAGTGTGTACCCAGTAGTTCTCAGTCACATACTATACTCAGTCAC
ATACCTAGAAAGACCCATGGTCACTCCTAGTCCCCAACCCACCAAACACACCCAACACAAATC

63

TTGTGTTGGGTGTGTTTGGTGTGTGGTGTGGACTCACAGAATGTTGAGTGGAATTTCATGTACCCTTGTAGGGGTACGTGTCTCAGGTTGGTGGAGAA
GACAGTAAGTCTTCCTAAAAGTGTATGGAACTTCTTAAAAATGCAGAGAGAGAGAGTCTGTATACACAAGAAATTGGAACTTCTATGGGTTGCAGCAT

GGAAGTTTAATTATTCTTTCTCTGGCTTTAATTTTAAAATGTATTTAAGAGTACAGGAACCAATTAAAGATGGCAGAGTAAAGACATTTCTGCCCCGTTC
TTTGAAACCAACTTAAAACCAATACAAAGAACAACCCCACCAAACACACCCAACACAAATC

64

TGTGTTGGGTGTGTTTGGGTTGGTTTGGCTATTGATTATTGCCACAATTTCAGATCCTGTTATTAGTCTATTCAGAGATTCAACTTCTTCCTGGTTTAGTCT
TGGAGAGTGTATGTGTCGAGGAATTTTTTGCCATCCCCATCAAGCTACCAATGACTTTCTTCACAGAATTGGAAAAAACTACTTTAAAGTTCATATGGA
ACCAAAAAAGAGCCCACATTGCCAAGTCAATCCTAAGCCAAAAGAACAAAGCTGGAGGCATCACACTACCCGTCTTCAAACTATACTACAAGGCTAC
AGTAACCCAAACAGCATGGTACTGGTACCAAAACAGAGATATAGATCAATGGAACAGAATGGATCCCTCAGAAATAACGCTGCATATCTACAACTATCT
GATCTTTGACAAACCTGAGAAAAACAAGAAATAGGGAAAGGATTCCCTATTTAATAAATGGTGCTGGGAAAACTGGCTAGCCATATGTAGAAGGCTGA
AACTGGATCCCTTCCTTACACCTTATACAAAAATTAATTCAAGATGGATTAAAGACTTAAATGTTAGACCCACAAAACCAAACACACCCAACACAAATC

65

TTGTGTTGGGTGTGTTTGGGGGGGGGGGTAAAAAGCACAAAAAAAATGTAAAATCTCGGGCAATCATGCCGATTCTCTCCTTTATTAATTACGATGTTCCT
ATTAACGCTGTAGTAAATCATGACCCCATTTGAATTAATACCTCCCCTTAAAAATGGTTTAAAACAATGTACCTTTACATTATAACTGATACGTAAATCAAT
TTATGTTGGAGCTTCTCATAGACTTTGAGATATTTCGCACACTGCCAATCTTTAAGGGAAGTCTTTATGACCGACTTAGGCAGAGCTTTTGGTTGAGCAT
CTTTTGAAGCCTAGGATTGTTTGAACGCGGGTTGAATGTCTTTTTTGTATCCTTGGCTGTTGTGGCGGGGGGAATATCTTTCACTTCTTTTTCGG

66

TTGTGTTGGGTGTGTTTGGTTTGGACTGGAATGGAATGGATTGGAATGGAAATGAATGAAAGGGAATGTAATTGACCTGCGTGTGATGCAATTTACTGT
AAAGAAAGGTATTGCTGTTGTATGGGATGCCTATAATGGATGTTGCTCCACACACCACCAAACACACCCAACACAAATCTCTTTAGGATCCCCGGGTAC
CGAGCTCGAATTCGTAATCATGGTCATAGCTGTTTCCTGTGTGAAATTGTTATCCGCTCACAATTCCACACAACATACGAGCCGGAAGCATAAAGTGTAA
AGCCTGGGGTGCCTAATGAGTGAGCTAACTCACATTAATTGCGTTGCGCTCACTGCCCGCTTTCCAGTCGGGAAACCTGTCGTGCCAGCTGCATTAATG
AATCGGCCAACGCGCGGGGAGAGGCGGTTTGCGTATTGGGCGCTCTTCCGCTTCCTCGCTCACTGACTCGCTGCGCTCGGTCGTTCGGCTGCGGCGAG
CGGTATCAGCTCACTCAAAGGCGGTAATACGGTTATCCACAGAATCAGGGGATAACGCAGGAAAGAACATGTGAGCAAAAGGCCAGCAAAAGGCCAG
GAACCGTAAAAAGGCCGCGTTGCTGGCGTTTTTCCATAGGCTCCGCCCCCCTGACGAGCATCACAAAAATCGACGCTCAAGTCAGAGGTGGCGAAAC
CCGACAGGACTATAAAGATACCAGGCGTTTCCCCCTGGAAGCTCCCTCGTGCGCTCTCCTGTTCCGACCCTGCCGCTTACCGGATACCTGTCCGCCTTT
CTCCATTCGGGAAGCGTGGCGCTTTCTCATAGCTCACGCTGTAGGTATCTCAGTTCGGTGTAGGTCGTTCGCTCCAAGCTGGGCTGTGTGCACGAACCC
CCGGTTCAGCCCGACCGCTGCGCCTTATCCGGTAACTATCGTCTTGAGTCCAACCGGTAAGACACGACTTATCGCCACTGGCAGCAGCCACTGGTAAC
AGGATTAGCAGAGCG

67
ATCAGCTCACTCAAAGGCGGTAATACGGTTATCCACAGAATCAGGGGATAACGCAGGAAAGAACATGTGAGCAAAAGGCCAGCAAAAGGCCAGGAA
CCGTAAAAAGGCCGCGTTGCTGGCGTTTTTCCATAGGCTCCGCCCCCCT
68
TTGTGTTGGGTGTGTTTGGTTTGTTGGTGTTTTAAAAAAATAAATATGTGTGTGTGTAGATAGATAGCTAGATAGATAATCTCCATTAGCTACCACCTGGA
TCAAATAATAGAATATGTTCACTACCCCAAACACACCCAACACAAATC
69
TTGTGTTGGGTGTGTTTGGTGTGTTGGTTCTATGTTGACTGATATGGTGTTTTGCATGTAAGAATAACCTAGGAAAATTCAGTACATTTAATTGTTTAATT
GAAGGGCAAAAATGAAAAAAAATTGCAAAGGATACTGAAATCACCTTCTAGATTTATCAGATGGTTTTAACAGCAACTAATAAAAAACCCCTAGAAAT
AATTAAACTCTTATGTCAAGAAAGAAGAAATATACATTTGAAAAAAAAACCCAAATTCCCCCAACACAAATCTCTATGGGATCCCCGGGTACCGAGCTC
GAATTCAACATCATGGTCATAGCTGTATTCTGTGTGAAATTGTTATCCCAATGCAATTCCTCACAACATTTTAAACTGAAGAATCAAGAGGTGAATTTGC
GACGTCTATAGAGCGAAATAAATCACATAAATTGCCATGTAATCACTATCCACTTTCCGATTATGAACCCTGATTAGCCAATGGATTTAATGCATCGGGCA
ATCCGCTGGGAGAGGATGACCGCAATGTGAAAATGCGTTAGCTTCCTCGAGAACCCCAAACCCAAACACACCCAACACAAATC
70
TTGTGTTGGGTGTGTTTGGGGTTTGGGTGCATAGTATTCCATGGTGTATATGTGCCACATTTTCTTAATCCAGTCTATCATTGTTGGACATTTGGCTTGGTT
CCTAGTCTTTGCTATTGGGAATAATGCCACAATAAACATACGTGTGCATGTGTCTTTATAGCAGCATGATTTATAATCCTTTGGGTATATACCCAATAACGG
GATGGCTGGGTCAAATGGTATTTCTAGTTCTAGATCCCTGAGGAATCAAAACACTGATTTCCACAATGGCTGAACTAGTTTACACCCCCCCAAACACA
71
TTGTGTTGGGTGTGTTTGGTTGTTGGTTTGCAAGATCTGGTAATAACCAGGAGACAGGATGTTCTAACAGGAAGTACTGTGGCTTGGGAGAGACAGAT
TCTACTTGTAAAATGAGGGCATTGAGCTGAATGATTTTTCATGCCTTCTGACTGTTAACATCTGAGGTTCTTGCATATAGAGCTTCCTTCAAAGCCTGAG
ACATTCAGACAACTTGCCCCTCTTCCAATTAGGGAACTGATCTTTGGAGTCTTCCACACCCCAAACACACCCAACACAAATC
72
TTGTGTTGGGTGTGTTTGGTTGTGGGTTGCGCTGGATCAAGGAATTCGCCACTCCACCCCCATGTTCCCTCTCAAAGAGGAAGAAGAAAGAGAGCCTA
GTAAAAAGCCCTTCCCCAGTGAAAAGCCGTGGGGTCCCTCCATACATGTCACCAAATAGGGGACAGGAAGACCAAGAATCAAGAGGCAGCAGGAGG
GCTAGAGGAAGAAAGACCTGGAGACCATGGGGAGCCAAACCAACTGCGCCTTTAAATCCTTATCCAAAACAAAACCAAACACACCCAACACAAAT
73

TTGTGTTGGGTGTGTTTGGTGTGTGTGTGTGTGTGTGTGTGTGTGTGTGTCTTCCCTCTCACAGGCAGAGCAGACACACCAAGCTGCTGACCATCAAG
GGTCGGCAAATTACAGACCTACCTCTCTTATGAGCCCCCATACCAAACTGTGAGTGGCGGGAAGAAAGCAGGGTAAAAACCACCGAAACACACCCAA
CACAAATCTCTAGACGATCCCCGGGTGCCGAGCTCAAACTCCTGCGCCTTTAAATCCCTGTTC<u>CCT</u>GTGTGAAATTGTTATCCAATACAAAT

74
TTGTGTTGGGTGTGTTTGGGGTTGGTGTGCTTACTTTAATTCTGTTACAGATTTAAAGTAGGCACATCTAACCCTAAAACCTGTTTATTATTTTTGTAACA
CAAATAAAAGCCCGAACTTGTCGGGCTTTATTAAAACAAACACCAAACACACCCAACACAAATC

75
TTGTGTTGGGTGTGTTTGGTTTTTATTTTTTTCCATAAGTTATTGGCGTACAGGTGGCATTTGGTTACATGAGTAAATTCTTTAGTGGTGATTTGTGAGATT
TTGGTGCACCCATCACCCAAGCAGTATACACTGCACCATATTTGTAGTCTTTTATTCCTCACCGCCCTCCTACACTTCCCCCCCACCCCAAACACACCCA
ACACAAAT

76
TTGTGTTGGGTGTGTTTGGTTGTGTGGTGTTGCTCTCATGGAGAACCTCTGCTAGGGCAGTGCAGAAGGGAAATGTGGGGTCAGAGCCCCGGTACAGA
GTCCCTACTGGGGCACCACCTAGTGGAGCTGTGAGAAGATGGCCACCAACCTCCAGACCCCAGAATGGTAGATCCATCTACAGCTTGCACTGTGAACC
CACCCACCAAACACACCCAACACAAATC

77
TTGTGTTGGGTGTGTTTGGTTGGGTGTTCTGGACTATTTGAGTGGGCCCTAAACGCAGTCTCAAGTATATTTATAAGAGGAAGACAAAGCCGGGCGCGT
TGGCTCACGCCTGTAATCCCAGCACTTTGGGAGGCTGAGGCAGGCGGATCACGAGGTGAGGAGATTGAGACCATCCTGGTTAACACGGTGAACCCCT
GTCTCTACTAAAAACAACAACCCAAACACACCCAACACAAATC

78
GCAGCTTGCATGCCTGCAGGTCGACGATTTGTGTTGGGTGTGTTTGGTGGGGGTGTTCTACGGGGTTGGGGGCTTGAATGATGCTCTTGGGTTTTTTCC
TCTGCCTTTCTCTTCTGCTGGGCCTGTGGGTGTTAGGGGGCACCGTCATCTCCTTTCTGTGGCTCTGTCTTCCTTTTTGCTACAGGGAGGAAAGGGGAT
TGCCCTAGGACTGTTAAGAAACAATCCTATTTTCAAAGGTCTGATGAGTCATGTCCATGTATAAAGAAATGCTGCACATTAGTCTGCAGCAAGAGCTGT
GTAGTCCCAATCTCTAAGCAGAAGCTGGGAACTGGAACCATTCTCTCAGAGTATGTGCGGTGGTAAGGGACAGTGGAAAAGCCCAGGCTTGGGTTGC
CAGAGACCTGGGACAAAGACTGAGGTTCACCAGTGACTGGCCAGCAGGTCCCTGGGCACATCTCTCTGGTCTCTCTGTGCCTCAGTTCCTCCCTGTGT
AAGATGGAGAAATGCCTACTTCAGAGGGGGAAAATTTTATATATTACATATTATATATATATAATTATATATATACACACATATGTGTATATATATATGTGCAC

ATACATATATACAAATTGGATAAACATGTGCTTGTCACAAGAAATGTGGAAGAAGTGATAATGGCTACTTCTGGAGAAACATGTTTGGAAAACCACATA
CATTTAAAGGCCAGCATCACACAAGTGGGTTAACAGCATAGGTGCATTTTGTTACATGACTGTGAAATTGTCTTTGAAAGCAGATGGAGAATACCTTTG
GGATTTTATGTTAGGCATTTAATGAGGGCCAGTGAAATGAAAGAATGGTTATTTTATCAGGAGGAAAAATGAATCCTTTTTTATAGGTGATGTTGGCTTG
TGACATTGTCTCTTACTCAGGAGTGTGGGACCTATAAAAAAACAACCAAACACACCCAACACAAATCTCTAGAAGATCCCGGGTACCGAGCTCGAATT
CGTAATCATGGTCATAGCTGGTATCCC
79
GCAGCTTGCATGCCTGCAGGTCGACGATTTGTGTTGGGTGTGTTTGGGTTGGTTGTTAAATAAATATTTTAGTTGGGTGTTTGTGTAGCAGGTGATCTTG
CTGGCTCCCTGTACTGTGTCCTGTGTGGGGGGCACCTGGTGCCCATTCACTGAATGTGTCTGATGGATTTACCACACTCATCTGGGAACCTGTGGGATT
GCCCTCCCCCTAAATCTAAACAATGCTATTTCTCAACGGCTCTGGAGGGGAGTTTGTGTATAAAGAATTGATTTTCATTAGTCTGCTACCCTAGCTACTCA
TCCCCAATATCTAATATCATTCTGTAAACTGAAACCATTCTCTCAGACACTGTGCCCAGCCAAGGCCCAACGGAAAAGCCAAGGCTTGGGTTGCCATCC
CCCTGTACACAAACACTGATGTTCACCCATGACTGGCCATGATGCCCCTGGGAACATCTCTCTGGTCTCTCTGTCCCTCAGTTCCTCCCTGCGTAAGATG
GAAAAATGCCTACTTCAGAGGCGAAAAATTTTATATATTACATATTATATATATATAATTATATATATACACTCCTATGTGTATATATGTATGGCCACCTGCATA
AATGAATCTTGCATAAACCTGTGATAGGCACTTTACATGTTGAGCACTTTATAATGTCTACTTTTGGAGAATCATGTCTGTCAGACCTCCTACTTTTAAAA
GCCATATTCACTCACTTGGATGAACTACTACGGTGCTCCTCGGAACCTGGGTATAACACTGGATATAACATGAGATGAAGAAGACCTCTAGGAGCCTAT
GACAGTAATTAAGTCAGGTTCCTGGAGATTTTCCATATGCTCTTTCCCCTGACGAGAAACACAAAAATCTACGCACAAGTGATGTGTGGTTAAGCCCTA
GACGCCTACACAGATACCGGTGGGACTCCCCCTGAAAGAACCCTCATGCGCTCAACTGTCCGACCCTGCGGCCCCCGGATACCTGTCCGAATTCTCAC
TTCGGGAATAGCGGCGCT
80
GTGCAGCTTGCATGCCTGCAGGTCGACGATTTGTGTTGGGTGTGTTTGGTTGTTGGGGTTGAAATCTCTGAGCTGAGATCCCACTTGGTCACTGCTTGT
GTGTCCCTAACCAAATGGCCTCAGCATTTTAGACCTCATTTCTCCCGTTGTCAAATGATGGAGCTTGACTGCCTGATTTTCCTCGAGTAGAAACCCCCA
AACACACCCAACACAAATC
81
CTGCAGCTTGCATGCCTGCAGGTCGACGATTTGTGTTGGGTGTGTTTGGGGGGGTCTGTGTTCTTTGCGTTTAGCCTGAATAGAGCTTTATGACTGTCTT
TACCAATAAAGCATGGCTCAATGGAGGCTCTATGACTTCTGAGTCTAGGTCATTAAAAAAAAACAAAAAACCAAACACACCCAACACAAATC
82
ATGCAGCTTGCATGCCTGCAGGTCGACGATTTGTGTTGGGTGTGTTTGGGTTGGTTGTTAAATAAATATTTTAGTTGGGTGTTTGTGTAGCAGGTGATCT

TGGTGGCTCCCTGCAGTGTGTCCTGCATGGTGAGCACCTGGTGAGCATTCACTGAATGTGTCTGATGGATTTACCACACTCATCTGTGAACCTGTAGCT
CAGCTAATCCCCCTAAATCTGTGAGTGGCCCTTCTCAGCTGCCCTGCAGGGGACTTTGTGGAGCAGGAATTTATTTTGACCAGCAAACTACCCTTGTGA
CTCATCACCAAGAGCTTCTATCATTCTCTAGCATGATTTGGAAATTAAAGACACTTTGACCAACCAAAACCAAACACACCCA

83

CGTGCCAGCTTGCATGCCTGCAGGTCGACGATTTGTGTTGGGTGTGTTTGGGGGGGTGTTGCCAAACGAGTACGGCGCAATGCTCGCGTTCATCAGCC
ACAGCTGTCGCCCGCGCACATCGACCCAGGCATCTTGCAGCGACCCACCGTGCGCCCGCAGCGACTTAATCTCCGTCCCGAGCAACACAATCCCTGCC
TCAAACGTCTCGAGAATCTCATAGTCATGCCCTGCCTTGCGGTTGGAGACGAGCTCCGGATCGGGGGCTTTTTCTTTGGCCATGGGTTTATCATAGCGA
AAAGAGGGGAGTGAAGGGAAGGTAATCATTTTTTGAGGTTGTGAAAGAAATTTCAAATGGGGTACAATTTTGGGCACAAAACGAGAGGAACACCATG
ATTCAAACCACCAAACACACCCAACACAAATC

84

TCCAGCTTGCATGCCTGCAGGTCGACGATTTGTGTTGGGTGTGTTTGGTGTGGTTTTGAAAGTGGTTGGGGTGTTCAGCCCTGCAACGACATCGAAGA
CGCTGGCAAGTTGCCGATTGATTTTGATTACTACGTCAGAGAAGTGGAGAAACTATGTCTGAGTCTAGCTTGAGCAAACAAGTTGACGGGAATCACTA
CAAAGACCAAGCAATTCAACCCGTTGAGTACATTCATGCCAATCACATCGGTTATTTTGAGGGGAACGTAATCAAATACGTGACCCGCTGGCGCAAGA
AGAACAGGCTTGCTGATCTTGAGAAGGCCAAACACACCCAACACAAATC

85

GCAGCTTGCATGCCTGCAGGTCGACGATTTGTGTTGGGTGTGTTTGGTGTGGTTTTGAAAGTGGTTGGGGTGTTCAGCCCTGCAACGACATCGAAGAC
GCTGGCAAGTTGCCGATTGATTTTGATTACTACGTCAGAGAAGTGGAGAAACTATGTCTGAGTCTAGCTTGAGCAAACAAGTTGACGGGAATCACTAC
AAAGACCAAGCAATTCAACCCGTTGAGTACATTCATGCCAATCACATCGGTTATTTTGAGGGGAACGTAATCAAATACGTGACCCGCTGGCGCAAGAA
GAACAGGCTTGCTGATCTTGAGAAGGCCAAACACACCCAACACAAATC

86

TCCAGCTTGCATGCCTGCAGGTCGACGATTTGTGTTGGGTGTGTTTGGTTTTGTTTTAAAAGTCACAAGACAATGCTTCAGGAGAAAATCTGGGGAGG
CGGTGTCGGGGCAAGAGGTGGAGTGGGGGAAATGCCTATGGAAGGGGAAAAACTCATCAGTGGGACCGACCCACTCCATGCGTTGACGGAACTCAC
ATCTATAACCGAGCCCGGCGAACAGATGAGTACATTCTTGTCAATCTCATAGTCGATTTGGGGGGGAACTTATTCAAATAC

87

GTGCAGCTTGCATGCCTGCAGGTCGACGATTTGTGTTGGGTGTGTTTGGGTTGGGTGAAAGTAAGGATAAATAGAAAAAGAATCACAATTAAGAATAG
TTTTAAACATTAGGAAGACAAAGATTTTGAGAGACAAGCAAAACATTATCAGGATGCACTGCCCCTTTCCATTCCAAAGAAAAGATTAGATAGGGAAG

GTTCCAAATTGCAGTGTTTGCTAAGAATTGCATTAGCTGATCAGTGTAGGAAGAAATCAGCAGTAAATATACTGATCAAAAGATATTCTGGAGATGAAAT
CTTTTGCTTTTTAATATCCGAAGGTCTTTTCTGAATGGAAAAGGCTAGTCCAGGTTAAGAGAAAGGGAGAGTGCTTCTTTTGAGAAATTATTCAGTAAA
GCCTACCTTTTGCCAACAGCCAGGCATGGGTAAGCCATCTGGCCCCTGTTTTAAAGAGAAAAAAAACCAAACACACCCAACACAAATC
88
GCAGTGCAGCTTGCATGCCTGCAGGTCGACGATTTGTGTTGGGTGTGTTTGGGGGGGGGTTGATTAACTGGAGATATTTTGTTTGTAATTTCCCTTTTTA
TTTTTTTTTATTTTTTATTTTTTTTACAGATAGCACTTTTATTTGCCACTATTTGAAGTCTGAACTTTAAACAGATTCTTGGACTGGTGGTTCATATCCATCA
GCTCATTCAACTTTAGCACCCGTCTCGTCCCCAGCAGCTTTTCCAGAACTACTGCCTTCACCATGAAGCTCCATGAACTTTCCCAGTTCAAACTTGGGC
TTCTTCAACATTTTTACTTTTCTAACGAAGACATCATAGAGAGGATAAATAGATTGGCAAGTCTTTTCTATGTCTTTTCCAATGCTGTCTGGAATCAATTT
ATTGACCACACCACACACCAAACACACCCAACACAAATC
89
AGCAGTGCAGCTTGCATGCCTGCAGGTCGACGATTATCCTTTCTTCCACTTAATCGAATCGGCTATTGAAGCTTTTGTATGTGTCCTGAAGTTCTTGTGC
CATGGTTTTCAGCTCCATCAGGCCATTTAAAGTCTTCTCTACGCTGTTTATTCTAGTTAGCCATTCGTCTAACCTTTTTTCAAGGTTTTTAGCTTCTTTGTG
ATGGGTTAGAACATGCTCCTTTAGCTCGTAGAAGTTTGTTATTACCGACCTTCTGAAGCCTGCAACCCCAACCAAACACACCCAACACAAATC
90
GCAGTGCGAGCTTGCATGCCTGCAGGTCGACGATTTGTGTTGGGTGTGTTTGGGTTGATGGGTGTGACTGACTAATAAGTCCTAAATGTGAAAGTGATC
CGAATGCTTGGGTGATGGGCACCCACTGGCTGATCAACAATGGCGCAACCCTACAGCGGATTACCATGCGCTTGGCCGATTACTATCGATTGCCTCACC
AGCCGATTGAATTGCATCAACCCGTTGCTGCCACGATGCATTTGAAGTCCCAATTGAACCTATCCGACGATGCGAAGGTTGAAGCTTGGGCCAAATTGC
CGGAGACCCTGGCGCTACCCAGCATGAATCTCAGCAAACGATACCATGCGGGGCTGTTGGAAGTTAAAAACCCACACTATCTGAAAAAAAACCGTCC
CGCTGTAGCCGCTGAAGATAAACAACCCGACCCCGATGAAAGCAGCTATGACCGATAAGAGCAAAACCGTTCTGAGTGACTTGCCCGAACACTTGCG
CCGTATTCGTAGTTTTGTGCGTCGTGAAGGACGTCTGACCGAGGGACAATCTTCGGCGATTGAGACCGGTTGGCCACGATGGGGCGTGGACTTTACGC
CTGGCATGACCAATTGGGATACCATCTTCGGTCGTTCCGCACCACGCTACCTCGAGATTGGCTTTGGCATGGGCGGGGCAACGGCACAAATCGCTCGC
AATCATCCGGTCAACGATTACCTGTGTATCGAGGTGCATCGCCCCGGTGTTGGCAACCTGCTCAAGCTCATTGCGCAGGATGGCCTGACGAATCTGCGC
ATCATGACGCACGACGCTGTGGATGTTGTCAGCGAGATGATTGCTCCAGATTCGCTAGACGGCATCCTGATCTTTTCTCCGGATCCCTGTCTCAAGAAG
CGGAAATACAAACCCAAACACACCCAACACAAATC
91
CGCAGTGCAGCTTGCATGCCTGCAGGTCGACGATTTGTGTTGGGTGTGTTTGGGGGGACGCCAACAAAACAAAATCAAGTGCATTAATGGATCCTGAA

TCAAATTGTGATCCATTCCCAACTCCACACAACTTATAGACGGAAGCATACCAATTGAAGACTGGGGTGCCGAGTGTGTGATCTAAGTCACATTAATTG
GGAAGGGCTAACTGCCCGCTTTCCAGTCTGGAACCCTGTCGAGCCAGCTGCATTTAGGCCCGGGAAACTAGCGGGGAGAGGCGGTTTGCGTAAGGGC
GATTTTCAAAAGATACGATAACTAAATCGAATCGATCACTCAATCGGCTGCGGAGAACGGTATCCTCTCACTCCACCGCTATCATATATTCATCCACAAA
AAATGGGCAATGGGTGAGGAAGCCATGTGCAATGGTGACATCAAAAGGTCGGTATGTAAAAAGGATCGTAGAGGGCGGTTAACATAGATGCATCCCCA
CTGTCCGTATCCCAAAAGTCCACGAACAGACCAAGGTGACAACCCTAATTGGACTAAAATTTACAAAAAATTCCCCCAGTTAAGCCACTACTGCGTTG
TCGGTTCCTTACCACCGACCAAAAAACACAT

92

GAGTGCAGCTTGCATGCCTGCAGGTCGACGATTTGTGTTGGGTGTGTTTGGTGGGTGTGTGTTCACATAAGCACCTAAATTTTTGGCAGAGAGAAGGC
ACCAGATTGGCAATAAATCACCTGAGAATAGCTTTTCAGTATGAAAAATGTTGGACTCTATGATGCTTCTTATGTAACATTATCAAAGATACCCATTTCAC
TACCATTTCATCTTAGTCCAAACACACCCAACACAAATC

93

GAGTGCAGCTTGCATGCCTGCAGGTCGACGATTTGTGTTGGGTGTGTTTGGGTGGGGGGAGAGCATCAGGAAGAATAGCTAATGGATGCTAGGCTTAA
TACCTAGGTGATGGGATGATCTGTACAACAAACCACCAGGCACACATTTACCTATGTAACAAACCTGCACATCATGCACATGTACCCTGAAATGTAAAAT
AAAAGTTGAAGAGAAAAAAAGTTCAGGAAATTTATGAAAAACAAAACCTCTTCAGGCCAGGCATAGGTAGCTCACGCCTGTAATCTTAGCACTTTGG
GAGGCCAAGGTGAGCAGATCACTTGAGGTCAGGAGTTGGAGACCAGCCTGGCCAACATAGCAAAACACTGTTCAACCAAACACACCAAACACACCC
AACACAAATC

94

GCAGTGCAGCTTGCATGCCTGCAGGTCGACGATTTGTGTTGGGTGTGTTTGGGTTTGTTGGTTGGAATAGTTTCAGAAGGAATGGTACCACCTCCTCCT
TGTACCTGTGGTAGAATTCGGCTGTGAATCCTTCTGGTCCTGGACTTTTTTTGGTTGGTAAGCTATTAATTATTGCCTCAATTTCAGAGCCTGTAATAGAG
AAGTTGTCTCTATTCAGAGATTCAAGTTCTTCCTGGTTTAGTCTTGGGAGAGTGTATGTGTCTAGGAATTTATCCATTTCTTCTAGATTTTCTAGTTTATTT
GTGTAGAGGTGTTTATTGTATTCTCTGATGGTAGTTTGTAAAACAACAACCAAACACACCCAACACAAAT

95

GCAGTGCAGCTTGCATGCCTGCAGGTCGACGATTTGTGTTGGGTGTGTTTGGGTTCAGGTGAAGCTGGAAGTTCAATGTTCTCAGATGTATCCACCCA
AATATCCCCAGCTCATATCTCACAGTCCCACTCCTTCTCTAACAGGGATCTGACTTGAGCCTAAAAGACCTGTTAAAGCTGCATGTTAAACCCTCTTTGC
AGCTTTGCCTTCCTTATCATTCACTCCTGGCCTTGGTTTTTAGCATTGCTGGTCCTGTACCACACCCAGTCCACCAAGTGAAAATCTCAAGAATTTAGAT
GCTCAGTAAATTAAAAGTTTCCACACACCACACTGGATGCCTGCAAAGGAGTCCTTATTGCCATCTGTCTGGTTAATGATCCAGTTCCAGAATCCCATAC

TGAAGAGGAGCTTTTTAGGGTGACTTCTAGTATCAGCTGTCATAGTTGATCTCTCCCTAAAAGCAGAACCTGAAACAAGGTCAGAGATGCAGGTAGTT
GACTTGGGAGATGATGCTAGGAAACTGGAGTAAGGGAGTAAGGACAGCAAGACAAGGAAGGAGAAAGACCAATAAAATGTATATGGTTGGTCTGGTT
ACACTGTGGGCTACTGGAGCTCAGTCCTGCTGCAGATCCTCTGAAGAACTACGTAGAACACACCTTAAAATTGTTTCTCCAGGAACAGAGGACAGAA
ACACTTGCTCACTAACTACTATCCCCCATAGTTCCCCTGAGGATGTAAACTGTCCTGCTCAGCAACTTTCCACCACTTTGGAAAAACCCCACCAAACAC
ACCCAACACAAATC

96

TTGTGTTGGGTGTGTTTGGTGTTGGTTGGGTGCTGTGACTAGATGCTTCATGTGAGAGGGTGAACACAGTAAAAGGGAAATTTAGTTCGTTCCATAGGA
GTTGCCAGTCTGGATCAGATTACTGATCCATAAATTTTGGATACCTTCCCATAGTAACAGGGGCTTATTTCAAGAGCTTATCAAGAAGTCAGAATTATTTC
ATCAGATTCCAAGTCAAATGAACTATGCAAAAGAAAGTTGAAAAAGATTTGAGAAGAGATTAGATGAGAAATTAAAAATCTGAAACAATGGCCTATTA
GTTTTTTGAATAATAAGTTGTACTACTCCTTTTGAATATGTACATTACATATAATATTCACTAATAATTAGGTCATTTCATTAAATTGTACTCTGGCTGCAAG
GGAAGTTCTTGAAGCTCTTGTGTCTATAATAAACACAGATACCTTTTAAGAAATGTGACTTTAAAAATCTGTTTCAAATATATTATGCAGTGGCATGTAAA
CTGATAATTGAATGTAATCATATGCATCTGCTCCAACTGATTGAGGCAGGAATCAACACTAGAACTAGACAAACACAATAAGATTAGACAAGAGATAGG
TGGTCACACTAGAGATAGCTGTACAGGACAGCCTATCTAATAGAGCACACTGGAGGATGGCTCCCCAACACATACACACATAAATGCAGGTGATTTAAG
ATTAGGTTTCAATCTTATTATTTTCTGTAATGATATCTAGTTGGAATAATACCACTTATTAAGAACTCCATCCTTATCCACCTAATTTGAAATGGCACCCTTA
TCAAATATTTTATCAAATGTCTATATTCAAATACCAAAACACAACCCAAACACACCCAACACAAATC

97

TTGTGTTGGGTGTGTTTGGCTTGAGATATGGAAGCATGTCACATCTCCTGGCACTTGTGGACTAGGGTTTGTCACCAATCCTCACCTCTGTTAAATACAT
GGTGCATTGTAGAATTCCACGGTTGTTTGTCTGTGAGGATGCATTAACGATAAAAGTAAGGATGATCAGCAAACCCACATTTCAACAAATCCTTAATCA
ATAACCCCCACCATTAACTGAACACTCACTGTGTACGACGCATGGTTCTAAATGCTTTACACATTTTAACTTATTTTGTCTTCATAAGGAAACTATGATAT
AGGCGCTATTCTTTTCGTCCACTTTTTACACACGAGAAAATCAAAGCACAGAGAAGTTAAATCACTTGTCAAGATCATACTGCTAGTTGGAGGCAAAG
CCAAACATTTGAACCCAGGTGTTACGGCTTCCGAATCCCTGAGCTTAATCATTATATTCCAAAAACCACCAAACACACC

98

TTGTGTTGGGTGTGTTTGGCTTGAGAGGTGGGAGCATGTCACAATGTCCTGGCAACTTGTGGACTAGGGCAGGTCATACAATCCTCAAACTAATGTTAA
ATACATGGTTCATTGTAGAATTCCACAGTTATTTGTCTGTGAGGATGCATTAAGGATAAAAGTCAGGATGATCAGCAGACCCACAACACAACAAATCCAT
AATCAATAACAGCCAACTTTAACTGAACACTCACTGTGTACAACGCATGGTTCTAAATGCTTTACACATTTTAACTTATTTTGTCTTCATAGGGAAACTAT
GATATAGGCGCTATTCTTTTCATCCACTTTTTACAGATGAGAAAATCGAAGCACAGAGAAGTTAAATAACTTGTCAAGATCATACTGCTAGTTGGAGGCA

GAGCCAAACATTTGAACCCAGGTGTTACGGCTTCAGAATCCCTGAGCTTAATCATTATATTCCAAAAACCACCAAACACACCCAACACAAATC
99
TGGGGCAGAGTCGGCCGGCGACGAGAAAGAGATGGGCGTAACAACAATATCCAAATTTAAAACAATAACGTTGAATGATAGACCTTTCACATAAAATT
CCACATAAATTTTATGCTTTATTTAAGTCTCCCTGAGCTGGAA
100
AGGAAGGTCTCTTGTGTTTTTTTTGTTGTGCTTTTTAGGTTCCCTTTATTTTTTTTTTTGTCCTTTCAACCTCTCCCCCTACCCCTCTTGGGGCCCCGCG
101
TTAATTGATAAGTTTATTGAAGTAAAGAAGGAACAACAAAAAAAAACAAAAAAAATAAAAAAAGAAATTTCAACACATATCCAATCAACCCAAACAA
AGAATGAACAAAAAACAGACTGGCAACCCATAC
102
AAGGACCAAACAAACACAAACAAACACACTCGGGAGTACCTGTCTCCTGTACCACTAAAAACGCCCACCACAACTCTGTAGGGGATCCCCTTCTACG
GACCTTTAATTCATAAGAATGAACATTGCTGCTTTCTGTCTAATGTTGGCCCACGCTCACTGCTCCTCATCACATCCCATCTCTACTCTTAAGGGTCCAGA
CTGTGGTGGCTAATGTGTGAGCTATGTGATGTTTCTTGCGTTATCCTGAATGCACTGCACGGAGCCTGAA
103
TTTTTTTTATTTTTTTTTTTTTTTTTTTTTTTTTTTTTTTTTTTTTTTTTTTTAATTAATTAATTTTTTTTTTTTTTTTTTTTTTTTTTTTTTTTTATTTTTTTTTTT
TTCCAAAAACCCCCAAAACAAAACCCCAAAAGAACCCCGGGGACCCAGCCCCAATTCCTAAACCGGGGCAAAACTGTTTCCTGGGGGAAATTGTTAT
CCCCCCCCAATTCCCCACAAAAAACAAACCGGAAACATAAAAGGGAAAACCCGGGGGGCCCAAAGAGGGAGCTAACCCCCATTAATTGGGTTGCGC
CCCCTGCCCCCTTTCCAGTCGGGAAACCTGGCCGGCCCCCTGCATTAATGAATCCGCCAACCCCCGGGGAAAGGGGGTTTGCGTATTGGGGGCCCTTC
CCCTTCCCCCCTCACTGAATCCCTGCCCTCGGGCGTTCGGCTGCGGGGAAGGGTATCAACTCACTCAAAGGGGGGAAAACGGTTATCCCCAAAATCCG
GGGAAAACCCAGGAAAAAACATGGGAGCAAAAGGGCCGCAAAAGGGCCGGAACCCTAAAAAGGGCGCGTTGCTGGGGTTTTTCCCTAGGGTCCCCC
CCCCTGAAAAACATCACAAAAATCCACGCTCAAGTCAAAAGTGGCCAAACCCCACAGGGCTAAAAAGAAACCCGGCGTTTCCCCCTGGAAACTCCCT
CCTGCGCTCTCCTGTTCCCACCCTGCCCCTTACCGGATACCTGGCCCCCTTTCCCCCTCGGGAAACGGGGCGCTTTCTCCTAACTCACGCTGTAGGTATC
TCAGTTCGGGGGAAGTCGTCCCCTCCAAGCGGGGCTGTGGCACGAACCCCCCGTTTAACCCGAACGCTGCGCCTTATCCGGTAACTATCGCCTTGAG
TCCAACCCGGAAAAAACGACTTACCCCACTGGGAGCACCACTGGTTACAGGATAACAGAGCCAGGTATGTAGGCGGGGCTACAGAGTC
104
TCCCCAACCCCCCCAAAAAAAATTTTTAAAGGATCCCCGGGGACCCAGCTCAAATTTTAAATCTGGGGCATAGGTTTTTTCGGGGGGAAATTTTTTTCC

CCCCCCAATTCCCCCCAAAAAAAAAAACCGGAAACAAAAAGGGTAAAACCGGGGGGGCCCAAAGGGGGGGCTAACCCCCATTAATTGGGTTGGCCCC
CCGGCCCCCTTTCCCATCGGGAAAACTGGCGGGCCCCCTGCTTTAAAAAAACCCCCAACCCCCGGGGAAAGGGGGTTTGGGTATTTGGGGCCTTTCCC
CTTCCCCCCCCACTAAACCCCTGGGCTCGGGCTTTCGGGGGGGGGGAGGGGTTTTCCCCTACCTAAAGGGGGGAAAACGGTTTTCCCCAAAAACCGG
GGAAAACCCAGGAAAAAAAAATTGGAGCAAAAGGCCCCCAAAAGGCCCGAAACCCTAAAAAGGGCCCGTTGGGGGGGTTTTTAAAGGGTCCCCCCC
CCTGAAAAAAATCAAAAAAATCCCCCCCAATCAAAAGGGGGGAAACCCCAAGGGAAAAAAAAACCCGGGTTTCCCCCCGGAAACTCCCCCGGGGGC
TCTCTGTTTCCAACCTGGCCCTTACGAGAACCTGTCCCCCTTTTTCCTTCGGGAAACGGGGGGCTTTTTCAAACTCCCCCTGTAGGTATCTCAATTCGG
GGGGAGGCGTTCCCCCCAGTGGGGTGGGGGAAAAACCCCCGTTAACCAACCGGTCCCCTATTCGGAAATTTCTCTTTGGGTCCAACCGGGAAGACAC
ACTTTATCCCACTGGGCGCACCCACGGGAACAGGATAACCACGAGGGTTTTAGGCGGGGTCAA
105
TTGTTTTTTTTTATATAATGGCTTAGATGATTTATATGTACTCCTCTATCGTTCTTATTCTAAGAAATCCAGCGGACCCGCCCGATCTGTTACGAATCCCCGG
TTCCAGAGGAATTATCAGAAAAGTGTCGAAATGAGGGGGCGGGGGTAAAAGTCCTATTATTGAAATGTTAATGGGAGGGCCTGATAAATAAAAGGAGG
AAGATATGAGTGAAGCTACCTGTTTCATTCACCGCTGTATTACGGTGTGCCTAATCATTTTATTTTCCTGAGGAAAGAAACTGATACGGCCAACGCAATA
ATGGTTAAAGACCTTCGGCGAAATCGAGTCGGCCTACCGAACGAGATACCCTTACTCCAAAAACAACCAACAGAA
106
TTTTGGTTATTTCTTTCTATTTACCCAACTCACCAAAATTTTTTCTATAATTCTCTATATTATTTTTTTTTTTTAATGGGAAATCATACCGATGGTCTTTTTGA
TGACCCCCGAATGTGTAAACCGCACACTTATGACAGAACATAGGATGCGGAAGCGGAAAATGGATAGCCTGGCGGGCCTAAAGAGCGTGTCATTCCCA
TTAATTGAATTGCAAAACCTGACTGCCCTCT
107
GCAGCTTGCATGCCTGCAGGTCGACGATTTGTGTTGGGTGTGTTTGGGGGGGGGGTGGGAGTGTTAATGTCACCTCTGAGAAATCTGCCAGGCCTAAAC
AGCAGGCTTCACTCACTTTCACCCTTTGCCCTCAGCCCACACTACTGTCCTTGTCACCCAGCAACTGCCTCGCTCTGAGTCCCATCTCCTGCGTATAGT
GCCACGGCACGAAAAGGACAGGCAGGACCAGGCTCTGCTGCTCCTCTCCTGTGCTGGTGCACCTGTGCACACGTGGCAGCCCACTAGGCTGTGCTCC
AGCCTCCCCACACCCATCTTGCTCATGCACACGACTTCACCAACACACCAAACACACCCAACACAAA
108
ACCAAAAACCCCCCAAAAAAATCTCTAAAGGATCCCCGGGGACCCAGCTCCAATTTTTAAACCTGGGCATAGTTTTTTCCTGGGGGAAATTTTTTTCCC
CCCCCAATTCCCCCCCACAAACCAACCCGAAACAAAAAGGGGAAAGCCGGGGGGGGCCAAAGAGGGAACTAACCCCCATTAATTGGGTTGCCCCCC
CCGCCCCCTTTCCAGTCGGGAAACCTGTCGGGGCCCCTGGTTTAATAAAACGGCCAAACCGCGGGGAGAGGGGGTTTGGGTTTTGGGGCCTCTTCCC

CTTCCCCCCTCAATGAACCCCTGCCCCCGGGCGTTTGGGTGGGGGGAGGGGTTTCAACTCCCTCAAAGGGGGGAATACGGTTTTCCCCAAAAAACCGG
GGAAAACCCCGGAAAAAACATGGGAACAAAAGGCCCACAAAAGGGCAAAAACCCTAAAAAGGGCCCGTTGCTGGGGTTTTTCCATAGGCTCCCCCC
CCCTGAAAAGAATCACAAAAATCCACCCTCAAGTCAAAGGTGGCGAAACCCGAAGGGCTTTAAAGATACCCGGGGTTCCCCCTGAAACTCCCTCGTG
GGCTCTCCTGGTCCAACCTGGCGCTTTCCGGAAACCTGTCCCCCTTTTTCCCTGGGGAAGCGGGGGGCTTTCTCCTAACTCACGCTGGAAGGATCCAA
TTCGGTGGAGGGCCTTTGCCCCAAGCTGGGGTGTGTGCAAAAACCCCCGTTTAGCCGACAGCTGCGCTTTATTCGGTACTATCCTCTTGGGCCACCGGT
AAAACACACTTTTTCCCCTGGGGCACCCCTTGTAACAGGTTAACAAAACGGGGTATGTAGGGGGGCTAAAA

109

AAAAAAACAAACCCCCCCAAAAAAAAATTTCCGAGGGGCCCCGGGGGCCCCCCCTCAATTTTTAAATGGGGGGATATGTTTTTTCGGGGGAAAAATTTT
TTCCCCCCCCAATTCCCCCCACAATAAAACGCGGGAAAAAAAAAGGTAAAACCGGGGGGGGCCAAAGGGGGGGGAAACCCACAAAAATTTGGGGGC
CCCCCCCGCGCCTTTTTCCGGGGGGAAAACGGGGGGGGGCCCCCCTATTAAAAAACGCCCCCCCCCGGGGGGAGGGGGGGTTTGTTTTTGGGGGTTT
TTCCCTTTTTCCCCAAAAAAAAACCCCGGGCCCCGGGCTTTGGGGGGGGGGGGGGGTTTTCCCTCCCCCCAAGGGGGGAAAAAGGGTTCCCCCCAAA
TAGGGGGGATACCCGGGGAAAAAATTTTGTAAAAGGGGGCCAAAAAGGCCCAAAAGCCCAAAAAGGGGCCTGTGGTGGGTTTTTCCCTCCTGCCCCC
CCCCCGTAAGAACACAAAAAACCCCCCCTCCAACCAAGAGGGGGGAAACCCCCCGAGCATTAAAAATACCGGGGTTCCCCCCAGAAAACCACCAGG
GGGCTCCCGTGTCCCCCTAGGCAATAAAAAACCCCCACCTCTTTTCCTTCGGAAGAGAGGGCGTTTTTATAACCCACGCGGGGGTATCAAACGGGGGA
GGTGCTCCCCCCAGGTGGTTGGTAGAAACCACGTTATCCCACGCTGTGCTATACCGGAATATGTGTTAACACACCGGAGAGAACATTTTACCCCCTGGT
ACCCTGTGTAAAAAAAA

110

TCCAGCTTGCATGCCTGCAGGTCGACGATTTGTGTTGGGTGTGTTTGGTTTTTTTTTTTTTTTTTTTTTTTTTTTTTTTTTTTTTTTTTTTTTTTT
TTTTTTTTTTTTTTTTTTTTTTTTTTTTTTTTTTTTTTGCCCAAACCCCCCCCACAAAATTTTTTAAGGAACCCCGGGGCCCCCCCCCCAATTTTTAAACCGG
GGCAAAATTTTTTTCTGGGGGAAAATTTTTTCCCCCCCCAATTCCCCCAAAAAAAAAAACCCGGAAAAAAAAAAAGGTAAAACCCGGGGGGCCCAAAAG
GGGAACCAAACCCCATTAATTTGGTTGGCCCCCCCGCCCCCTTTTCCGCCGGGAAAACTTTTGGGGCCCCTTCTTTTAAAAAACCCCCACCCCCCGGG
GAAAGGGGGTTTTTGTTTTGGGGGCCTTTCCCCTTCCCCCCCCCAAAAACCCCTGGCCCCGGGGTTTTGGGTGGGGGGGGGGGTTTTTCCCTCCTCAA
AGGGGGGATAACGGTTTTCCCCCAAAACCGGGGGAAACCCGGGAAAAAAAAATTGGGTAAAAAAGGCCCCAAAAGGGCCGAAACCCTAAAAAGGGC
CCGTTGGGGGGGTTTTTCCATAGGGTCCCCCCCCCTTAAAAAAATTAAAAAAATCCCCCCCTCATTCCAAAGGGGGGAAACCCCCCCGGAATTAAAAA
ACCCGGGTTTCCCCCCGGAAAACCCCTTGGGGCTTCCTTTTTGAACCTGCCCTTAAAGGAAACCGGCCCCCTTTCTCCCTTGGGAAGGGGGGGCCTTT
TTTCAAATACCCCCGGGGGAACTCCTTTGGGGGAGGGGGTCCCCCACAGGGGGGGGGGGGCGAAACCCCCTTTACCCACAGTGGGCCTTTTCGGGAA

TATGTCTGTGTCACACCCGGAAAAAAACAATTTATCCCCCGGGACACCCTGTAAAAGGATATATAAACAGAGATTTAGGGGCGTACAATTCTTAAA
111
CACCTCAGGGAATGCTGTGGACTTCTTTCCGGTCCTGCGCTACCTGCCCAACCCAGCCCTCAAGAGGTTTAAGAACTTCAATGATAACTTTGTGCTGTT
TCTGCAGAAAACAGTCCAGGAACACTATCAAGACTTCAACAAGAACAGTATCCAGGACATCACAGGCGCCCTGTTCAAGCACAGTGAGAACTACAAA
GACAACGGTGGTCTCATCCCTCAGGAGAAGATTGTCAA
112
GATGAGTTCGTGTCCGTACAACTGGCGTAATCATGGCCCTTCGGGGCCATTGTTTCTCTGTGGAGGAGTCCATGACGAAAGATGAACTGATTGCCCGTC
TCCGCTCGCTGGGTGAACAACTGAACCGTGATGTCAGCCTGACGGGGACGAAAGAAGAACTGGCGCTCCGTGTGGCAGAGCTGAAAGAGGAGCTTG
ATGACACGGATGAAACTGCCGGTCAGGACACCCCTCTCAGCCGGGAAAATGTGCTGACCGGACATGAAAATGAGGTGGGATCAGCGCAGCCGGATAC
CGTGATTCAA
113
CCAAAACCCCCCAAAAAAATCTCTAAAGGATCCCCGGGTACCCGGCCCTAATTTTTAAACAGGGGCATAGTTTTTTCCGGGGGGAAATTTTTTTCCCCC
CCCAATTTCCCCCAAAATAAAAGCCGGAAACAAAAAGGGTAAAACCCGGGGGGGCCAAAGAGGGAGCAAACCCCCATTAATTGGGTTGGGCCCCCC
GCCCCCTTTTCCCTCGGGAAACCTGTGGGGCCCCCTGTTTTAATAAAACCCCCAACCCCCGGGGAAAGGGGGTTTTGGTTTTGGGGGCCCTTCCCCTT
CCCCCCCCAAAAAACCCCTGCCCCCGGGCTTTTGGGGGGGGGGAGGGGTTTCACCTCCTCCAAAGGGGGGAAAACGGTTTTCCCCAAAATCTGGGGA
AAACCCAGGAAAAAAAAATGTTGGCAAAAGGCCCCCAAAAGGGCCGAAACCCTAAAAAGGGCCCGTTGTTGGGGTTTTTTCAAAGGCTCCCCCCCCC
TGAAAAAAATCAAAAAAATCCACCCTCAAGTCAAAGGGGGGGAAACCCCCCAGGGCTATAAAAATACCCGGGGTTTCCCCCTGGAAACTCCCTCGTG
GGCTCTCCTGTTCCCCCCTGCGGCTTAACGGAAACCTGCCCCCTTTTTCCCTTTGGGAAGAGGGGGGTTTTTCAAAACTCACGCTGTAGGTATCTCATT
CGGGGTAGGGCGCTCCCCCCAAGTGGGGTGTGTGAAAAAAACCCCCGTTTGCCCGACGCTGGGCTTTATATGGAAAATATCGTCTTGAGTCCACCGGG
AAGAAAACACATTTTATCCCTGGGAGCACCCTCTGTGACAAGATTACCCCCCGAGGATTTTGGCGGGGGTCTCA
114
ACCCAAACCCCCCCAAAAAAAATCCTAAAGGATCCCCGGGGACCCGGCCCCAATTTTTAAATTGGGGCAAAATTTTTTTCCGGGGGAAAATTTTTTCC
CCCCCCAATTCCCCCAAAAAAAAAAACCGGAAAAAAAAAAAGGGAAAACCGGGGGGGGCCAAAAGGGGGAACAACCCCCATTAATTGGGTTGGCCC
CCCCGCCCCCTTTTCCAGGGGGAAAACTGGTGGGGCCCCTTTTTTTAAAAAACCCCCCACCCCGGGGGGAAGGGGGTTTTGGTTTTGGGGGCCTTTCC
CCTTCCCCCCCCAAAAAACCCCTGCCCCCGGGCTTTTGGGGGGGGGGAAGGGTTTCCCTCCCCAAAGGGGGATTTAGTTTTTCCCAAAACCGGGGTA
ACCCGGAAAAAATTGGGAAAAAAGGGCCAAAAAGGGCCAAAACCTAAAGGCCCGTGGGGGGGTTTTTCTAAGGCCCCCCCCCTGAAAAAATAAAAA

ATCCCCCCCAATCAAAGGGGAAACCCCGGATATATAGTACACGCTTTCCCCCGAAGCCCCCGGGGCTTCCTGTCCAACTGCGTTACGAAACATTCGCC
TTCCCCTTTGGAAGTGGCCTTCCAACTCACGGTGAGTATCTGTCGGAAGCTCGTCAAGTGGCTGTGACAACCTGTACCACGTTGCTTCGGAATTCTGGT
CCACGTAAGACACTATCCCTGGCACCTGTTATGTTCC

115

ACTAGTGCAAAGCCCCTTTACAGAGTTGTCTTTCAAGTGAAAAAATTAATGTAGTTAAAATTATTTGTTTAAAAATTCAATATATGTACAATGCAAAAA

116

TTTTTTGTGGACTGTCACCAAACAGATCTCCTGGCATGTAGACCTCAGGTAAGGGGAACTAAAGACTGAACTCTGACTGCCTGTTCTTTGTTCTATGTA
TTTTTTTGAGGGACCTAGAGGAGGTCATGCTCACGAGCCACAGCTAACATTCTTTTCTGTTAACCCCAAATTTTTATTTATTTATTTATTTATTTATTTATTT
ATTTATTTATTTATTTATTTTTGAGACAGGGTGTTCCTCTGTCACCTAGGCTGGAGTGCAGTGGCGTGATCTCCGCTCACTGCAACCTCTGCCTCCTGGGT
TCATGTGATTCTCTTGCCTCAGCCTCCTGAGTAGCGAGTAGCTGGGACTACAGGCATGCAGCACCACACCCAGCTAATTTTTGTATTTTTTGGTGGAGA
CGGGGTTTCACTGTGTTGGCCATGCTGTTCTCTAACTCCTGGCTTCAAGTGATCTGCCCACCTTGGCCTCCCAAATTGCTGGGATTATAGGAGTGAACTA
CTGCACCCGGCCTGACCCCAAATTTTTAAACAAAAACAACCAAACACACCCAACACAAATCGTCGACCTGCAGGCATGCAAGCTTGGCACTGGCCGT
CGTTTTACAACGTCGTGACTGGGAAAACCCTGGCGTT

117

GGTGTTTTGGGCACAAATTCTGACTTCAAATCCAGCGGCCTTTTTCTCACATAATACATGGAGGAAAAAACGTCTGTGGCCTTGAATTTCTTTCTCTCAT
AAGTCTTACTGCCACATGACCTCAAAATGAACCAACCATGCATCATATCTCCCTTTTTCGTAGTTCTAACCAGACCCTCTCAGCCAAAGAAGATCCCATT
CCCAGCTGGAAATGCTGTCTCGGAGCAGGAATGAAGAGCCTCTCTCATTGGAATTGCCCATGAGGACAACACCCCCCC

118

TTTTTTGTGGACTGTCACCAAACAGATCTCCTGGCATGTAGACCTCAGGTAAGGGGAACTAAAGACTGAACTCTGACTGCCTGTTCTTTGTTCTATGTA
TTTTTTTGAGGGACCTAGAGGAGGTCATGCTCACGAGCCACAGCTAACATTCTTTTCTGTTAACCCCAAATTTTTATTTATTTATTTATTTATTTATTTATTT
ATTTATTTATTTATTTATTTTTGAGACAGGGTGTTCCTCTGTCACCTAGGCTGGAGTGCAGTGGCGTGATCTCCGCTCACTGCAACCTCTGCCTCCTGGGT
TCATGTGATTCTCTTGCCTCAGCCTCCTGAGTAGCGAGTAGCTGGGACTACAGGCATGCAGCACCACACCCAGCTAATTTTTGTATTTTTTGGTGGAGA
CGGGGTTTCACTGTGTTGGCCATGCTGTTCTCTAACTCCTGGCTTCAAGTGATCTGCCCACCTTGGCCTCCCAAATTGCTGGGATTATAGGAGTGAACTA
CTGCACCCGGCCTGACCCCAAATTTTTAAACAAAAACAACCAAACACACCCAACACAAATCGTCGACCTGCAGGCATGCAAGCTTGGCACTGGCCGT
CGTTTTACAACGTCGTGACTGGG

119

GTGTTTTAGCCCTAGCCAGTCAGTTGGCCAAAGTCTACCTCTGACTCAGCACATATATAATAAATGCTCAATGCTTAATGAGCATGACCACCACAAATAA
ATGAGGGTCAATGAACAGAGCATTAAAGTATAGGTCAGCAAGTCTTAGTTCTTCAGGATAAATATTACACTAAAAAGAAAATAACCTGTTTCTTCCACTT
AAAAAATGTGATGTAGTAAGTGTAGCCATAAAGATAAATGAATTAGTAAATATTGTAATCACAGCTATGACTAATTTTATAGCACTTCTAGATTACAAAAA
ATTTTTCCCAATAGAATATATAC

120

TGGTGTGTTTAAGAAAGTAAAATGGAAAGCGGGGGTAGGGTGAGGGGTCATTCTGATGATATAGTTTTAGGACCTGGATGTAGCCATACCTGTAGCTGT
CAACTCTGTGCCATAGTACTGCTTTTTTTTTTTTTTTCCTTCAAATTTAAATACTTTCTAAAGGCAAGGTCTTGCTATGTTGCTTAGGCTGGTTTTGAAAA
GTATTTAAATTTGAAGAATCGTCGACCTGCAGGCATGCAAGCTTGGCACTGGCCGTCGTTTTACAACGTCGTGACTGGGAAAACCCTGGCGTT

121

GTGTTTTAGCCCTAGCCAGTCAGTTGGCCAAAGTCTACCTCTGACTCAGCACATATATAATAAATGCTCAATGCTTAATGAGCATGACCACCACAAATAA
ATGAGGGTCAATGAACAGAGCATTAAAGTATAGGTCAGCAAGTCTTAGTTCTTCAGGATAAATATTACACTAAAAAGAAAATAACCTGTTTCTTCCACTT
AAAAAATGTGATGTAGTAAGTGTAGCCATAAAGATAAATGAATTAGTAAATATTGTAATCACAGCTATGACTAATTTTATAGCACTTCTAGATTACAAAAA
ATTTTTCCCAATAGAATATATACACCAAAAACCAAACACACCCAACACAAATCGTCGACCTGCAGGCATGCAAGCTTGGCACTGGCCGTCGTTTTACAA
CGTCGTGACTGGGAAAACCCTGGCGTT

122

TGGTGTGTTTAAGAAAGTAAAATGGAAAGCGGGGGTAGGGTGAGGGGTCATTCTGATGATATAGTTTTAGGACCTGGATGTAGCCATACCTGTAGCTGT
CAACTCTGTGCCATAGTACTGCTTTTTTTTTTTTTTTCCTTCAAATTTAAATACTTTCTAAAGGCAAGGTCTTGCTATGTTGCTTAGGCTGGTTTTGAAAA
GTATTTAAATTTGAAGAATCGTCGACCTGCAGGCATGCAAGCTTGGCACTGGCCGTCGTTTTACAACGTCGTGACTGGGAAAACCCTGGCGTT

123

TGGTGTGTTTAAGAAAGTAAAATGGAAAGCGGGGGTAGGGTGAGGGGTCATTCTGATGATATAGTTTTAGGACCTGGATGTAGCCATACCTGTAGCTGT
CAACTCTGTGCCATAGTACTGCTTTTTTTTTTTTTTTCCTTCAAATTTAAATACTTTCTAAAGGCAAGGTCTTGCTATGTTGCTTAGGCTGGTTTTGAAAA
GTATTTAAATTTGAAGAATCGTCGACCTGCAGGCATGCAAGCTTGGCACTGGCCGTCGTTTTACAACGTCGTGACTGGGAAAACCCTGGCGTT

124

GTGTTTTAGCCCTAGCCAGTCAGTTGGCCAAAGTCTACCTCTGACTCAGCACATATATAATAAATGCTCAATGCTTAATGAGCATGACCACCACAAATAA
ATGAGGGTCAATGAACAGAGCATTAAAGTATAGGTCAGCAAGTCTTAGTTCTTCAGGATAAATATTACACTAAAAAGAAAATAACCTGTTTCTTCCACTT
AAAAAATGTGATGTAGTAAGTGTAGCCATAAAGATAAATGAATTAGTAAATATTGTAATCACAGCTATGACTAATTTTATAGCACTTCTAGATTACAAAAA

ATTTTTCCCAATAGAATATATACACCAAAAACCAAACACACCCAACACAAATCGTCGACCTGCAGGCATGCAAGCTTGGCACTGGCCGTCGTTTTACAA
CGTCGTGACTGGGAAAACCCTGGCGTT

125

GTGTTTTAGCCCTAGCCAGTCAGTTGGCCAAAGTCTACCTCTGACTCAGCACATATATAATAAATGCTCAATGCTTAATGAGCATGACCACCACAAATAA
ATGAGGGTCAATGAACAGAGCATTAAAGTATAGGTCAGCAAGTCTTAGTTCTTCAGGATAAATATTACACTAAAAAGAAAATAACCTGTTTCTTCCACTT
AAAAAATGTGATGTAGTAAGTGTAGCCATAAAGATAAATGAATTAGTAAATATTGTAATCACAGCTATGACTAATTTTATAGCACTTCTAGATTACAAAAA
ATTTTTCCCAATAGAATATATACACCAAAAACCAAACACACCCAACACAAATCGTCGACCTGCAGGCATGCAAGCTTGGCACTGGCCGTCGTTTTACAA
CGTCGTGACTGGGAAAACCCTGGCGTT

126

GACATGACATGATTACGATTCGAGCTCGGTACCCGGGGATCCTCTAGAGATTTGTGTTGGGTGTGTTTGGGTTGTTGGGCCATGACGATCCTTTGGGGC
CAGTCTACCTCTGACTCCTCACATATATAATAAATGCTCTGTGTTTAATGAGCATGACCACCACAAATAAATGAGGGTCAAAGAACAGAGCATTAAAGTA
TAGGTCAGCAAGTCTTAGTTCTTCAGGATAAATATTACACTAAAAAGAAAATAACCTGTTTCTTCCACTTAAAAAATGTGATGTAATAAGTGTAGCCATA
AAGATAAATGAATTAGTAAATATTGTCATCACTGCTATGACTAATTTTATAGCACTTCTAGATTACAAAAAATTTTTCCCAATAGAATATATACACCAAAAA
CCAAACACACCCAACACA

127

TGGTGTGTTTAAGAAAGTAAAATGGAAAGCGGGGGTAGGGTGAGGGGTCATTCTGATGATATAGTTTTAGGACCTGGATGTAGCCATACCTGTAGCTGT
CAACTCTGTGCCATAGTACTGCTTTTTTTTTTTTTTTCCTTCAAATTTAAATACTTTCTAAAGGCAAGGTCTTGCTATGTTGCTTAGGCTGGTTTTGAAAA
GTATTTAAATTTGAAGAATCGTCGACCTGCAGGCATGCAAGCTTGGCACTGGCCGTCGTTTTACAACGTCGTGACTGGGAAAACCCTGGCGTT

128 号

TGTTTTTTTGATAGGAAGAATTACTTTTTAAGAGAAAGAGCTACGCACCATTTGGGTAGGATCTGAGGATTTACATAATTTCAATATATAAACAGCCTATA
TTCATCTCATCAAGAATAGATATAGTCTGAGAAAAAAGAACAGAACACTCAGTAGTCTGCAGTTTAACTGTTAGATTCAGTATCTTTGCCCATGTATAGC
CAACAACCCCCAAACACACCCAACACAAATCGTCGACCTGCAGGCATGCAAGCTTGGCACTGGCCGTCGTTTTACAACGTCGTGACTGGGAAAACCC
TGGCGTT

129

TGTGTTGGGTGTGTTTGGTGGTTTTGGTAAAGACAGGGTTTCACCCTGTTGGCTTGCTGGTCTCTAACTCCTGGCCTCAAATGATCCTCCTGCCCCACC

AACGCCAACAAGTTGACGGCTTTCCGTATGGATACACCCGGGGACTAAAACCATATCAATAGACTGACCCCTCACAGTTGCCCCGGTTTCATTTTTATTT
TCTTAAACCCCCCCCCAAAAACAGCCACCACA
130
GGTTTTGTTATATTCCATTCCATTCCATTCCATTCCATTCCATTCCATTCCATTCCATTTCATTCCATTCCATTGCATTCCATTCCTCTCACGTTGTTTCCATT
CCGTTCCATTAGTTTCCATTCCATTCCATTCCTTTCCATTCAACCCAACCCCAAACACACCCAAC
ACAAATCGTCGACCTGCAGGCATGCAAGCTTGGCACTGGCCGTCGTTTTACAACGTCGTGACTGGGAAAACCCTGGCGTT
131
CTTGACATGATTACGATTCGAGCTCGGTACCCGGGGATCCTCTAGAGATGTGTTGGGTGTGTTTGGGTGGTTGTTGAATACAGTCAATAGACTTTTGGAT
GTTTTCACAGGGCTGAAGCTTTGTGCAGGGTCTTTATTTATATCTGACTTCTGGTCTTTGGTTTTACAGCAGGGATATGTTAGTGAGGCATTTTTGGTGTA
AAACCAAACACACCCAACACAAATCGTCGACCTGCAGGCATGCAAGCTTGGCACTGGCCGTCGTTTTACAACGTCGTGACTGGGAAAACCCTGGCGT
TACCCAACTTAA
132
AATTTGACATGATTACGATTCGAGCTCGGTACCCGGGGATCCTCTAGAGATTTGTGTTGGGTGTGTTTGGTCATTCACCTAAATAAGCCCTGATTATTCCA
ACTCTGTATGACCGAGCAGTCAGAAAAAAAGAAATGTCAGTTGTGGCTGGAGAACCTGTTGCTAAAATGCCAGGATCCACGGAAGAAAGGAGACACA
TGGGATCAACACCCACCACCCAAACACACCCAACACAAATCGTCGACCTGCAGGCATGCAAGCTTGGCACTGGCCGTCGTTTTACAACGTCGTGACT
GGGAAAACCCTGGCGTT
133
GGCCCTTGACTGATTACGATTCGAGCTCGGTACCCGGGGATCCTCTAGAGATTTGTGTTGGGTGTGTTTGGGTGGGGGTTTAGTTGGCTACACTGGTGT
TCCCAGGGTCTCCTTCTCTGTATGGTTCTGAGTTAAAGTTGGGCAAAAGAACTTGCATGAGATTTGGAAGAGGAGTCATTTCTCTGAAAGTCATTTGAT
GGTAAGATGAGATGACCAACAGACGCAGAGTTGCCCAGCAGTTTCCAGCTTGTTCTCACTCTCCCTCTCTGTGACCAGTCTGGCCCCACTGACCAACA
GTGGCCCCTCCCCAACACCAAATTATTGGTTGTGAATTGACACAGAGGGTAGCTACCTAGAGGCAACAGCTTCCCATGGATGCCTCTGCCTCAGTGACT
AGATAGAGTTGGCTCTTTGGATGGACAGGTGGGTGACTCTTTCTCTGATTCTCTAACTTCCTGTTCTGGATCTTCAGTTCCCCAGCTCTTTTGTAAATTCT
AACTCTTTAATCTATGGAATTAAATCTCTTATCCCATCGCAGTTACAGTGACTCTGTTGCTCCCCTGCCTAAGTGTGCCAAGGTTAGGACAAGGTACGTA
AGAACAGCAGAGTCATTCAGGAGCCAAACCCCACCAAACACACCCAACACAAATCGTCGACCTGCAGGCATGCAAGCTTGGCACTGGCCGTCGTTTT
ACAACGTCGTGACTGGGAAAACCCTGGCGTTACCCAACTTAA
134

GGCCATGACATGATTACGATTCGAGCTCGGTACCCGGGGATCCTCTAGAGATTTGTGTTGGGTGTGTTTGGGTGGGGGTTTAGTTGGCTACACTGGTGT
TCCCAGGGTCTCCTTCTCTGTATGGTTCTGAGTTAAAGTTGGGCAAAAGAACTTGCATGAGATTTGGAAGAGGAGTCATTTCTCTGAGAGTCATTTGAT
GGTAAGATGAGATGACCAACAGACGCAGAGTTGCCCAGCAGTTTCCAGCTTGTTCTCACTCTCCCTCTCTGTGACCAGTCTGGCCCCACTGACCAACA
GTGGCCCCTCCCCAACACCAAATTATTGGTTGTGAATTGACACAGAGGGTAGCTACCTAGAGGCAACAGCTTCCCATGGATGCCTCTGCCTCAGTGACT
AGATAGAGTTGGCTCTTTGGATGGACAGGTGGGTGACTCTTTCTCTGATTCTCTAACTTCCTGTTCTGGATCTTCAGTTCCCCAGCTCTTTTGTAAATTCT
AACTCTTTAATCTATGGAATTAAATCTCTTATCCCATCGCAGTTACAGTGACTCTGTTGCTCCCCTGCCTAAGTGTGCCAAGGTTAGGACAAGGTACGTA
AGAACAGCAGAGTCATTCAGGAGCCAAACCCCACCAAACACACCCAACACAAATCGTCGACCTGCAGGCATGCAAGCTTGGCACTGGCCGTCGTTTT
ACAACGTCGTGACTGGGAAAACCCTGGCGTT

135

TGTTAACCTATGTACTTGGGAGGCTGAGGCAGGACAATCGCCTGAAGCCAGGAGGTTGTGGTTGCCATGAGCCAGGACCACACCACTGTCTTCTCTAT
CCTGGGTGACAGAGCAAGACTCCGTCACAAAAAAAAAAAAAAAAGACAAAAAGCAAACTGGGCAGGCCTCCAGACCATGCACATGGACCTGTACCA
AGGTGTTCCCCCCCCTAACTTGACCAAACACACCCAACACAAATCGGCGACCTGCAGGCATGCAAGCTTGGCACTGGCCGTCGTTTTACAACGTCGTG
ACTGGGAAAACCCTGGCGTT

136

TTGGTGTTCCTCTTAAGTGATCTTTCTTCCTCTAGATCTCTCTTCAGCTTTTAAGTTATTTCAAGCTTCTGTAACCCCGACAGGAGACTGCTGCTTGGATA
AGCATATCAGCTGCATGGTTAAACTATTTGTTCTGTCTTTCAACTGAGCTGTCAATACACTCTTTGAAAAATTTGAAGGGAAGAGTATAGTTCCACTTAC
TATTTCTTTATAAGTAACCAAGAAACAACGTAACTTTCATATTAATCTTCGGGAAGGAAGGATTTTTTATTCTATGAATTTTGTGGTAGGATTTTTTGTGGT
AGGATTTCTGGCCTTATTGTAATTTTATGCCTTGGTATTTAAAGGCAAAGTCCAATCCACTGCTGATATCTTTGGTGTCTAAGAAGGACATCTGTTCAAAG
AAAAGGCCGTACCATTGCCAGAAGCCACTGTGAGTCACACCGATGAAAATAAAGCAAGAGCAGAAAAAAAAGGTGAGCAGGAGGGAAGACTTAACG
CAGGAGCATTGATCTGCAGCATTTTAATAATTCATCCGGAACGCAGACGGCAGTACTGTTCCCTTCCACGTTCATATTGAACAACTTCAAACAGAAAGT
GGAAAGAACATTGCAGTGAACACCCAA

137

GTGTTTGGGTTGGTTTGAAATATTTTCATTACAAATTCTTCTCTGACATTGTTTAAAATATTTTCATTACAAATTCTTCTCTGACATTTGTAAAACATTTTCT
ATTTGTTTTACAAATTTTATTTTTAAAAATTAATTTAAAAGCCAATAGTCACTGCAGAGCTTAGAATAAATTTCATCATTTTTCTTTATACACAAAAACCAA
ACACACCCAACACACGTCTATAATCTCAGCTCACTGCAACCTCCGCCTCCTGAGTTCATGGAGTTCTCATGCCTCAGCCTCCTGAGTAGCTGGGATTAT
AGACATACACCACCATACCCGGCTAATTTTTATATTTTTAGTAGATGCAGGGTTTCACCATGTTGGCCAGGCTGGTCTCAAACTCCTGACCTCAAGATCC

ACCTACCTTGGCCTCCCAAAGTGCTGGGATGATAGGCATGACCCCCCCCCAAACACACCCAACACAAATCGTCGACCTGCAGGCATGCAAGCTTGGC
ACTGGCCGTCGTTTTACAACGTCGTGACTGGGAAAACCCTGGCGTT
138
TGTTTGGTTGCATTGTGACTGGAAAGTAGGCCTGGAAAATGGACACAGATTGGCCAGGTGCAGTGGCTCATGCCTGTAATCCCAGTACTTTGAAAGGC
CAAGGCTCTTAAGGTCCTCAAGGATCACTTGAGATCAGGAGTTCAAGACCAGCCTGGCCAACATGGTGAAACCCTGTCTCTACTAAAAATACAAAAAT
TAGCTAGGCATCATGGCAAGCACTTGTAGTCCCAGCTACTCAGGAGGCTGATGCAGGAGAATCACTCGAACCCGGGAAGCAGAGGTTGCAGTGAGCC
GAGATCAAGCCACTGCACTCCACCCCATCTCAAAAAACACCCAAACACACCCAACACAAATCGTCGACCTGCAGGCATGCAAGCTTGGCACTGGCCG
TCGTTTTACAACGTCGTGACTGGGAAAACCCTGGCGTT
139
TGTTGTGGTTCCTATGAACTCTTGGTGTGCTGACCTAAACACATTTGCTGATCCTGAAAGGCTGAGAACCAGATACTGGAACATTGCAAGTTTACCAAT
GGAGAGATAAGAGATGTTGGCGATCTCTGGGTGGTCAGCCTGGGTAATTCAGTCAATTATCTGCCTCATTGGACAATCGTCAAAATGCAAAAGATACAT
TCAATTTGATTTGGTAGCTTTTCTACCCCCAAAAAACCAAACACACCCAACACAAATCGTCGACCTGCAGGCATGCAAGCTTGGCACTGGCCGTCGTT
TTACAACGTCGTGACTGGGAAAACCCTGGCGTT
140
TTTATGCTCATGAAAGATATCTGGGCCAGAATTATAGAGGTGGTGTCACCGCATTTCAGTGCTGGTTGAAGACCCCAAAGCCAATGAAAATTTCCCCAC
ATGCACTGAGTAGAAAGCAGAGATATCCTGGGATAGAATCTTCAGGACTCCAGTATTCAAGGAGCAGCTAGATAAGGAGCCAATAAAGAAAGGTCTGA
GGAGGCCCACAGCCCAGCCTCTTCCACCTTCTTTTTCCATAATAATCTCCATCTTCTTATTTCTACTTCCTTTCTCTGTCCCCCAACCCAAACACACCCAA
CACAAATCGTCGACCTGCAGGCATGCAAGCTTGGCACTGGCCGTCGTTTTACAACGTCGTGACTGGGAAAACCCTGGCGTT
141
TTGTTGGGTGATCGTGTTCAAAATAATAAAATTGGTGCCTGTCTAATGGCAGAACCAAATCTAGTTGCAGAATGCATTCACAGTATGCAAAAAGCCGTC
TCAATTCCTGTCACGGTAAAACATCGTATTGGAATTGATGATATGCAGTCTTATGAAGAAATGCTGCACTTCGTCGATACTGTAGCAGCTACAGGATGTA
CACATTTTATCGTGCATGCACGCATTGCGATCTTGCAAGGTTTGTCACCCAAAAAAAACCACCCAAACACACCCAACACAAATCGTCGACCTGCAGGC
ATGCAAGCTTGGCACTGGCCGTCGTTTTACAACGTCGTGACTGGGAAAACCCTGGCGTT
142
TGTTGTGGTTCCTATGAACTCTTGGTGTGCTGACCTAAACACATTTGCTGATCCTGAAAGGCTGAGAACCAGATACTGGAACATTGCAAGTTTACCAAT
GGAGAGATAAGAGATGTTGGCGATCTCTGGGTGGTCAGCCTGGGTAATTCAGTCAATTATCTGCCTCATTGGACAATCGTCAAAATGCAAAAGATACAT

TCAATTTGATTTGGTAGCTTTTCTACCCCCAAAAAACCAAACACACCCAACACAAATCGTCGACCTGCAGGCATGCAAGCTTGGCACTGGCCGTCGTT
TTACAACGTCGTGACTGGGAAAACCCTGGCGTT
143
GGTGTGTGTTAACCTAGGTACTTGGGAGGCTGAGGCAGGAGAATCGCCTGAAGCCAGGAGGTTGTGGTTGCAGTGAGCCAAGACCACACCACTGTAT
TCTCTAGCCTGGGTGACAGAGCAAGACTCCGTCTCAAAAAAAAAAAAAAAGACAAAAAGCAAACTGGGCAGGCAGCCAGACCATGCACATGGAGCT
GTACCAAGGTGTTCCCCAGCCTAACTTGACCAAACACACCCAACACAAATCGTCGACCTGCAGGCATGCAAGCTTGGCACTGGCCGTCGTTTTACAAC
GTCGTGACTGGGAAAACCCTGGCGTT
144
TGTTCCTCTTAAGTGATCTTTCTTCCTCTAGATCTCTCTTCACCTTTTAAGTTAGTTCAAGCTTCTGTAACCACAACAGGAGACTGCTGCTTGGATAAGC
ATATCAGCTGCATGGTTAAACTATTTGTTCTGTCTTTCAACTGAGCTGTCAATACACTCTTTGAAAAATTTGAAGGGAAGAGTATAGTTCCACTTACTATT
TCTTTATAAGTAACCAAGAAACAACGTAACTTTCATATTAATCTTCGGGAAGGAAGGATTTTTTATTCTATGAATTTTGTGGTAGGATTTTTTGTGGTAGG
ATTTCTGGCCTTATTGTAATTTTATGCCTTGGTATTTAAAGGCAAAGTCCAATCCACTGCTGATATCTTTGGTGTCGAAGAAGGAGATCTGTTCAAAGAA
AAAGCCGTAGCATTGCCAGAAGCCACTGTGAGTCAGACAGATGAAAATAAAGCAAGAGCAGAAAAAAAGGTGAGCAGGAGGGAAGACTTAACGCA
GGAGCATTGATCTGCAGCATTTTAATAATTCATCCGGAACGCAGAGGGAAGTACTGTTCCCTTCCACGTTCATATTGAAGAACTTCAAACAGAAAGTGG
AAAGAACATTGCAGTGAACACCCAAACACACCCAACACAAATCGTCGACCTGCAGGCATGCAAGCTTGGCACTGGCCGTCGTTTTACAACGTCGTGA
CTGGGAAAACCCTGGCGTT
145
TTTTGGTGGGAAATGGGCAACTCCAGTCACACTCAGAAAATGGAAGTGAAAATTGGGGCCAGGATCATCTTAGAAGCACCAACAAAAGCATATTGGT
AAAATACCCAACAATAAAGATCACAAAAATTAGCCACACATGGGATAATCACAACCCCAAACACACCCAACACAAATCGTCGACCTGCAGGCATGCAA
GCTTGGCACTGGCCGTCGTTTTACAACGTCGTGACTGGGAAAACCCTGGCGTT
146
TGTTCCTCTTAAGTGATCTTTCTTCCTCTAGATCTCTCTTCACCTTTTAAGTTAGTTCAAGCTTCTGTAACCACAACAGGAGACTGCTGCTTGGATAAGC
ATATCAGCTGCATGGTTAAACTATTTGTTCTGTCTTTCAACTGAGCTGTCAATACACTCTTTGAAAAATTTGAAGGGAAGAGTATAGTTCCACTTACTATT
TCTTTATAAGTAACCAAGAAACAACGTAACTTTCATATTAATCTTCGGGAAGGAAGGATTTTTTATTCTATGAATTTTGTGGTAGGATTTTTTGTGGTAGG
ATTTCTGGCCTTATTGTAATTTTATGCCTTGGTATTTAAAGGCAAAGTCCAATCCACTGCTGATATCTTTGGTGTCGAAGAAGGAGATCTGTTCAAAGAA
AAAGCCGTAGCATTGCCAGAAGCCACTGTGAGTCAGACAGATGAAAATAAAGCAAGAGCAGAAAAAAAGGTGAGCAGGAGGGAAGACTTAACGCA

GGAGCATTGATCTGCAGCATTTTAATAATTCATCCGGAACGCAGAGGGAAGTACTGTTCCCTTCCACGTTCATATTGAAGAACTTCAAACAGAAAGTGG
AAAGAACATTGCAGTGAACACCCAAACACACCCAACACAAATCGTCGACCTGCAGGCATGCAAGCTTGGCACTGGCCGTCGTTTTACAACGTCGTGA
CTGGGAAAACCCTGGCGTT
147
TCAAATTATCAGGCTTAAGGATCTCTTAGGAAATCATATTTTCCTAAGTAAAGCTCTAGCTAGGGAATATTTTGGATGAAAAATTCCAAAAAAGATATTGT
TTACTCATTATATAATAGGGCTGGTAAAAGCCAAAAATATTGTAGGAATAAGTATGTTCACCATCTCATAAAGACTCAAGTACTGCTTGCCTTTCTTGGAT
CCCCAGGCATAAATCAAATCATAAAATATTTTCCTGACAAGTTCTTAAATCTGTGACTTATAATGAATAGTACAGTTCTACTAGTCTTGCTAAAACATTAG
TAATTAAACACAGATTTCACGTATAAGGTCACTTATTGAAATGCTATATTAACAGCTCCATATTAAAAAAAACCCAAACACCAAACACACCCAACACAA
ATCGTCGACCTGCAGGCATGCAAGCTTGGCACTGGCCGTCGTTTTACAACGTCGTGACTGGGAAAACCCTGGCGTT
148
TTTTTGTGTTTCTGGTAGAATGATTTACATTCCTTTGGGCATATACCCAGTCATGGGATTGCTGGGCTGAACAGCAATTCTGTTTTATCTTCTTTGAGAAA
TCTCCAAACTGCTTCCCACCGCAGCTGAACTAGTTTATATTCCCACCAGCAGTATATAAGCATTCCCATTTCTCCACAGCCTTGCCAGCATCTCTTCTTTA
TGGACTTTCTAATAACAACCACTCTGACTGTTGTGAGATGGTCTCTCATTGTGGTTTTGATTTGCATTTCTCTAAGGATTAGTGATGTTGAGCACATTTCT
GAATGAACCATCCAGCTTGGCTTCATTGTTTTTTTATTGTTTCACCTGAAGAACTTAAAGAAAGAAGTAAAAGAGATATATTTGCCCTTTGATTTATGAAT
GAATGATGATGTCTTAGATCTCAAATATTTGCTAAAAATGTAGATACCTTTAGAGTACTTCTATACAGAAACCAAAATATTAAAATTAACAAATCTGCTTC
CTTCATTAGTATCTCATTCTCCAAGTTTGACATTTGAATCCTAATTCCTAAAAATTGAATGTTTTGGTAAATCATAGTCTGTTCTTTAGACTACATTTCTTTC
CTTCTTAAGAAATAATTGCATATCAATATAGCTATCTGGATGTATTTATCTGTGTAAAGAATGTGTTAGAAGTGTAACCAGATTGCATTTATAAATATTATTG
TAACACCACCACCAAACACACCCAACACACTTGTTGAATCTTCAAATCTATCCAAATGTCCACTTGCAGATTCAACAAAAAGTGTTTTTCAGAACTGCT
CTATCAAAAGAAAGATCCACCTCTGTTAGCTGAGTTCAGACATCACAAACAAGTTTATGAGAATGCTTCTGTCTAGTTTTTATTTGAAGATATTTCCTTT
CTCACCATAGAGCTGAAAGCTGTCCTAATGTTCACTTCCAGTTACTACAGAAAGAGTGTTTCAAAACTGCTGTACGAAAGGGAATGTTCAACTCTGTG
ACTTGAATGCA
149
ACAATGACATGATTACGATTCGAGCTCGGTACCCGGGGATCCTCTAGAGATTTGTGTTGGGTGTGTTTGGGTTTTGACTCGGTCCAATTCATCCCTGCTT
TTATAATTTTCTTTTTTTTGAAGGTGGAGAGGCGGGAAGGTCCCCAGTGTGTTCATGGGAACGCAGGGCATGCCTCACTGCCTTTGGACTTGCCGCTCC
CTGGGTTGGGAGGGAGACCTCTCTGACAGATGCCCACCTTATGTTCTCAGCGACATTTCCCTCACCATTCCCTCTACCCCTTTTTTTTAAAAAAAAAAA
AGAGACATAACTCATATACTGTAAAATTCACCATGTTACGGTGCAGAGTTGGGTGGGTTTCAGCGGATCCACAGGGCAGTGCAGCTGTCACTTCTCTCT

ATGTCTGGAACATTCCCATCACCCCAGAAAGGAACCCTGTTCTCTTAAGCAGCGTCTACCCATTTTCCCCTCCCCCATAATCCTGGCAACCACAAATCC
ACTTTCGGTCTCCATGGATTTGCCTCTCGTTGACATTTCACATAAGCGGGATCATTCTATAAGTGAGCTTTTGTGTCTGGCTTCTTCCACTCAGCATCGTG
TTCTCAAGGTTCATCAGTTTGACCAGGTGTCAGTGCTTCCTTCCTTTTTCTGGCTGAGCAATATTCCGTTGTGATTTAAGGGAACATGAATACACAGCAT
TTTCTTTATTTACTCCTCCATTGATGGACATTGGGTTGCTCCCACTTTTTGGCTCTTATGAATAATGCTGTTATCCCTATTTGTATTCACATTTTTAAATGAA
CACAGGTGTCAGTATCTCTGTGCACATTGTAGTGTGACATCTGGTAACTTCGTGTTTGACATTTCCATGAAGTCTCCACCAAAGCTGCACCATTTTTCCA
TTCCCACCAACAGGCGGGAGAGCTCCCAAAACCAAACACACCACACAAATCGTCGACCTGCAGCATGCAAGCTTGGCACTGGCGTCGTTTACAACGT
CGTGACTGGAAACACTGTCGTACCACATCATCGCGTGTAGCACATCTCCCTTCTCAGCTGCGTATAGCGAGAGACGG

150

GGGATTGGGAGATGGAAGATAGACGAGACAAGAGACCTAGAATCTATTTAGACTCTGACAGTGTCCCAGACACTGGAATGAGGATCTGAAGTAGTAG
GAACAACGGGACAGAGACATTCGGTAATATGTAAGAGCTTCAGCTGGTGTATGTAATATGTAAGAGCCTGGTGATCAGTTTAATGTGACAGAGAAGAG
GGAGGAAGAAATATCAGAATGGGATTTCCATTTCAAGAATATAAGTACTAGGCTCATCACCACCACATGAGATAGAAAACATACACACACACACACAC
ACACACTCACCCACACACCAAACACACCCAACACAAATCGTCGACCTGCAGGCATGCAAGCTTGGCACTGGCCGTCGTTTTACAACGTCGTGACTGG
GAAAACCCTGGCGTT

151

CTCAAAAAATAAATAGCAAAGAAAGACAAGTTCTTCCCAAGCAAAACACAACCCAAACACACCCAACACAAATCGTCGACCTGCAGGCATGCAAGC
TTGGCACTGGCCGTCGTTTTACAACGTCGTGACTGGGAAAACCCTGGCGTT

152

GCGATGACATGATTACGATTCGAGCTCGGTACCCGGGGATCCTCTAGAGATTTGTGTTGGGTGTGTTTGGGGTTTGTTGTCCATCATTAAGTTTAGAGAA
CCCCAAACATTGAAGTAATAACAGCAAAGAGCTTTCCAAAATTAATGATAGACACCAAATCACAGATACAGAAAGTTTAGAGAACCCCAAGCACAACA
AAAACACCAAACACACCCAACACAAATCGTCGACCTGCAGGCATGCAAGCTTGGCACTGGCCGTCGTTTTACAACGTCGTGACTGGGAAAACCCTGG
CGTT

153

GCATGACATGATTACGATTCGAGCTCGGTACCCGGGGATCCTCTAGAGATTTGTGTTGGGTGTGTTTGGTGGTTGTTGGAAATGGGAATATCTTCACATA
AAAACTAGACAGAAACATTCTCAGAAACTTCTTTGTGATGTGTGCATTGAACTCACAGGTTTGAACCTTTCTTTTGATGGAGCAGTTTTTAAACACTTT
TTTTTATTATTATACTTTGTTTTAGCGTACATGTGCATATTGTGCAGGTTTGTTACATATCTATACATGTGCTATGTTGGTGTGCTGCACCCACTAACTCGTC
ATCTAGCATTAGGTATATCCCCCGATGCTATCCCTCCCCAAACACACCCAACACAAATCGTCGACCTGCAGGCATGCAAGCTTGGCACTGGCCGTCGTT

TTACAACGTCGTGACTGGGAAAACCCTGGCGTT

154

GTCAATCACTGCTTTTAAATTCTTAATACTATACTTTTTGAATAAAGGGCTCTGCTTTTTCATTTTGCACTGGGTCCCACAAATTATGGAGCCAGTCCTGG
TTAGGGCAGTGGTTGTGGGAATGGAGGAATGATAAGGATCCATAGGTTGTTAGGAGGTCAAATAGGACTTATTTATTGATTCATTGGATGAAGAGTTAG
GAAGAAGGAGGAACTAGGATAACTTTCTGTTTTCCAGACAGATGGTGGAGCTCTTAATAAGATAGGAAATGTGGGAGGAGGAGCAGGCTTAGGGAAA
GGGGTAAATAATGAATTCAGTTTTAAACTAACAGACTTGAAGTACTTGTGGGGTCTGCTAGGATCCCAGACTCTCTAACCCCAATTTCTTCCAGTCCCTA
TCTCCTTCCCCACATGTGACCCAGCCCCTGCTTTTCTCTACCCACTCTTCTATAGGGCCTCTCTTGTCCCGCATCCTTCTGAATCCTCCCAAACACACCC
AA

155

TTGTGTGTCAAAGGAAATGTCTTCAAATAAGAACTAGACAGAAGCATTCTCAGAAACTTATTTGTGATGTGTGCCCTCAACTAACACAGTTGAACCTTT
CTTTTGACACAGCAGTTTGGAAACACTCTTTTTGTAGAATCTACAAGTGGATATTTTGAGAGCATTGAAAATTTCGTTGGAAACGGGAAAAACCCCAA
ACACACCCAACACAAATCGTCGACCTGCAGGCATGCAAGCTTGGCACTGGCCGTCGTTTTACAACGTCGTGACTGGGAAAACCCTGGCGTT

156

GCGATGACATGATTACGATTCGAGCTCGGTACCCGGGGATCCTCTAGAGATTTGTGTTGGGTGTGTTTGGTTTTATGCTCATGAAAGATATCTGGGCCAG
AATTATAGAGGTGGTGTCACCGCATTTCAGTGCTGGTTGAAGACCCCAAAGCCAATGAAAATTTCCCCACATGCACTGAGTAGAAAGCAGAGATATCC
TGGGATAGAATCTTCAGGACTCCAGTATTCAAGGAGCAGCTAGATAAGGAGCCAATAAAGAAAGGTCTGAGGAGGCCCACAGCCCAGCCTCTTCCAC
CTTCTTTTTCCATAATAATCTCCATCTTCTTATTTCTACTTCCTTTCTCTGTCCCCCAACCCAAACACACCCAACACAAATCGTCGACCTGCAGGCATGCA
AGCTTGGCACTGGCCGTCGTTTTACAACGTCGTGACTGGGAAAACCCTGGCGTT

157

TGTTTGGTTGGTTTGTTTTTCACACAAAGAAATGATAAATTCTTGAGGTGATGGATAATCCTATTTACTCTGATGTGATTATTATGCATTGTATACCAGTAT
ACCAAAATTTCTCATATACCCCATAAATATATGCAACACCTACTATGTACCCATAAAAATTTAAAATTAAAATCATCTAACAAGTAGATTAAAGCACTATAG
TTTTGGGGTTGTATACTTACTACTGTCCCTCTCCTGACCAAGACTCTCCTTGCTGTACTTCCCTTTAGCATAACTGTGGAATTTGTCTCCAGGCTCTACTT
TTCCCAAACACACCCAACACAAATCTCTAGAGGA

158

TTGGTTGGGTAGATCACCTGAGGTCAGGAGTTTGAGACCAGCCTGGCCAACGTAGCAAACCCCGTCTCTACTAAAAATACAAAAATTAGCCAGGCGTG

GTGGTGCGTGCCTGTAATCCCAGCTACTCGGGAGGGTAAGGCATGAGAATTGCTTGAACCTGGGAGACAGAGGCTGCAGTGAGCTGAGATCATACCA
CTGCACTCCTGCCTGGGAGACAGAGCGAGACTCTGTCTCAAAAAAAAAAAAAAACCAAACACACCCAACACAAATCGTCGACCTGCAGGCATGCAAG
CTTGGCACTGGCCGTCGTTTTACAACGTCGTGACTGGGAAAACCCTGGCGTT

159

GTGGGGGGGTTTGCACAGCCTTGTAAATACTGGGGTACTCTACATGCCCTGAGCACCTGAAAAATTTAGTAAATATTTAGACGTCAACTGGTTAAAATG
ACATTAACTAAAACCTTTTTGAACATGCTATTGGGATAGGGGATGGGTTATGGTATAAATTTACTGGCTGCTATTTAAATGAAAAGAATTACAATGCACTT
AATGTTCATTCTAATCAAGTGATGCTAGAAGATTGAATTTCAGACTGTAAATTTATAACAAAACCAAACCAAACACACCCAACACAAATCGTCGACCTG
CAGGCATGCAAGCTTGGCACTGGCCGTCGTTTTACAACGTCGTGACTGGGAAAACCCTGGCGTT

160

GGTGGTGGGGAAATTTATAAAGAAAAGAGGTTTAATTGGCTCATGGTTCCGTGGGCTGTACAGGAAGCATGGCTGGGGAGACTGCGGGAAACTTCGAT
TCATTGAAAAAGGTGAAGAGGAAGGAGGCACATCTTACATGGCTGGAGCAGGAGAAAGAGTGAAGGTAGAGATTCTACACACTTTTAAACAACCACA
TCCCATGAGAACTCACTCACTGTCACACCACAACCAAACACACCCAACACAAATCGTCGACCTGCAGGCATGCAAGCTTGGCACTGGCCGTCGTTTTA
CAACGTCGTGACTGGGAAAACCCTGGCGTT

161

GGATTGACATGATTACGATTCGAGCTCGGTACCCGGGGATCCTCTAGAGATTTGTGTTGGGTGTGTTTGGTGTGTTTGGGTGGGTTTTTGAGGTCAGGA
GATCGAGGCCATCCTGGCTAACACTGTGCAACCCTGTCTCTACTAAAAATACAAAAAATTAGCCGGGCGTGGTAGCGGGTGCCTGTAGTCCCAGCTAC
TCGGGAGGCTGAGGCAGGAGAATGGCGTGAACCCAGGAGGCGGAGCTTGCAGTGAGCCAAGATAGCCACTGCAGTCCAGCCGGGGTGACAGAGCG
AGACTCCGTCTCAAAACAAAAAAAGGGGGAAAAAACAAACCAAACACACCCAACACAAATCGTCGACCTGCAGGCATGCAAGCTTGGCACTGGCCG
TCGTTTTACAACGTCGTGACTGGGAAAACCCTGGCGTT

162

TTGTTGTTTTTTAATAAGTGAGGAACAGACTCTTGGAAACTGTTTTTTCCTAGTGGCTTACCCTGTGCTTGGAATATTTCTTGAATATTTTGTGAATATTTA
AGTGACGTGGAGGCAACATTTATATTTGAAGTTTTATCCTCCTCCAAACACACCCAACACAAATCGTCGACCTGCAGGCATGCAAGCTTGGCACTGGC
CGTCGTTTTACAACGTCGTGACTGGGAAAACCCTGGCGTT

163

TGGGTTTTTGACTAATAACATGGTTACATCCAGACAAGCTGATTGTAAGTCAAAAAAATACTGTAAATTGAAAAGCATTTAATGCCTGGATAAACCCATT
GTAAAGTCAACAAATTGTACTTTGAACCATTGTAAACTTTCTGTAGGATAGTCACCCTACAGAAAGTAAAACTCAAGTTGGCCTTGGAGTTCACCCATC

ACAGGGGAGGGCCAGAGGGCCAGTCACTTGGGCACCCATCACAGAGGAGGGCCAGTCACTGAGATGAAATGAAATAAAAATAAGAAATGTTATAGGA
ACCCATGAGTGAAAGTCCAGAGGTGAGGTTGGCTTAAATATTAGTTTAATCCAGTGATTCTTGTTCTATTTCTGTATGATTCTCTTTGCTCCTCTTCAGAT
CCTTTCATCCTGAGGCAGGTATTATGATAGTTACAGTAGTTCCAGGTCTCATATCCAGTTAAAACAATGTCCAGGAGAAGAGTAAAGTTCTCAGCAACC
CACCACCAAACACACCCAACACAAATCGTCGACCTGCAGGCATGCAAGCTTGGCACTGGCCGTCGTTTTACAACGTCGTGACTGGGAAAACCCTGGC
GTT

164

TTGGTGGTTTTAAACCTCTTTACATTGAAATGAGAAGCTACTTGGAGCAAACTGTTCCTCTCTGCAAAAGTAAGGATAATGGTATCCACAATGTGGCCT
CTGACCCAGCTGTACATTTCCTACTTTCTTATCAGTGAAAATAATCAATTGACTTCTCTATTAACATTTTTTAAAAAACTAATGTCCAAAAACGAGAAAAT
CTGTTTTCAGTAGCAAAACTTATTTCTGATGTGGAAAGATCGTCAATTCTTCCTAAAAACAACACCAAACACACCCAACACAAATCGTCGACCTGCAG
GCATGCAAGCTTGGCACTGGCCGTCGTTTTACAACGTCGTGACTGGGAAAACCCTGGCGTT

165

TGGTGGTTTTTCTAGAATGAACAACCTTGAAAATGTGTTTCTGTGCCTATTATATGGACATTGCTTATCAAATCAGCAAGACCGTTTTCCTCAAAATACTT
TTGTTTTAATAGCATAATCTAGTAATAAAGACAACACATGCTCATTGAGTAAAATTCAAACAATATAAACTATAAAAAAAAACACCAAACACACCCAAC
ACAAATCGTCGACCTGCAGGCATGCAAGCTTGGCACTGGCCGTCGTTTTACAACGTCGTGACTGGGAAAACCCTGGCGTT

166

TGTGTCCACTCAGGGTTAAATGGATTAAGGGCGGTGCAAGATGTGCTTTGTTAAACAGATGCTTGAAGGCAGCAGGCTTGTTAAGAGTCATCACCAAT
CCCTAATCTCAAGTAATCAGGGACACAAACACTGCGGAAGGCCTCAGGGTCCTCTGCCTAGGAAAACCAGAGACCTTTGTTCACTTGTTTATCTGCTG
ACCTTCCCTCCACTATTGTCCCATGACCCTGCCAACCCCCAACCAAACACACCCAACACAAATCGTCGACCTGCAGGCATGCAAGCTTGGCACTGGCC
GTCGTTTTACAACGTCGTGACTGGGAAAACCCTGGCGTT

167

GGGGTACGACGGCAGTGCAGCTTGCATGCCTGCAGGTCGACGATTTGTGTTGGGTGTGTTTGGTTTTTGGTTTCAGATTCAAAGTCACTATGACTCTGA
ATCTGAAATATTAATCAAACTTCTAAGACTCGACCAAGTTCTCCACCACACCCCAAACACACCCAACACAAATCTCTAGAGGATCCCCGGGTACCGAG
CTCGAATTCGTAATCATGGTCATAGCTGTTTCCTGTGTGAAATTGTTATCCGCTCACAATTCCACACAACATACGAGCCGGAAGCATAAAGTGTAAAGCC
TGGGGTGCCTAATGAGTGAGCTAACTCACATTAATTGCGTTGCGCTCACTGCCCGCTTTCCAGTCGGGAAACCTGTCGTGCCAGCTGCATTAATGAATC
GGCCAACGCGCGGGGAGAGGCGGTTTGCGTATTGGGCGCTCTTCCGCTTCCTCGCTCACTGACTCGCTGCGCTCGGTCGTTCGGCTGCGGCGAGCGGT
ATCAGCTCACTCAAAGGCGGTAATACGGTTATCCACAGAATCAGGGGATAACGCAGGAAAGAACATGTGAGCAAAAGGCCAGCAAAAGGCCAGGAA

CCGTAAAAAGGCCGCGTTGCTGGCGTTTTTCCATAGGCTCCGCCCCCCTGACGAGCATCACAAAAATCGACGCTCAAGTCAGAGGTGGCGAAACCCG
ACAGGACTATAAAGATACCAGGCGTTTCCCCCTGGAAGCTCCCTCATGCGCTCTCCTGTTCCGACCCTGCCGCTTACCGGATACCTGTCCGCCTTTCTCC
CTTCGGGAAGCGTGCCGCTTTCTCATAGCTCACGCTGTAGGTATCTCAGTTCGGTGTATGTCGATCGCTCCAAGCTGGGCTGTGTGCACTAACCCCCCG
TTCAGCCCGACCGCTGCGCCTTATCCGGTAACTATCGTCTTGAGTCCAACCCGGTAAGACACGACTTATCCCCCCTGGGCAGCATCCCTGGTAACAGGA
TTAGCAAAGCGAGATATGTAGGCGGTGCTACAGAGTCTTGGAGTGGGGGCCTAACTACGGTACACTAGAAGAACAGTATTTGGATCTGCGCTCTCCGC
AACCCGTTACCTTCTGAAAAAGAAATGGTAGGGCTTG

168

GGTGTGGTGAAACTTGGTCGAGTCTTACAAGTTTGATTAATATTTCACATTCTAGTCATAATGACTTTGAATCTGAAACCAGAAACCAAACACAGCCGA
AAGGAAACGTCGACCTGGACGAGGGCAAGGTTGTTGGTGATTGTCGTTGATAAAATTTTCGAGAGCGAGAACCTCCCCAAT

169

TTGTTTTGTTAGGTTTTGGGGTATAAGTGCAGTTTTGTTACACACATATATTGTGTGGTGGTGAAGTCTGGGCTTTGAGTGTACCTGTCACTCGAATAGT
GAACATTGTACCCAACAGGTGATTTATCAACCACACACCAAACACACCCAACACAAATCGTCGACCTGCAGGCATGCAAGCTTGGCACTGGCCGTCGT
TTTACAACGTCGTGACTGGGAAAACCCTGGCGTT

170

TTTTGTTGGGCCAACTTTATACTTCAGCATTAAGTTTAAGTCTGCTAAATTTCTAACTTGTTTCAGAGTTGTTAGATTTTATTTTGAGGTCTATTTCCAATT
GCTTTGGTATCCATGACCCTTACAACAAATTAGACATTTACTGAATGCTTATTTTTCACTATCCTTAAGTCAGTACATTTCAGGGTAGAAGTTATATCAAG
CACATGAAAAGTCTTGAGTAGCTTCATTTATAAATACTCCCATATATAAATATGCTTAAAAGTTTGATTTTTAGAAACAGTTGTGTGTTTTTAAAAACCAA
ACCCCCCAAACACACCCAACACAAATCGTCGACCTGCAGGCATGCAAGCTTGGCACTGGCCGTCGTTTTACAACGTCGTGACTGGGAAAACCCTGGC
GTT

171

GTGGTGGGGGCCGAGGCAGGCAGATCACAAGGTCAGGAGTTTGAGACCAGCCTGACCAACATGGTGAAACCCCGTCTCTACTATAAATACAAAAACT
AGCCAGGCATGGTGACACACGCCTATAATTTCAGCTACTTGGGAGGCTGAGGCAGGAGAATCGCTTGAACCCGGGAGGCAGAGGTTGCAGTGAGCCG
AGATTGTGCCATTACAATCCAGCCTGGGCGACAGAGTGAGACTCCGTCTCGGCAAAAAAACCCCAAACACACCCAACACAAATCGTCGACCTGCAGG
CATGCAAGCTTGGCACTGGCCGTCGTTTTACAACGTCGTGACTGGGAAAACCCTGGCGTT

172

TTTTGTTGGGCCAACTTTATACTTCAGCATTAAGTTTAAGTCTGCTAAATTTCTAACTTGTTTCAGAGTTGTTAGATTTTATTTTGAGGTCTATTTCCAATT

GCTTTGGTATCCATGACCCTTACAACAAATTAGACATTTACTGAATGCTTATTTTTCACTATCCTTAAGTCAGTACATTTCAGGGTAGAAGTTATATCAAG
CACATGAAAAGTCTTGAGTAGCTTCATTTATAAATACTCCCATATATAAATATGCTTAAAAGTTTGATTTTTAGAAACAGTTGTGTGTTTTTAAAAACCAA
ACCCCCCAAACACACCCAACACAAATCGTCGACCTGCAGGCATGCAAGCTTGGCACTGGCCGTCGTTTTACAACGTCGTGACTGGGAAAACCCTGGC
GTT
173
GGAGCCGAGGTGGGTGGATCACTTGAGGTCAGGAGTTTAAGACCAACCTAACCAACATGGCGAAACCCCGTCTCTACTAAAAATACAAAAATTAGCC
AGGCATGGTGGTGGGCGCCAGTAATACTGGCTACTCAGGAGGCTGTGGCAGGAGAATCCCTTGAACCCAGGAGGGGGGGTTGCAGTGAGCCGAGATC
GCACCACTGTACTCCAGCTTGGGTGACAGAGCAAGACTCCAACTCAAAAAAAAAGAAAGAAAAGAAAAGAAAATTGTGTGGAATTACAGATTTTAGTT
TTTTGTTTGTTTTTTCTAAAAAAAAAAAACAAACACTTTTCCTAATGTATTACAAAAAGACGCCCCAAAAATGTTGTGGAAAAAATTTATAATACTAAAA
AAAAATAGTAGGCCAGGCAGGCACCAAACACACCCAACACAAATCGTCGACCTGCAGGCATGCAAGCTTGGCACTGGCCGTCGTTTTACAACGTCGT
GACTGGGAAAACCCTGGCGTT
174
TGTTTTTGTGGTCCCTCACTTTCCTCCCCATTTGCATCTGTAGGGCACTTTATTTTAAATGCTGGATTCTTTTGCTCCAGAAAGTGGGAACTGGTGGGGA
GGAGGGCCATGGATCTTTTGCTGTCAAAATAGGTTAAAGTCAAAGAGTCTTCATGGACACAGCTGTAAACCAGTGTTAGTTGTCCATATTGGCAGTGTG
TCCTTGTCTCATTGCCTTGAAATTTAGAGTTAAAGCAGGATTAAGCTTTTTTGTTTCTTAGTTTTGAAAAAATAACCTTTTTTATTCATTGTAATAACTTTT
CTTTCACCACCTCAGAGTTTCTCAGTGGACATAGCTCATCTTCCTTTACACCTTTTGCTTCTCTCAGGCCAGTGAGAGACTGTCTTATTTTGAACACCAC
CCAAACACACCCAACACAAATCGTCGACCTGCAGGCATGCAAGCTTGGCACTGGCCGTCGTTTTACAACGTCGTGACTGGGAAAACCCTGGCGTT
175
TGTGAGCCATGGTGTAAAGTCCAATTAACTGCCTTTAAAACAAGAGTAAGGACAAATTATAGATAGAGATTTCCAATTCAAGAATTTCTTTAATTTCTGA
GAGACAGGGTCTCACTCTGTTGCCCAGGCTGGAATGCAATGGCGCAATCATGGCTCACTGCAGCCTCGACCTCCTGGGCTCAGGCAATCCTCTCACCG
CACCCCCCCCCCCCAAACACACCCAACACAAATCGTCGACCTGCAGGCATGCAAGCTTGGCACTGGCCGTCGTTTTACAACGTCGTGACTGGGAAAA
CCCTGGCGTT
176
GGGTTTGTTCGCTTACTTTGGTAAAGGGAAGGGAGGGATGTGTCCCGTACTATGTGGCAAAGTGAACTTAATACAAGCCAATGCTCTCTCCCCCATCAC
GTACTTGTGATAGATGACCTCTTTACTTGTGAGTGGTTGCACCCTTACAATGATAAGTCTGGACTGCCTTCCTGCTGACAAGGCAATGACGAGCAGCTT
CTTGTCCCTTGTTCACCAGCTGGCGTGTGGCCTTGGACCTGAGACATTTTCTTTCTTTTTTTGTTTTTGTTTTTGTGTTTGAGATGGGGTTACTGACTTGT

TGCCCTCTCTGGAGTGCAATGGAACAGACTAGGCTCACCTATGCCTCCCCCTCCTGAGTTT
177
AGACTTCAGTTGCTCACTTTCAATAATGGATAGAAGATCTAGGTAAAAGATAACTAAGGAAATAGAGGATTGGAACAAAAACACCAAACACACCCAAC
ACAAATCGTCGACCTGCAGGCATGCAAGCTTGGCACTGGCCGTCGTTTTACAACGTCGTGACTGGGAAAACCCTGGCGTT
178
GTTTACATTGTTGCTTGAAAGGTTCTAATATCATCTAATATCTCAGTAGCTATGGAATATGAATAAAACTAAAGAGAAGTCATCCATTCTATTTTCTCTAAA
TTTTAAAACCACTCCAGATGGCTCCTCATTTATGGACACACAAACAGAAAAAAATCAAAATAAAATATAATGACAACTCAGCATTTGATATGCAAATAA
GTACATAGTTTTGAAAAATAGTTACTCTGCAACAGATGCGCAACATTTAAAAATATCTTTGTGACAATCTTAATAAATTTTAAAAATATAGACCAACCAC
ACCAAACACACCCAACACAAATCGTCGACCTGCAGGCATGCAAGCTTGGCACTGGCCGTCGTTTTACAACGTCGTGACTGGGAAAACCCTGGCGTT
179
GGTGTGGTTTATTTGCCAAAGCTAAGGACGCACACTGTGACACAGCCTCAGGAGGTCCTGATGACATGTGTCCAAGGTGGGTCGGGGCACAGCTTGG
TTTTACACATTTAGGGAGACAAGAGACATCAATCAATATATTTAAGAAGTACATTGGTTTGTCCAAAAAGGTGGGTGCCAGGCCTCTGAGCCCAAGCTA
AGCCATCATAACCCCAACCCCAAACCAACACCAAACACACCCAACACAAATCGTCGACCTGCAGGCATGCAAGCTTGGCACTGGCCGTCGTTTTACA
ACGTCGTGACTGGGAAAACCCTGGCGTTA
180
GCCATATGTACCTGATTACGATTCGAGCTCGGTACCCGGGGATCCTCTAGAGATTTGTGTTGGGTGTGTTTGGGGTGTGGTTTATTTGCCAAAGCTAAGG
ACGCACACTGTGACACAGCCTCAGGAGGTCCTGATGACATGTGTCCAAGGTGGGTCGGGGCACAGCTTGGTTTTACACATTTAGGGAGACAAGAGAC
ATCAATCAATATATTTAAGAAGTACATTGGTTTGTCCAAAAAGGTGGGTGCCAGGCCTCTGAGCCCAAGCTAAGCCATCATAACCCCAACCCCAAACCA
ACACCAAACACACCCAACACAAATCGTCGACCTGCAGGCATGCAAGCTTGGCACTGGCCGTCGTTTTACAACGTCGTGACTGGGAAAACCCTGGCGT
T
181
GCATTGACATGATTACGATTCGAGCTCGGTACCCGGGGATCCTCTAGAGATTTGTGTTGGGTGTGTTTGGGGGGTTGTGTGTGTATGTGATTTTGTGTGT
GCACTAATACACATGTAGGAGACCAAGGCAGGAGAATCACATATCCAAAGCCAAGCTGCCTACCAGGTTGCACAGTAAATTACAGGTCATGGCAGGCT
AAAGAGGTACCCTGTCTTTAAAACCCAAACAAAAGAAATCTATATCTTACCAACAACCCCAAGAAACAACCATTTTGCACACCACATTTCATATGTATA
AGTTAATTAAATGACATTGAAACACACACATTAGTTTTTCTACTGTATTTTTTTTTTTACATGAGTCATAGATAGTTAACTCAGGCACTATTCCTGGCCATA
CATTCCCATGTCCCATTGCTTCTTCAACAGCTATTTCTTTAACAATGTTAGTGATTTCTATCATCTCGCTATTATTGATAACACTATAATTAAAAAGCAGAC

GATTCTCACAAAAACACCAAACACACCCAACACAAATCGTCGACCTGCAGGCATGCAAGCTTGGCACTGGCCGTCGTTTTACAACGTCGTGACTGGG
AAAACCCTGGCGTTTTCGGGGAAAT

182

GGACATGACATGATTACGATTCGAGCTCGGTACCCGGGGATCCTCTAGAGATTTGTGTTGGGTGTGTTTGGTGGTTTTTTTGTGGAGATGGGATTTTGCC
ATGTTTCCCAGGCTGGTCACAAATTCCTAGGCTCAAGCAATCTTCCCACCTTGACCTCCCAAAGTGTTGGAATGATAGGTGTGAGCCACCGTGCCTGGC
CCATATGGTTATTTTTGGTGAAGAAATTTTCAGGTACATGGTCATCCTCCACACTAGTTAGCCATTAAGTTTTCACTTAAAGCTCTACATCAGGGATCATA
GCAAGTCAGGTTAAACAGTAAGACAAGCAAAGGCTTTAGCTTACTTAGAAAAGCAACTCAAATACTGTAGTTTCCTGTTTCTAGAAGGCAGGCTAAAT
ATAATTGCATGGTAAGTTCTAAGTTTTCAAAATAGTATCAGAAAAAAATAAGGAGGCAAGGGTAGTGACAGCATTTGGATTGACCTTGTAGATTCTGATAC
TGTAAACTTTCTTAATTTTAATATTTCACTCTCTTAAGATTTGTCCACAACATTTCATATTCTAAATAAACCACATCCTTCCATTACTGGAAGGGATATTAG
AAATCAGTGCATTCAACCAACACCCAAACACACCCAACACAAATCGTCGACCTGCAGGCATGCAAGCTTGGCACTGGCCGTCGTTTTACAACGTCGT
GACTGGGAAAACCCTGGCGTT

183

GCAATGACATGATTACGATTCGAGCTCGGTACCCGGGGATCCTCTAGAGATTTGTGTTGGGTGTGTTTGGGTTGTTGGTTTATTTATTGTAAGTACACTGT
AGCTGTCCTCAGACACTCCAGAAGAGGGAGTCAGATCTCATTACGGATGGTTGTGAGCCACCATGTGGTTGCTGGGATTTGAACTCCTGACCTTCGGA
AGAGCAGTCGGGTGCTCCTTCCCACTGAGCCATCTCACCAGCCCATGGTGTAAATTCTTAAAGGTAAGAAGGGAGATAGTCCCTTTCTCTGCCTACCAT
ACTACCCCACTCCCAACATCCCTAAGCAGAGGGTCAAAACATAAATGGCTTCATCTTGTTAATGTAACTTTAAAAGCCAGGCGGTCACTTTTCAGAGGA
GATAGCTGCACTCCTACAGTTAGTTGCCTTGGCTAGTCCAGTTTCCACTGGAGTGCGGCAGGACTGCGGTCTGTCGCTGCTCTCCGTGCTCTTCCCAGT
GAGGGAGACCCCGGCCTTCATTCACACTCAGCTTCTGGGCATCAACTATGTGAGGAGAGTCTATGAATCTTACAAGAAAGCAACCAGCTCTCAGAAAA
CACTCACCAAACACACCCAACACAAATCGTCGACCTGCAGGCATGCAAGCTTGGCACTGGCCGTCGTTTTACAACGTCGTGACTGGGAAAACCCTGG
CGTT

184

TTTGTGGGGGTCGAGGTGGGTGGATCACCTGAAGTCAAGAATTTGAGAGCATCCTGGCCAACATGGCAAAACCATGTCTCTACTAAAAATACAAAAAC
TAGCCAGGCATGATGGTGCATGCCTATAGTCCCAGCTACTTGGGAGGCTGAGGCAGAAGAATCGCTTGAACCCTGGAGGCAGAGGTTGCAGTGAGCC
AAGGTCGTGCCACCGTACTCCAGCCTGCGCAACAGAGTGAGACAACATCCCCCCAAAAAAAAAAAATCAGGAAAACAGTTCATGATCTGAAGGAAAA
AAAACAACCAAACACACCCAACACAAATCGTCGACCTGCAGGCA

185

TGTTAGTTTGTTGACCCCTAAGATAGATGATGCTAATATACATTTCTTGGTATAAACTTTCCTGGCATTCCCTAAACTAACTCTTTTTAGGCATGGTCTATT
AAAAGTATAGTAAAAGGCACTGATAACTTTCTCATCACAGTGAACAGGCAACCTACAGAATGGGAGAAAATTTTTGCAATCTCCCCCCACCACCAAAC
ACACCCAACACACCAAACACACCCAACACACCAAACACACCCAACACAAATCGTCGACCTGCAGGCATGCAAGCTTGGCACTGGCCGTCGTTTTACA
ACGTCGTGACTGGGAAAACCCTGGCGTT

186

GACCCCTAAGATAGATGATGCTAATATACATTTCTTGGTATAAACTTTCCTGGCATTCCCTAAGCTAACTCTTTTTAGGCATGGTCTATTAAAAGTATAGTA
AAAGGCACTGATAACTTTCTCATCACAGAGAACAAGCAACCTACAGAATGGGAGAAAATTTTTGCAATCTCCCCCCACCACCAAACACACCCAACAC
ACCAAACACACCCAACACACCAAACACACCCAACACAAA

187

TTTGTGGGGGTCGAGGTGGGTGGATCACCTGAAGTCAAGAATTTGAGAGCATCCTGGCCAACATGGCAAAACCATGTCTCTACTAAAAATACAAAAAC
TAGCCAGGCATGATGGTGCATGCCTATAGTCCCAGCTACTTGGGAGGCTGAGGCAGAAGAATCGCTTGAACCCTGGAGGCAGAGGTTGCAGTGAGCC
AAGGTCGTGCCACCGTACTCCAGCCTGCGCAACAGAGTGAGACAACATCCCCCCAAAAAAAAAAAATCAGGAAAACAGTTCATGATCTGAAGGAAAA
AAAACAACCAAACACACCCAACACAAATCGTCGACCTGCAGGCATGCAAGCTTGGCACTGGCCGTCGTTTTACAACGTCGTGACTGGGAAAACCCTG
GCGTT

188

TGTTTGTGGGGCCTGGTTACCAGGTGACAGTTGCTGTAAGTTTTAATCCAACTTCTCTATTGAAAAATACACTATCTTGCAACTGCCTTGAAGCTGCCAC
AAAAATACATTTTTAAAGGAGTAATAAACAAATATCAAGAAATAGCCTTCATAAAATTGTCAGTATTAAAACCCCCCCCAAACACACCCAACACAAATC
GTCGACCTGCAGGCATGCAAGCTTGGCACTGGCCGTCGTTTTACAACGTCGTGACTGGGAAAACCCTGGCGTT

189

TTTTCTGTTCCTGTGTTAGTTTGCTAAGGATAATGGCCTCCAGCTCCATCCATATCCCTGCAAAGGACATGATCTCATTCTTTTTTATGGCTGTATACTATT
CCACAGTGTATATGTAGTATATATTCTCAAATATGTGAGTGTACACCTCCTTTCCTTTGGGCCCAACTGTCTCTATTTTGTCTGGGTCTGACAAAAGAGAT
TTCATCTTGGTATATGAAACATTCACTGCACAATATGCAAACTTTTTGCCTGTCTGATGTTGACACTATATCATCCAAGACTATTCTTATGTCAATGTCCTT
AAGACAGTTCAGGAAAAAATGGATGTAGTACAGAAACATATGGAAAATTGTCTCACAGGATCCCACCTCATTTTATGCAATGCAGGTTCTAGGTTCCTT
ATTAGAAAGTGTATCTCCTTCTTCTCTTCTGACTGTATGTCATTCACCTAGACCATGAAGGGTTTATATTTGACCATTCCTCAAGTCAGAAATTGATCCTG
ACATAGGCATAACATTAAAAGCCCCACCCCCCAAACACACCCAACACAAATCGTCGACCTGCAGGCATGCAAGCTTGGCACTGGCCGTCGTTTTACAA

CGTCGTGACTGGGAAAACCCTGGCGTT
190
GTTTACATTGTTGCTTGAAAGGTTCTAATATCATCTAATATCTCAGTAGCTATGGAATATGAATAAAACTAAAGAGAAGTCATCCATTCTATTTTCTCTAAA
TTTTAAAACCACTCCAGATGGCTCCTCATTTATGGACACACAAACAGAAAAAAATCAAAATAAAATATAATGACAACTCAGCATTTGATATGCAAATAA
GTACATAGTTTTGAAAAATAGTTACTCTGCAACAGATGCGCAACATTTAAAAATATCTTTGTGACAATCTTAATAAATTTTAAAAATATAGACCAACCAC
ACCAAACACACCCAACACAAATCGTCGACCTGCAGGCATGCAAGCTTGGCACTGGCCGTCGTTTTACAACGTCGTGACTGGGAAAACCCTGGCGTT
191
GGGTTGTATACTTGTTTCTGGAAGTTTTCCACCTCATTTCTGGCTGAAGTGAATCCTTGAAGAACCAGGAGGGCTGAGCAGAAAACAAATATTTGCCAC
TGCTTTCAGATCCATCCCTCTGTGGGAACAATCTGGAGAAACTGCCTTTTTCATATTTTCAGTAAAAAAAAATTCCATATTCATGCCATTTCCTGTATTATG
CAAAACCCCAACCAAACACACCCAACACAAATCGTCGACCTGCAGGCATGCAAGCTTGGCACTGGCCGTCGTTTTACAACGTCGTGACTGGGAAAAC
CCTGGCGTT
192
GGTGTGGGTTCTCTCATCATCACTGAGCTCCATCCAGGGAACCTGGGATATCTCCCCCAAGGGAAAATAAAATGGAGCCAATTAGCCCATCCCTCAAAC
ATCTATACAGACCCCTGTGTAAACAACACACACACAGCCAGCCAGGCCTTGAGGGTCATCAGGAGGGATAGGCCTCTGTCTCCCTCACCCAGGGGACC
TCAGTATAGTACAAGGAACCTAAAAAAGCAACTGTCGATCCCTCCATTCTGCTGGTATGGGGGTGTATGCCTATAGTCTTGTATTCAAGAGGCTGAGGC
AGGAGGATGGCTAGCCTGGGTTGCAAAAGAAAAGACATCACACTTTTCTGCTACCCATAGGTAGCCTTTTTCTTTCTCTCTCTCTCTCTCTCTCTCTCTC
TCGCTCTCGCTCTCCTTGCTTTCTTAATCAGGACTGAATCTAGAATACTTCAAAGACTTTGTCATGCTCTTATGGGAATTTCTTTTCATTCATCACAGATA
CACATGGTGGTAACAGAGATGCAGGGATAGCTGAGCTTGTGAAGGCCCAAGCTCAGTTCCCACAACAACACCCAAACACACCCAACACAAATCGTCG
ACCTGCAGGCATGCAAGCTTGGCACTGGCCGTCGTTTTACAACGTCGTGACTGGGAAAACCCTGGCGTT
193
GGGGTTGTGTGTGTATGTGATTTTGTGTGTGCACTAATACACATGTAGGAGACCAAGGCAGGAGAATCACATATCCAAAGCCAAGCTGCCTACCAGGTT
GCACAGTAAATTACAGGTCATGGCAGGCTAAAGAGGTACCCTGTCTTTAAAACCCAAACAAAAGAAATCTATATCTTACCAACAACCCCAAGAAACAA
CCATTTTGCACACCACATTTCATATGTATAAGTTAATTAAATGACATTGAAACACACACATTAGTTTTTCTACTGTATTTTTTTTTTTACATGAGTCATAGAT
AGTTAACTCAGGCACTATTCCTGGCCATACATTCCCATGTCCCATTGCTTCTTCAACAGCTATTTCTTTAACAATGTTAGTGATTTCTATCATCTCGCTATT
ATTGATAACACTATAATTAAAAAGCAGACGATTCTCACAAAAACACCAAACACACCCAACACAAATCGTCGACCTGCAGGCATGCAAGCTTGGCACTG
GCCGTCGTTTTACAACGTCGTGACTGGGAAAACCCTGGCGTT

194

GTGGTGGGGGCCGAGGCAGGCAGATCACAAGGTCAGGAGTTTGAGACCAGCCTGACCAACATGGTGAAACCCCGTCTCTACTATAAATACAAAAACT
AGCCAGGCATGGTGACACACGCCTATAATTTCAGCTACTTGGGAGGCTGAGGCAGGAGAATCGCTTGAACCCGGGAGGCAGAGGTTGCAGTGAGCCG
AGATTGTGCCATTACAATCCAGCCTGGGCGACAGAGTGAGACTCCGTCTCGGCAAAAAAACCCCAAACACACCCAACACAAATCGTCGACCTGCAGG
CATGCAAGCTTGGCACTGGCCGTCGTTTTACAACGTCGTGACTGGGAAAACCCTGGCGTT
